# Dynamical footprints enable detection of disease emergence

**DOI:** 10.1371/journal.pbio.3000697

**Published:** 2020-05-20

**Authors:** Tobias S. Brett, Pejman Rohani

**Affiliations:** 1 Odum School of Ecology, University of Georgia, Athens, Georgia, United States of America; 2 Center for the Ecology of Infectious Diseases, University of Georgia, Athens, Georgia, United States of America; 3 Department of Infectious Diseases, College of Veterinary Medicine, University of Georgia, Athens, Georgia, United States of America; University of Oxford, UNITED KINGDOM

## Abstract

Developing methods for anticipating the emergence or reemergence of infectious diseases is both important and timely; however, traditional model-based approaches are stymied by uncertainty surrounding the underlying drivers. Here, we demonstrate an operational, mechanism-agnostic detection algorithm for disease (re-)emergence based on early warning signals (EWSs) derived from the theory of critical slowing down. Specifically, we used computer simulations to train a supervised learning algorithm to detect the dynamical footprints of (re-)emergence present in epidemiological data. Our algorithm was then challenged to forecast the slowly manifesting, spatially replicated reemergence of mumps in England in the mid-2000s and pertussis post-1980 in the United States. Our method successfully anticipated mumps reemergence 4 years in advance, during which time mitigation efforts could have been implemented. From 1980 onwards, our model identified resurgent states with increasing accuracy, leading to reliable classification starting in 1992. Additionally, we successfully applied the detection algorithm to 2 vector-transmitted case studies, namely, outbreaks of dengue serotypes in Puerto Rico and a rapidly unfolding outbreak of plague in 2017 in Madagascar. Taken together, these findings illustrate the power of theoretically informed machine learning techniques to develop early warning systems for the (re-)emergence of infectious diseases.

## Abstract 2

This study develops an operational algorithm for the detection of the (re-)emergence of infectious disease. The authors illustrate its utility by successfully applying it to four (re-)emerging threats—mumps, pertussis, dengue and plague, providing early warning that could enable intervention measures.

## Introduction

Outbreaks of infectious diseases continue to surprise and evade public health control policy. This is due to a combination of (1) the reemergence of familiar vaccine-preventable infectious diseases, such as mumps [1], measles [2], and pertussis [3]; (2) the evolution of resistance to antimicrobials, including methicillin-resistant *Staphylococcus aureus* (MRSA) [4], malaria [5], and extensively drug-resistant tuberculosis (XDR TB) [6]; (3) pathogen range expansion driven by anthropogenic changes in land use [7] and climate [8]; and (4) the emergence of novel pathogens from a zoonotic reservoir, such as HIV [9], severe acute respiratory syndrome coronavirus (SARS-CoV) [10], and Ebola virus [11]. In addition to their burden on human morbidity, mortality, and the associated social and economic toll, the existential threat posed by (re-)emerging infectious diseases is increasingly recognized [12].

To foreshadow such threats, field and laboratory approaches have focused on surveillance of potential zoonotic hosts [13], the detection of "viral chatter" in sequence data collected from putative emergence hotspots [14], laboratory characterization of viruses with pandemic potential [15], biogeographic approaches to identify risk zones [16], and the use of phylogenetics to pinpoint animal reservoirs [17]. We submit that an important dimension to predicting pathogen (re-)emergence is to exploit epidemiological incidence reports. In reality, a diversity of mechanisms can drive increases in transmission that underpin disease emergence or resurgence. These include pathogen evolution leading to evasion of immunity [18, 19], host adaptation [20], immune waning [21], changes in population immune profile [22], environmental change [8], declining vaccine uptake [23], and changes in contacts [24]. This mechanistic uncertainty, coupled with sparsity of data, impedes the prospects for inference-based forecasts (e.g., by fitting a transmission model). Previously, statistical approaches have been developed focusing on characteristics of the outbreak size distribution [2, 25–27]. Though promising, generalizing these methods requires overcoming the need for (1) a sufficiently large number of independent outbreaks for reliable statistical estimation and (2) well-defined transmission chains, which is often not possible. Here, we propose a mechanism-agnostic approach that harvests information contained in longitudinal epidemiological data.

**Fig 1:**
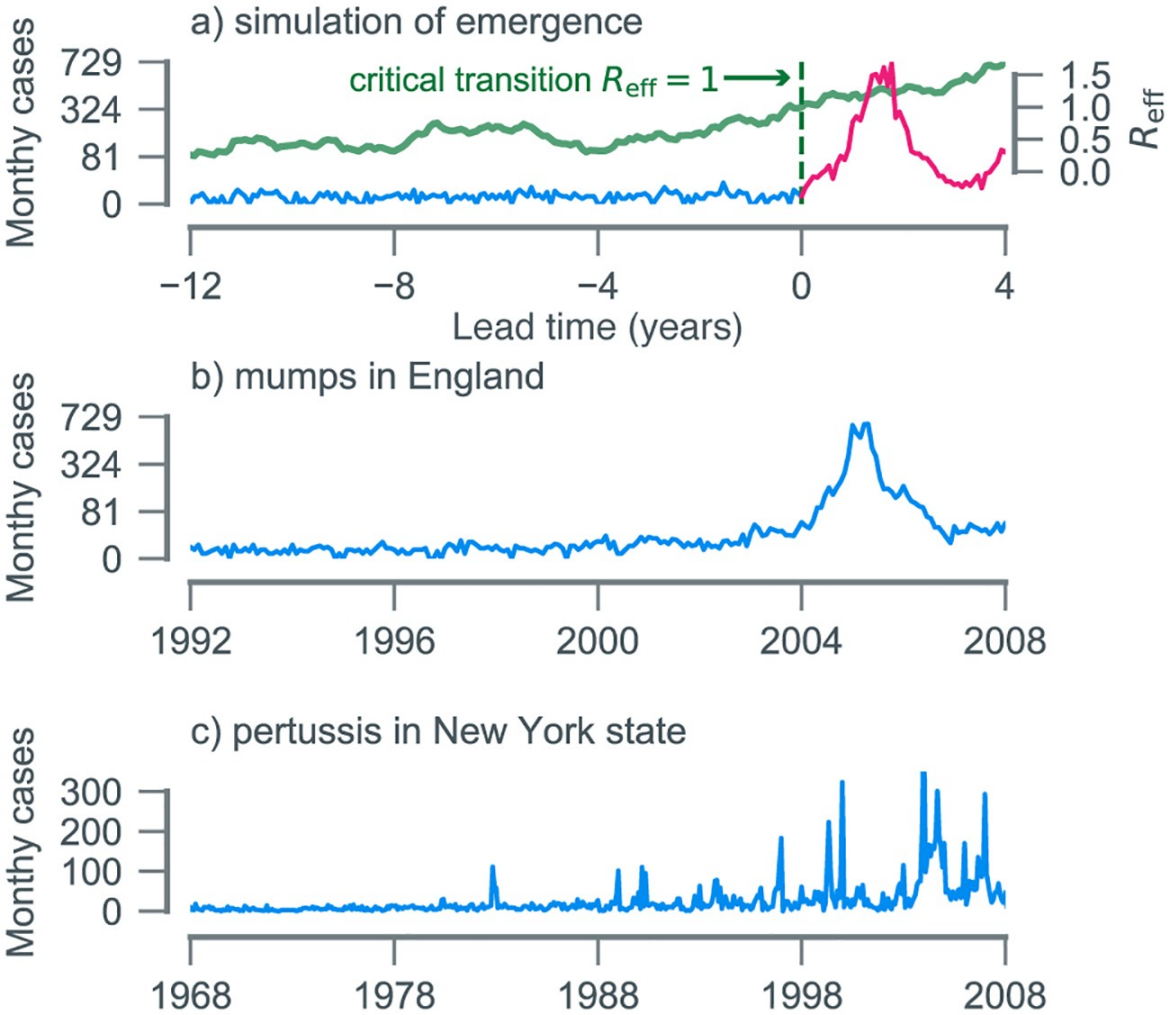
(a) Simulation of an emerging disease in a population of 10^5^ susceptible individuals. After 12 years, *R*_eff_ crosses the epidemic threshold, and a large outbreak is triggered. (b) Monthly clinically confirmed cases of mumps in England. Sixteen years after mass vaccination began in 1988, England experienced a large outbreak of mumps, primarily among university-aged individuals. (c) Monthly reported cases of pertussis in New York state (USA). Beginning in the late 1970s, various states (including New York) began experiencing resurgent outbreaks, in spite of high reported vaccine coverage levels. Data and code used to generate this figure can be found at https://doi.org/10.5281/zenodo.3713381.

**Fig 2:**
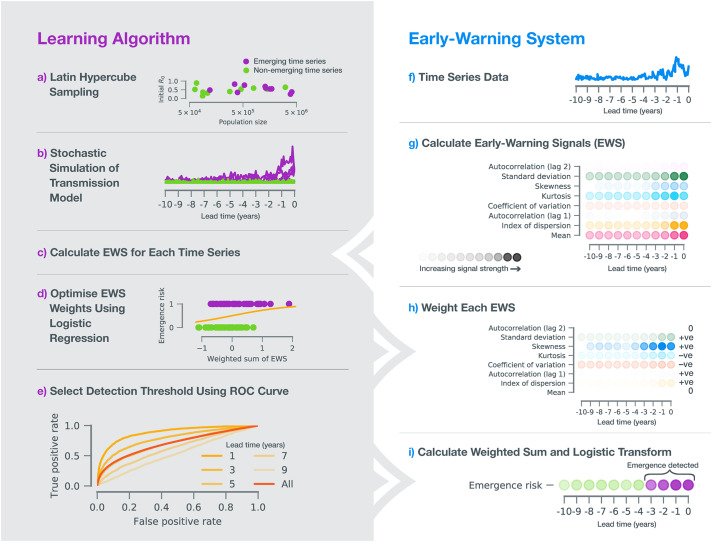
Demonstration of algorithm design and operationalization. (a) To create a training dataset, 10^5^ unique parameter sets were selected according to Latin hypercube sampling. Half were emerging (*R*_0_ had an increasing trend with endpoint *R*_0_ = 1), and half were not (no overall trend). (b) For each parameter set, a stochastic simulation algorithm was used to produce a time series of weekly case reports, subject to observation error. (c) For each simulated time series, 8 EWSs were calculated (see panel g). (d) The measure of emergence risk, defined as $D_{t}=[1+\text{exp}(-\sum_{i=1}^{8}w_{i}\Theta_{i,t}-w_{0}{)]}^{-1}$, was fitted to the emerging and nonemerging simulated time series using penalized logistic regression (see Methods), generating EWS weights ${\left\{w_{i}\right\}}_{i=1}^{8}$ used in *D*_*t*_ (fitted values are listed in S5 Table). Our algorithm detects emergence whenever *D*_*t*_ exceeds the detection threshold *c*. (e) Using the fitted weights (panel h), we parameterized *c* by minimizing the classification error using the ROC curve. In panels (f–i), we present a step-by-step outline of how, for any time series data, our fitted algorithm functions as an early warning system. EWS, early warning signal; ROC, receiver-operator characteristic.

In general, disease (re-)emergence requires a systematic increase in the expected number of secondary cases due to an infectious individual, which is quantified by the effective reproductive number (*R*_eff_) [28]. Specifically, as the threshold *R*_eff_ = 1 is crossed, the system undergoes a transcritical bifurcation, and sustained chains of transmission become possible (Fig 1A). Dynamical systems theory identifies statistical footprints of such a critical transition ("critical slowing down") [29]. These footprints are reflected in trends in the statistical moments of time series data, such as the autocorrelation and standard deviation [30, 31], as the transition is approached. Prior theoretical findings [30–32] and tests on simulated data [33, 34] support the premise of this approach and identify candidate statistical moments. The key challenge, however, is operationalizing these statistical features to serve as early warning signals (EWSs). In particular, given a time series, we need to (1) quantify emergence risk through time from a collection of EWS and (2) establish a threshold for detection of emergence. Here, we accomplish these by use of transfer learning, i.e., training a learning algorithm on simulated time series data to create a classifier that can subsequently detect emergence in incidence data (Fig 2; see Methods). The advantage of using a transfer learning approach is the identification of a generic measure of emergence risk that is robust to uncertainties in the underlying epidemiological dynamics.

## Results

### Statistical learning algorithm

We used a stochastic transmission model to generate 10,000 emerging and nonemerging time series (Fig 2B). To ensure robustness to parametric uncertainty, each time series was the result of a unique parameterization according to Latin hypercube sampling (Fig 2A; see S4 Table for ranges). For each trajectory, 8 time-varying EWSs (${\left\{\Theta_{i,t}\right\}}_{i=1}^{8}$) were calculated (Fig 2C). To classify disease emergence, logistic regression was carried out on the ensemble of EWSs to assign a weight to each signal (Fig 2D). We defined a summary measure of time-dependent emergence risk as the logistic transform of the weighted sum of our EWS, $D_{t}=[1+\text{exp}(-\sum_{i=1}^{8}w_{i}\Theta_{i,t}-w_{0}{)]}^{-1}$, with a range between 0 and 1 (see Methods for details). In this algorithm, emergence is predicted at any time *t* when *D*_*t*_ > *c*, where *c* is a threshold (Fig 2I). We identified this threshold by minimizing classification error, using the receiver-operator characteristic (ROC) curve (Fig 2E). We evaluated the performance of the detection algorithm as a function of lead time (the period of time before the outbreak) using the area under the ROC curve (AUC) statistic (Fig 2E). Further details of the learning algorithm are given in the Methods. The learning algorithm is designed such that the weighted EWS and detection threshold (Fig 2F–2I) may be applied to incidence data without further fitting.

We found that a composite of EWSs was a better predictor of emergence than any individual EWS (S3A Fig). Most weight was assigned to the skewness, the kurtosis, and coefficient of variation. Indeed, training on just these 3 features yields near-optimal performance (S4 Fig). Interestingly, individually, these are not the best performing EWS (in fact, the kurtosis and coefficient of variation are the 2 worst; S3A Fig). The learning algorithm exploits imbalances in these 3 EWS to detect emergence, assigning negative weight to the kurtosis and coefficient of variation (Fig 2H and S5 Table). A practical implication of this is that the performance of the algorithm is insensitive to population size (S3 Fig). That is, the outcome of application to case counts or incidence data (normalized by population size) is identical.

### Sensitivity to mechanism of emergence

We examined whether performance of our algorithm was sensitive to mechanisms (e.g., waning immunity and pathogen evolution) underlying (re-)emergence that are associated with different patterns of increase of *R*_eff_ (for details, see S1 Text). We retrained the learning algorithm on simulated datasets consisting of only (1) concave (*d*^2^*R*_eff_ / *dt*^2^ < 0; waning immunity) and (2) convex (*d*^2^*R*_eff_ / *dt*^2^ > 0; evolution) trends. Surprisingly, the EWS weights obtained by fitting to simulated data that comprised both mechanisms of increase performed best, comparable to training on concave/convex data alone (S5 and S6 Figs).

We performed a similar comparison using data simulated from a model with multiple time-varying parameters, in addition to *R*_eff_ (which drives the transition), to assess their confounding effects (S7 Fig). As might be expected, the presence of covariates reduced performance; however, we again found that the EWS weights obtained by fitting to simulated data without such covariates performed comparably to the optimal fit with covariates.

### Mumps case study

To test the performance of our detection algorithm, we carried out 2 case studies on re-emerging vaccine-preventable childhood diseases. Our first challenge was to anticipate the reemergence of mumps. In England, infant mumps vaccination started in 1988 and coincided with a rapid reduction in incidence (Fig 1B). This period of low transmission was interrupted in 2004 to 2005 by outbreaks reported across the country, primarily among university-aged individuals [35]. We examined whether our mechanism-agnostic approach could have anticipated these outbreaks.

**Fig 3:**
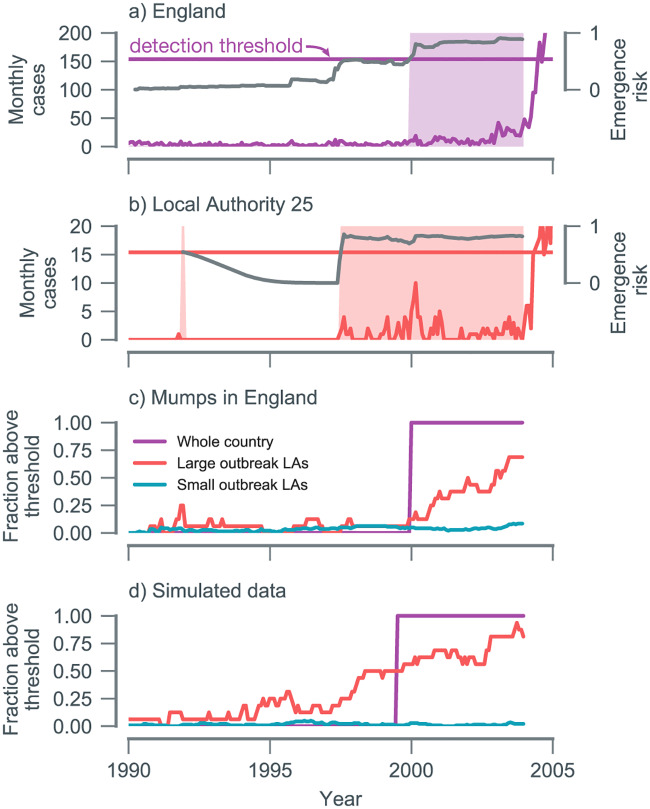
Performance at detecting the 2004 to 2005 mumps outbreak in England. (a) Laboratory-confirmed cases of mumps in England. The predicted emergence risk (*D*_*t*_; gray line) on the national level goes above the detection threshold (*c*; horizontal line) around 2000. Shaded backgrounds indicate *D*_*t*_ > *c*. (b) LA 25 had the most cases of all LAs and the longest lead time. Lines and shading correspond to the same quantities as shown in panel (a). (c) As the lead time decreases, the fraction of LAs above the threshold increases. Most of the localities above the threshold prior to 2004 had large outbreaks (> 92 cases). (d) Numerical simulation of a country with an administrative structure matched to England produces qualitatively similar results as shown in panel (c). Data and code used to generate this figure can be found at https://doi.org/10.5281/zenodo.3713381. LA, local authority.

We used the EWS weights (trained on simulated data) to calculate our emergence risk measure, *D*_*t*_. We additionally explored the potential impact of spatial scale on the predictability of emergence by calculating *D*_*t*_ at both the national level (Fig 3A) and for each local authority (LA) (Fig 3B and S8–S16 Figs). At the national level, the 2004 to 2005 outbreak was successfully anticipated, with a lead time of approximately 4 years (Fig 3A). At the local level, from 1998 onwards, we observed an increasing number of LAs exceeding the detection threshold, shifting from a baseline average of 4.6 LAs per week before 2000 to 23 detections per week at the start of 2004 (S20 Fig).

We additionally categorized localities into those that experienced a sizeable outbreak in 2004 to 2005 and those with small epidemics (S17 Fig). As shown in Fig 3C, locations with small outbreaks had a much lower emergence detection frequency, which we interpret to mean a low false positive rate. To dissect whether differences across spatial scales may result from data aggregation alone, we generated a simulated time series for each LA assuming spatial independence, ensuring the number of emerging and nonemerging time series matched the number of LAs with large and small outbreaks, respectively. As shown in Fig 3D, there was qualitative agreement between simulated and mumps data. One discrepancy was that the number of LAs above the threshold in 2000 was lower for mumps. We speculate this is due to additional spatial heterogeneity in mumps transmission in England (beyond our emerging/nonemerging categorization), perhaps with a small set of LAs (e.g., those with large urban student populations) serving as foci.

Our measure of emergence risk should not be conflated with a prediction of future outbreak size or its imminence. *D*_*t*_ quantifies whether the system is approaching *R*_eff_ = 1. The final outbreak size is determined by additional factors, such as the susceptible population size and the number initially infected [26]. We found no association between *D*_*t*_ and epidemic size for large outbreak LAs (S18 Fig). Similarly, although there is no theoretically derived relationship between *D*_*t*_ and the time of an expected outbreak, we observed a negative association between an LA’s outbreak size and the detection time, defined as the last time that *D*_*t*_ < *c* prior to the outbreak (Spearman’s *ρ* = −0.66; S19 Fig). This may be because larger outbreaks occur in LAs with larger susceptible populations, which are more likely to experience repeated "sparks" prior to the outbreak, hence providing a more reliable probe of the system’s state.

### Pertussis case study

**Fig 4:**
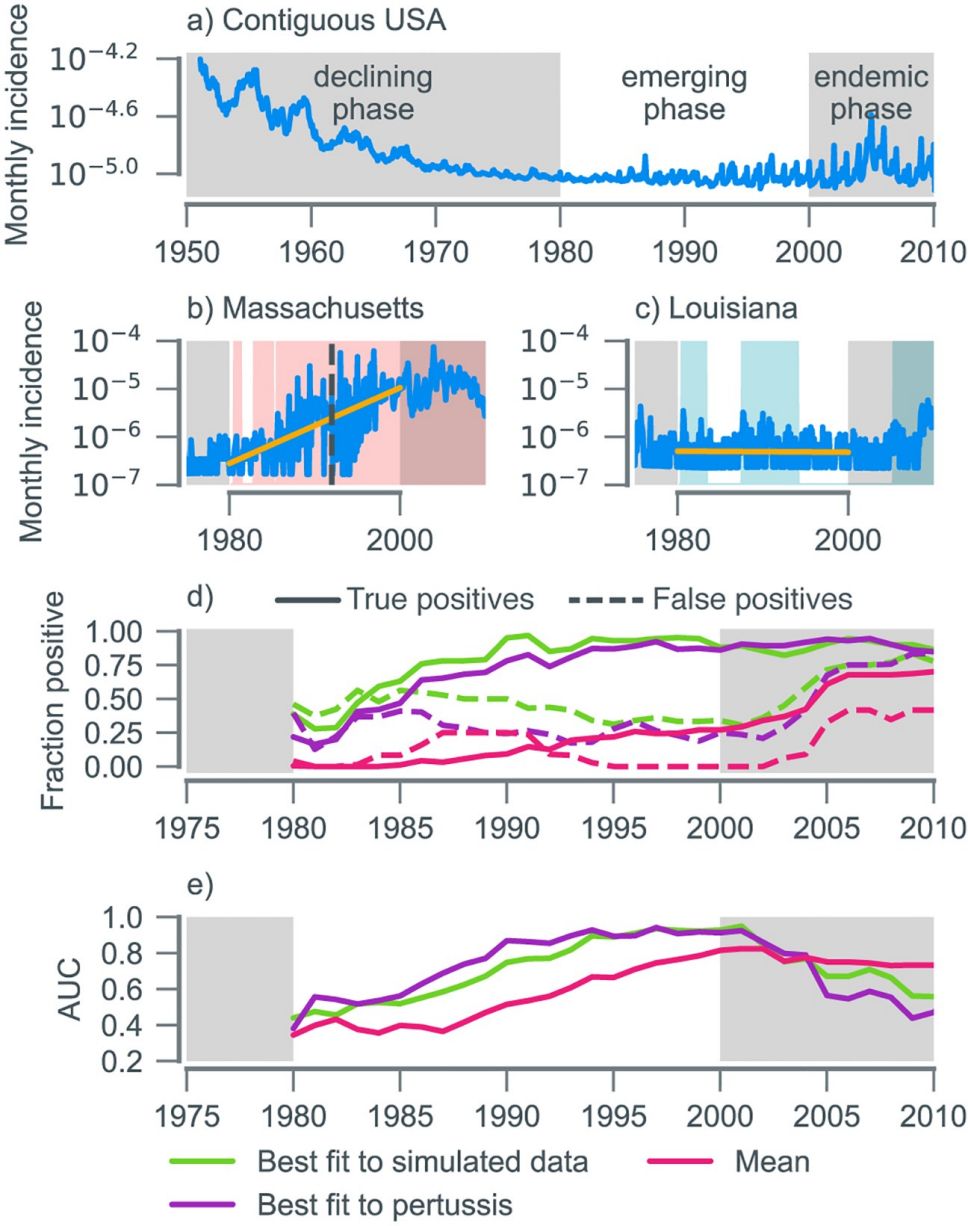
Performance at detecting pertussis reemergence in the contiguous US. (a) After a declining phase, pertussis incidence began increasing in the late 1970s. We focus our analysis on the emerging phase (1980 to 2000; white background). (b,c) Pertussis reemergence has been geographically uneven. Incidence in Massachusetts (b) increased through the 1980s and 1990s before plateauing in the 2000s, whereas in Louisiana (c), incidence was flat after 1980 until an uptick in the 2000s. Timing of first large outbreak (annual incidence over 5 cases per 10^5^) is shown by vertical dashed lines. Shaded backgrounds indicate detections of emergence (pink and blue shading correspond to true and false positives, respectively). (d) Fraction of emerging states (solid lines) and not emerging states (dashed lines) above the detection threshold using just the mean incidence (pink), *D*_*t*_ with EWS weights fitted to simulated data (green), and *D*_*t*_ with EWS weights fitted to pertussis data (purple). (e) AUC for the 3 emergence risk measures in panel (d). The fit to pertussis provides the upper bound on the ability of our set of EWSs to classify pertussis emergence in the US. Fitting to the simulated dataset gets remarkably close to this upper bound. Data and code used to generate this figure can be found at https://doi.org/10.5281/zenodo.3713381. AUC, area under the receiver-operator characteristic curve; EWS, early warning signal.

Our second case study focused on the resurgence of pertussis in the US. In most states, pertussis incidence declined throughout the 1950s and 1960s until it reached a nadir in the mid-1970s [36]. Since then, however, this trend has reversed. By the late 2000s, annual reported incidence in many states had reached levels not seen since the 1960s (Fig 4A). The mechanisms underlying this resurgence remain contested [21, 37, 38]. A striking feature of pertussis reemergence has been its geographic unevenness [36]. In some states, reemergence did not take place until the mid-2000s (Fig 4C), whereas in others, resurgence occurred early, and incidence has plateaued (Fig 4B). We restrict our analysis to the period from 1980 to 2000. We were prevented from performing a similar analysis to mumps because of the substantial variation in the timing of the first large outbreak in each state (S24–S26 Figs), which precludes the aggregation of detections in the same manner (for details, see S27 Fig). Instead, we used regression analysis to identify which states experienced reemergence (37 states, including Washington, DC) and which did not (12 states). We challenged our algorithm—based on the EWS weights fitted to the simulated data—to predict whether resurgence occurred in each state (Fig 4D and 4E and S24–S26 Figs). Earlier detections of resurgence imply better performance. In Fig 4D, it is shown that from 1990, almost 100% of states experiencing resurgence exceed the detection threshold. For those states not experiencing resurgence, from 30% to 50% were above the threshold; reasons for these detections likely vary on a state-by-state basis. Some detections (such as in Delaware and Oklahoma) can be attributed to isolated sporadic outbreaks in under-vaccinated communities, not associated with the national trends [39]. In other states (e.g., Wyoming, see panel Q of S26 Fig), the algorithm may be detecting a late resurgence not identified by the linear regression.

To quantify algorithm performance, we calculated the time-varying AUC (a measure of diagnostic ability), which crossed the nominal value of 0.8 around 1992 and continued to increase as the year 2000 was approached (Fig 4E). As with simulated data, *D*_*t*_ outperforms any individual EWS, as depicted in Fig 4D and 4E using the mean (the performance of the remaining 7 EWS is shown in S28 Fig).

To obtain an upper bound on the ability of EWS to classify pertussis emergence, we retrained the learning algorithm on pertussis data (Fig 4E). There is remarkable similarity in performance relative to the model fitted to simulated data (Fig 4E). Reassuringly, the fit to pertussis data assigns most weight to the same 3 EWSs: the skewness, kurtosis, and coefficient of variation (S29 Fig). Taken together, these findings suggest that our algorithm trained on simulated data can be reliably applied to incidence data. Note that there were more positives (true and false) when the algorithm is fitted to simulated data, indicating a lower detection threshold (Fig 4D). This likely arose because demographic parameter ranges in the simulated data were chosen to mimic those of England rather than the US.

### Dengue and plague case studies

**Fig 5:**
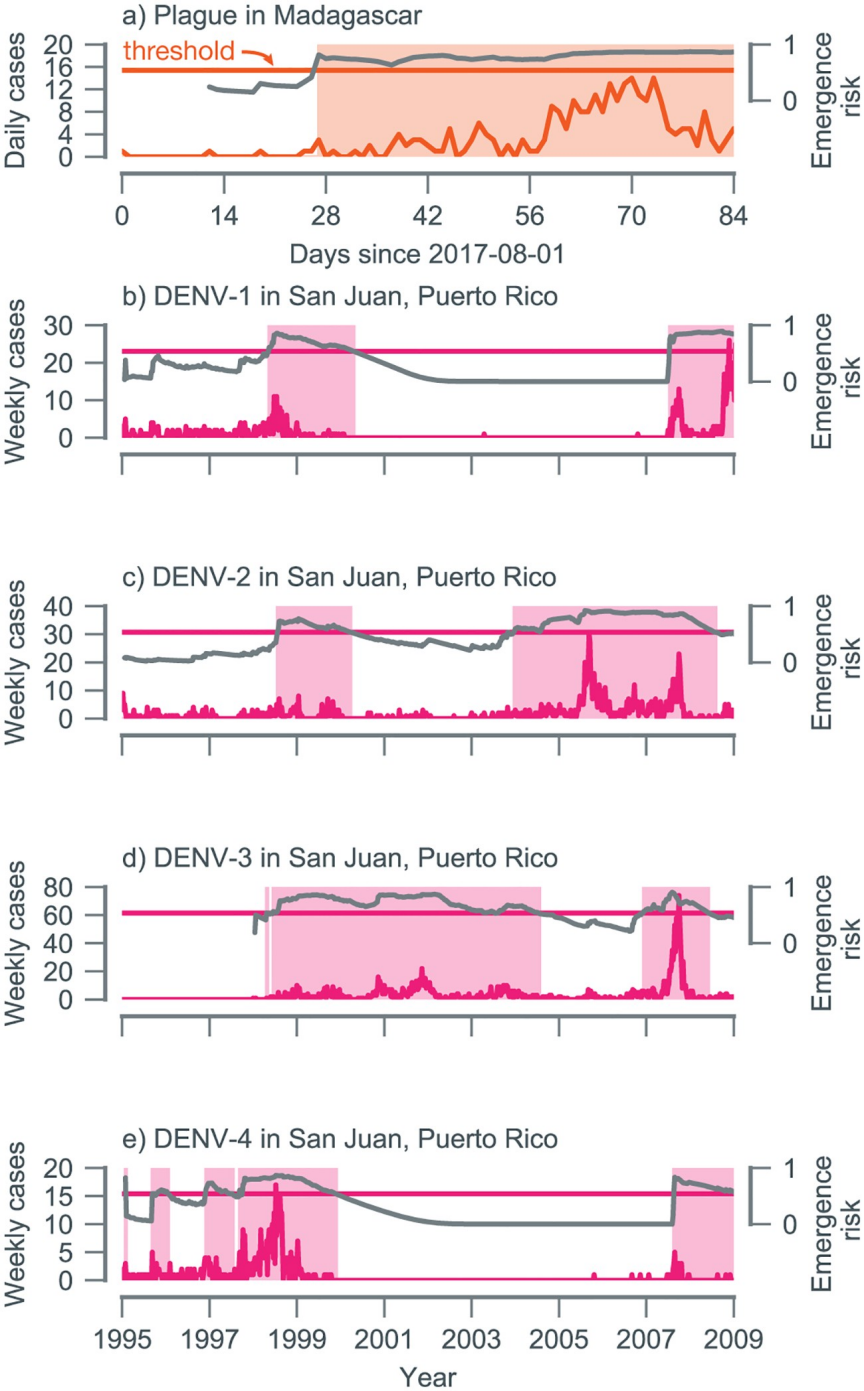
Performance at detecting outbreaks of (a) bubonic plague in Madagascar and (b-e) dengue in Puerto Rico. EWSs are calculated using different temporal resolutions: daily case counts of bubonic plague and weekly case counts of serotyped DENV infections. (a) For plague, emergence risk (*D*_*t*_) increases above the detection threshold on day 27. (b) The algorithm detects a small uptick in DENV-1 cases around 1998. The 2009 outbreak is detected 1 year in advance; detection prior to this is impossible because of an absence of cases. (c) For DENV-2, the algorithm makes 2 detections: 1 in 1998 that was not associated with a large outbreak and 1 at the start of 2004 that preceded the takeoff of the 2006 outbreak by 18 months. (d) For a long period (roughly 1999 to 2004), *D*_*t*_ is above the detection threshold for DENV-3, centered on a small outbreak in late 2001 (with a weekly peak of 22 confirmed cases). The much larger 2008 DENV outbreak is detected with a lead time of about 6 months. (e) The outbreak of DENV-4 in late 1998 was detected with a lead time of around 4 months. The reintroduction of DENV-4 in 2007 triggered a further detection. Data and code used to generate this figure can be found at https://doi.org/10.5281/zenodo.3713381. DENV, dengue virus; EWS, early warning signal.

In addition to the reemergence of these 2 vaccine-preventable childhood diseases, we tested the performance of our algorithm (fitted to the synthetic dataset described in the Methods) on outbreaks of 2 vector-borne diseases, bubonic plague, and dengue. Compared with the mumps and pertussis examples, the 2017 Madagascar plague outbreak took place over a much shorter timescale, driven by increasingly favorable climatic factors [40, 41]. Examining the daily case reports of bubonic plague, the emergence risk crossed the detection threshold 27 days after the first reported case, a lead time of around 30 days before the outbreak in late September (Fig 5A).

The epidemiological dynamics of dengue virus (DENV) serotypes are complex; dengue infection leads to lifelong serotype-specific immunity and a transient period of serotype-transcending protection [42, 43]. In Puerto Rico, these interactions led to a sequence of extinctions and recolonizations for DENV-1 (2000 to 2008), DENV-3 (1995 to 1998), and DENV-4 (2000 to 2008), with only DENV-2 in constant circulation over the entire period (Fig 5B–5E). Our algorithm, which is designed to detect trends in transmission from incidence data, successfully anticipated outbreaks of DENV-2 (2006) and DENV-3 (2008) that followed periods of modest but continuous transmission. These outbreaks were the result of shifts in the immunological profile of the population following the replenishment of the serotype-specific susceptible pool. This caused a systematic increase in *R*_eff_ culminating in a transcritical bifurcation. For DENV-2, our algorithm made 2 sustained detections: 1 in 1998 that was associated with a small outbreak and 1 at the start of 2004 that preceded the takeoff of the 2006 outbreak by 18 months (Fig 5C). For DENV-3, the 2008 outbreak was anticipated approximately 6 months ahead of time (Fig 5D). As expected, because of the absence of transmission during extinction periods, our algorithm was unable to anticipate sudden reintroduction events (e.g., DENV-1 in 2007).

### Transfer learning using the pertussis dataset

As a final test of our transfer learning approach, we applied our detection method fitted to pertussis data (using only the 3 most important EWS discussed earlier) to mumps, plague, and dengue on the national level (S31 and 32 Figs). Remarkably, there was next to no change in performance when compared with fitting to simulated data. The plague outbreak was detected on the same day, whereas for both the mumps and dengue outbreaks, the detections were within 2 months of each other. There was a reduction in positives (true and false) for mumps at the local level; however, there was still an appreciable increase in detections in LAs with large outbreaks before 2004 (S31C Fig). This robustness to the choice of training data underscores that our transfer learning–based approach is not reliant on the specifics of the simulated dataset. Instead, its success stems from the generic statistical properties of incidence data across disease emergence contexts.

## Discussion

At first glance, it may appear surprising that a singular detection method is able to detect emergence in the diverse contexts studied in this paper. However, underlying all these systems are dynamical commonalities inherent to the disease transmission process: as the reproductive number increases, the feedback effect of each infectious case on subsequent transmission is enhanced ("critical-slowing down"). In a similar vein to a recent study on the elimination of measles [44], our work shows that there is a "canonical path" for diseases emerging via increases in the reproductive number and that it can be found using statistical learning methods.

Although we have endeavored to design our detection method such that it is broadly applicable, specific usage of our algorithm necessitates decisions that cannot be made in a context-agnostic manner. In particular, all detection methods face a trade-off between reducing false positives (via a higher detection threshold) and false negatives (a lower threshold). For the mumps example, because of the rarity of outbreaks, our optimized threshold resulted in a relatively large total number of false positives prior to the 2004 outbreak, which could conceivably result in detection fatigue among end users. This threshold was arrived at by minimizing the classification error, assigning an equal cost to the false negative and false positive rates. The number of false positives can be reduced dramatically by assigning a greater cost to false positives than false negatives (S21 Fig), however, with an unavoidable reduction in the lead time provided by detections of the 2004 outbreak. The appropriate detection threshold is conditional on the potential human, economic, and political costs of a missed outbreak (which would be greater for dengue than for mumps, for example), and requires an assessment that can only be made by public health authorities.

The early warning system proposed here is likely to operate successfully for acute infectious diseases in which the approach to the critical transition (i.e., *R*_eff_ → 1) is gradual. This may result from (1) steady shifts in a population’s immune profile due to either waning immunity [21] or turnover in “antigenic seniority,” as documented in influenza [22], and (2) the accumulation of mutations that facilitate immune evasion [45] or host adaptation [20]. Instances in which the transition is abrupt (for example, the introduction of a reassortant influenza virus [46] or the de novo spillover of an easily human-to-human transmissible pathogen from a zoonotic reservoir [16], such as Ebola virus) cannot conceivably be predicted using approaches that rely on statistical trends in incidence data.

Here, we have demonstrated how ideas from the science of critical slowing down, implemented via a machine-learned detection method, point the way forward for early warning of disease (re-)emergence. Although our fitted model performed remarkably well in each case study, the gravity of confident declaration of disease (re-)emergence demands further scrutiny on the ideal choice of training data and the predictive impact of alternative fitting methodologies. Given the importance of anticipating such events and identifying appropriate preemptive steps to mitigate their toll, the adoption of a multiplicity of approaches is warranted [13–17]. Progress will likely require a combination of activities including pathogen discovery, characterization, and increased zoonotic surveillance, allied to cutting edge data analytics.

## Methods

### EWSs

EWSs are indicators of approaching critical transitions in dynamical systems. Mathematically, they are defined as the moments and correlation functions of the fluctuations away from a stable equilibrium [29]. As the critical point is approached, the strength of restorative forces decreases, and the magnitude of the fluctuations increases. These changes are captured in various different unique EWSs, Θ_*i*,*t*_, which are indexed by the subscript *i* = 1…*n*. We considered *n* = 8 EWS: the mean, standard deviation, coefficient of variation, index of dispersion, skewness, kurtosis, and autocorrelation at lags 1 and 2. For *R*_0_ < 1, the disease-free equilibrium is stable; previous theoretical studies have shown that as *R*_0_ approaches 1, there are detectable trends in the EWS [30–32].

Operationally, EWSs are calculated from a single epidemiological time series (${\left\{X_{s}\right\}}_{s=t_{0}}^{T}$; either case reports or incidence). We grouped weekly case reports into 4-week totals, informed by previous findings [33]. Estimators for each EWS were constructed by substituting any expectations, $E[f(X_{t})]$, in its mathematical definition with an exponentially weighted moving window average,

$$E[f(X_{t})]\approx \frac{\sum_{s=t_{0}}^{t}e^{-\lambda (t-s)}f(X_{s})}{\sum_{s=t_{0}}^{t}e^{-\lambda (t-s)}}.$$ (1)

The decay rate *λ* was specified by the half-life (the length of time for the exponential weight to half in magnitude), *t*_1/2_ = ln(2)/*λ*. Preliminary studies found using exponential weighting in the moving averaging, rather than the more common uniform window, performed better at detecting emergence in the synthetic dataset. The estimators for each EWS are listed in S1 Table.

### Synthetic dataset

This dataset was generated using a stochastic SEIR model [47]. The model incorporates demographic and environmental stochasticity as well as reporting error. Simulations were performed using the next reaction method [48]. The population size fluctuated via births and deaths, with a stable mean population size *N*_0_ and a mean life expectancy of 75 years. Transmission due to external sources occurred at rate *ζ*. Latent and infectious periods were assumed to be exponentially distributed with a mean of 13 days and 6 days, respectively, values appropriate for mumps [47]. Infection-derived immunity was assumed to be complete and lifelong. Time series were of length 20 years: a 10-year transient period with fixed parameters (not used for training the learning algorithm), followed by *T* = 10 years with varying parameters. For each time series, *i*, *R*_0_ followed a unique stochastic trajectory with initial value $R_{0}^{\left(i\right)}(0)<1$. We generated 2 types of data. Time series for an emerging pathogen were generated using a Brownian bridge process, with curvature determined by parameter *κ*. For these data, $R_{0}^{\left(i\right)}(T)=1$. We also generated data with no average trend in *R*_0_, such that $E[R_{0}(T)]=R_{0}^{\left(i\right)}$; these data were generated using an Ornstein–Uhlenbeck process. To ensure that $R_{0}^{\left(i\right)}(t)<1$ for all *t* < *T* for emerging and nonemerging time series and that $R_{0}^{\left(i\right)}(T)=1$ for the emerging time series, we ignored seasonality in transmission (a feature of all the diseases studied in this paper). The simulation algorithm returned time series of the reported number of new cases. These case counts were aggregated into weekly case reports, mimicking the practices of public health bodies. A negative binomial reporting error was applied to each weekly case report, with mean *ρ*. Model symbols and reaction rates are listed in S2 and S3 Tables, respectively. A total of 10,000 stochastic trajectories were generated, with an even split between emerging and nonemerging. We used Latin hypercube sampling so that each simulated time series had unique values for 5 parameters: initial population size (*N*_0_), reporting probability (*ρ*), import rate (*ζ*), the initial *R*_0_ ($R_{0}^{\left(i\right)}$), and the volatility of the Brownian random walk (*κ*). Parameter ranges for the Latin hypercube are given in S4 Table.

### Learning algorithm

As a measure of emergence risk, we used

$$D_{t}(\Theta_{t},w)={\left[1+e^{-\sum_{i=1}^{n}w_{i}\Theta_{i,t}-w_{0}}\right]}^{-1},$$ (2)

where *w*_*t*_ is the weight applied to the *i*-th EWS, *w*_0_ is the intercept, and *n* = 8. We fitted *D*_*t*_ to the synthetic dataset using logistic regression with an *ℓ*^1^-penalty ("lasso regression" [49]), treating each time point of each time series as an independent data point (see S1 Text for more details). Each data point was assigned equal importance in the fit—i.e., we did not prioritize classification accuracy for data points closer to the time of emergence. We used an *ℓ*^1^-penalty both to prevent overfitting to the training data and as a means of feature selection [49]. Our learning algorithm has 2 hyperparameters, the penalty strength *p* and the half-life *t*_1/2_ (used to calculate the EWSs), which were optimized using the AUC via 10-fold cross-validation [49] (see S1 Text). Using the optimized hyperparameters, we trained on the full synthetic dataset to get the optimum set of weights ${\left\{w_{i}\right\}}_{i=1}^{n}$ and intercept *w*_0_ for *D*_*t*_. We selected a detection threshold *c* by calculating the ROC curve—a parametric plot of type I errors against type II errors as a function of detection threshold—and finding the threshold that minimizes the sum of the type I and type II error rates. We calculated the AUC through time by grouping the dataset by reporting week and then calculating the ROC for each group separately. The AUC was calculated from the ROC curve using the trapezoidal rule. We fitted Eq 2 to both case reports data and incidence data (calculated by scaling each simulated time series by its associated population size).

### Mumps data

We sourced laboratory-confirmed mumps cases from Public Health England. Cases were disaggregated by specimen collection week and the respective LA. To preserve patient anonymity, each LA was assigned a unique integer identifier and every specimen week was shifted by the same constant. Because of the formation of new LAs during the time period, we restricted our analysis to the 157 LAs with cases of mumps prior to vaccination in 1988. We calculated emergence risk for each LA by grouping cases into 4-week reporting intervals and applying Eq 2, using the fit to the synthetic case reports. Emergence risk was calculated nationally by aggregating 4-week case reports from all LAs before calculating the EWSs.

For each LA, we calculated the outbreak size (total cases) in 2004 to 2005. To assign LAs as those with large and small outbreaks, we modeled outbreak size using a general mixture model. The model used was a mixture of 2 exponential distributions, $P(\cdot )=\sum_{i=1}^{2}\phi_{i}f_{i}(\cdot ;\lambda_{i})$, with rate parameters *λ*_1_ ≤ *λ*_2_. We fitted the model to the outbreak size data using maximum likelihood. An observed outbreak of size *o* was classified as large if ${\hat{\phi }}_{1}f_{1}(o;{\hat{\lambda }}_{1})>{\hat{\phi }}_{2}f_{2}(o;{\hat{\lambda }}_{2})$; otherwise, we classified it as small.

### Pertussis data

We obtained monthly pertussis case reports for each of the 48 contiguous states plus the District of Columbia from 1980 to 2000 [36]. Using state-level population data [50], we converted case reports data into incidence data. We performed a linear regression on the log-transformed monthly incidence data. States were classified as either emerging or not emerging based on the significance of the slope using a 1-sided *t* test. We used a significance level of 0.05.

EWSs were calculated for each state. Emergence risk was then calculated with Eq 2, using the weights fitted to the synthetic incidence dataset. Performance was assessed by calculating the AUC, using the linear regression classification as the true classification. Emergence risk was also calculated using Eq 2 with weights fitted to the pertussis data instead of simulated data. For this fit, the linear regression classification of each state was used as the target in the logistic regression.

### Plague and dengue data

Daily case counts of bubonic plague in Madagascar were obtained from [40]. Weekly serotype-resolved confirmed cases of dengue were made publicly available by the NOAA as part of the Dengue Forecasting project.

### Data availability

Data and code to reproduce results are deposited in the Zenodo repository: https://doi.org/10.5281/zenodo.3713381 [51].

## Supporting information

S1 Text Supplemental text. (PDF)

S1 Table List of early warning signals. (DOCX)

S2 Table Model symbols. (DOCX)

S3 Table Transitions of the SEIR transmission model. At the beginning of each aggregation period *C* is reset to 0.
(DOCX)

S4 Table Latin hypercube space. (DOCX)

S5 Table List of EWS weights and intercepts. Weights found by performing lasso regression on the dataset indicated in the columns with hyperparameters weeks (see S2 Fig). EWS, early warning signal.
(DOCX)

S1 Fig Time series of 20 emerging (left column) and nonemerging (right column) samples from the Latin hypercube. Top row shows trajectories of *R*_0_(*t*); bottom row shows case reports through time. Convex trajectories are generated using *κ* > 1, whereas concave use *κ* < 1. Data and code used to generate this figure can be found at https://doi.org/10.5281/zenodo.3713381.
(TIF)

S2 FigHeat maps showing mean AUC values from cross-validation for (a) incidence data and (b) case reports data.The results are largely unaffected by data type, with the same maximum, *μ*_AUC_ = 0.68, located at $t_{1/2}^{m}=156$ weeks, *p*^*m*^ = 10^−3^. The black contour indicates the region within 1 standard deviation of the maximum. The best hyperparameter values are the same for both data types, *t*_1/2_ = 156 and *p* = 10^4^. Data and code used to generate this figure can be found at https://doi.org/10.5281/zenodo.3713381. AUC, area under the receiver-operator characteristic curve.(TIF)

S3 Fig AUC through time for the complete simulated dataset. In panel (a), case counts are converted to incidence data before EWSs are calculated; in panel (b), raw case counts are used. Our measure of emergence risk, *D*_*t*_, with weights fitted to the simulated data (light green line) outperforms any individual EWS at distinguishing between emerging and nonemerging time series. Performance is only affected by data type for the mean, variance, and index of dispersion. Data and code used to generate this figure can be found at https://doi.org/10.5281/zenodo.3713381. AUC, area under the receiver-operator characteristic curve; EWS, early warning signal.
(TIF)

S4 Fig Analysis of importance of individual EWSs to performance of *D*_*t*_. (a) AUC through time when fitting *D*_*t*_ with 1 EWS left out. Color matches S3 Fig and indicates the EWSs left out. (b) Same data as panel (a) but showing only the AUC at a lead time of 3 years. EWSs are ordered from left to right based on impact on performance. Exclusion of the skewness is seen to have the most detrimental impact on performance, followed by the kurtosis and coefficient of variation. (c,d) Based on the ranking in (b), EWSs are sequentially added, with color indicating the rightmost EWS included in the fit. Including the skewness, kurtosis, and coefficient of variation is sufficient to get close to optimal performance. Calculated using incidence data. Data and code used to generate this figure can be found at https://doi.org/10.5281/zenodo.3713381. AUC, area under the receiver-operator characteristic curve; EWS, early warning signal.
(TIF)

S5 Fig AUC through time for the simulated dataset with only convex trends in *R*_0_. Case counts are converted to incidence data before EWSs are calculated. The fit to the simulated dataset with both concave and convex trends (light green) has comparable performance to the fit to just convex simulated data (dark purple). Data and code used to generate this figure can be found at https://doi.org/10.5281/zenodo.3713381. AUC, area under the receiver-operator characteristic curve; EWS, early warning signal.
(TIF)

S6 Fig AUC through time for the simulated dataset with only concave trends in *R*_0_. Case counts are converted to incidence data before EWSs are calculated. The fit to the simulated dataset with both concave and convex trends (light green) has comparable performance to the fit to just concave simulated data (dark purple). Data and code used to generate this figure can be found at https://doi.org/10.5281/zenodo.3713381. AUC, area under the receiver-operator characteristic curve; EWS, early warning signal.
(TIF)

S7 Fig AUC through time for the simulated dataset with the inclusion of covariates. Covariates included are the population size, the reporting probability, and the importation rate. Covariates vary linearly through time; initial and final values are included as independent variables in the Latin hypercube with identical ranges given in S4 Table. Case counts are converted to incidence data before EWSs are calculated. The fit to the simulated dataset with covariates (light green) has comparable performance to the fit without their inclusion (dark purple). Data and code used to generate this figure can be found at https://doi.org/10.5281/zenodo.3713381. AUC, area under the receiver-operator characteristic curve; EWS, early warning signal.
(TIF)

S8 Fig Mumps cases by local authority. The coloring indicates the classification using the GMM, with red and blue corresponding to large and small outbreaks, respectively. Shaded backgrounds indicate *D*_*t*_ < *c*. Data and code used to generate this figure can be found at https://doi.org/10.5281/zenodo.3713381. GMM, general mixture model.
(TIF)

S9 Fig Continuation of S8 Fig. (TIF)

S10 Fig Continuation of S8 Fig. (TIF)

S11 Fig Continuation of S8 Fig. (TIF)

S12 Fig Continuation of S8 Fig. (TIF)

S13 Fig Continuation of S8 Fig. (TIF)

S14 Fig Continuation of S8 Fig. (TIF)

S15 Fig Continuation of S8 Fig. (TIF)

S16 Fig Continuation of S8 Fig. (TIF)

S17 Fig Fit of the GMM to outbreak size data for mumps in England. The GMM fit identifies a boundary between large and small outbreaks at 92 cases (dashed gray line). Dots show the empirical distribution calculated using a bin width of 5. Data and code used to generate this figure can be found at https://doi.org/10.5281/zenodo.3713381. GMM, general mixture model.
(TIF)

S18 Fig Size of the 2004 to 2005 mumps outbreak against emergence risk at the start of 2004, *D*_2004_. Data and code used to generate this figure can be found at https://doi.org/10.5281/zenodo.3713381.
(TIF)

S19 Fig Size of the 2004 to 2005 mumps outbreak against the detection time. Data and code used to generate this figure can be found at https://doi.org/10.5281/zenodo.3713381.
(TIF)

S20 Fig **(a) Fraction of LAs above the detection threshold through time**. Excluding LAs for which no cases were recorded between 1990 to 2004 (32 in total) has little effect on either fraction. (b) Number of LAs above the detection threshold through time. Data and code used to generate this figure can be found at https://doi.org/10.5281/zenodo.3713381. LA, local authority.
(TIF)

S21 Fig Same as S20 Fig, however, with a higher detection threshold, *c* = 0.65. Data and code used to generate this figure can be found at https://doi.org/10.5281/zenodo.3713381.
(TIF)

S22 Fig Performance of *D*_*t*_ and each individual EWS at detecting mumps outbreaks. Weights and thresholds for both *D*_*t*_ and the individual EWS were found by fitting to the simulated training data. (a) Detection rate in local authorities with large outbreaks. We assumed that these detections are true positives. (b) False positives, i.e., detections in local authorities with small outbreaks. (c) Difference between the true and false positive rates. (d) Close up of panel (c) focusing on the last 6 years before the outbreak; the coefficient of variation, skewness, and kurtosis are not shown in this panels as these 3 EWSs performed poorly individually—echoing their performance during training (S3 Fig)—with high initial false positive and true positive rates. For all the remaining EWSs, detections increased as the transition was approached, with the fit using all EWSs, *D*_*t*_, having the highest true positive rate (a) and difference between positive rates (c and d) for most of the period post-2000. Performance of the autocorrelation (at lags 1 and 2) and the index of dispersion are close behind. Data and code used to generate this figure can be found at https://doi.org/10.5281/zenodo.3713381. EWS, early warning signal.
(TIF)

S23 Fig Fit of the GMM to annual incidence for pertussis. The GMM is fit to the annual incidence data from the years 1980 to 2012, treating each year and state as an independent sample. The fit identifies a boundary between large and small outbreaks of 6.10 cases per 10^5^ (dashed gray line). Dots show the empirical distribution calculated using a bin width of 1 case per 10^5^. Data and code used to generate this figure can be found at https://doi.org/10.5281/zenodo.3713381. GMM, general mixture model.
(TIF)

S24 Fig Emergence risk for pertussis in US states AL–KY. Colors indicate whether the linear regression analysis classified a state as emerging (red) or not (blue). Log-transformed incidence data are shown in dark blue; linear regression fits are shown in orange. *p*-Values from the linear regression analysis are shown in the panel labels. Shaded backgrounds indicate *D*_*t*_ > *c*. Black dashed vertical lines indicate the year of the first large outbreak, found using the GMM. Data and code used to generate this figure can be found at https://doi.org/10.5281/zenodo.3713381. GMM, general mixture model.
(TIF)

S25 Fig Continuation of S24 Fig for states LA–NC. (TIF)

S26 Fig Continuation of S24 Fig for states ND–WY. (TIF)

S27 Fig **(a) Fraction of states above the detection threshold for a range of detection thresholds as a function of lead time to the first large outbreak (determined using the GMM)**. Also shown are the number of states with *p* < 0.05 when using the linear regression method. (b) Because the earliest year used to calculate the EWSs is 1980, the number of states in the denominator of the fraction decreases as the lead time increases, with states that have the earliest first large outbreaks dropping out first. This leads to difficulties interpreting the results shown in panel (a) at lead times greater than 4 years. Data and code used to generate this figure can be found at https://doi.org/10.5281/zenodo.3713381. EWS, early warning signal.
(TIF)

S28 Fig Performance of different EWS at classifying pertussis reemergence in USA using incidence data. The fit to simulated data performs much better than any individual EWS and is close to the upper bound on the ability of our set of EWS to classify pertussis emergence in the US. Data and code used to generate this figure can be found at https://doi.org/10.5281/zenodo.3713381. EWS, early warning signal.
(TIF)

S29 Fig Replication of S4 Fig using pertussis data. The skewness, kurtosis, and coefficient of variation remain the most important to the performance of *D*_*t*_. Data and code used to generate this figure can be found at https://doi.org/10.5281/zenodo.3713381.
(TIF)

S30 Fig Time series of confirmed cases for DENV serotypes 1–4. Data and code used to generate this figure can be found at https://doi.org/10.5281/zenodo.3713381. DENV, dengue virus.
(TIF)

S31 Fig Replication of Fig 3 using weights trained on the pertussis dataset. Instead of training on the simulated data, we take the weights and detection threshold found by training on the pertussis data. We only included the 3 EWSs that were most important to the performance of *D*_*t*_ in the fit (see S4 Fig), namely, the coefficient of variation, the kurtosis, and the skewness. On the national level, the detection time is largely unchanged. On the local authority level, both true and false positives are reduced, indicating a more stringent detection threshold. Performance is slightly worse if the fit to pertussis including all EWSs is used. Data and code used to generate this figure can be found at https://doi.org/10.5281/zenodo.3713381. EWS, early warning signal.
(TIF)

S32 Fig Replication of Fig 5 using weights trained on the pertussis data instead of the simulated training data. We see no effect on the timing of detection for bubonic plague and a very small effect for dengue. Data and code used to generate this figure can be found at https://doi.org/10.5281/zenodo.3713381.
(TIF)

S33 Fig Comparison of performance of EWS-based detection methods with using linear regression. Linear regression is performed using the log-transformed pertussis data from 1980 to the date indicated on the x-axis. As the endpoint of the linear regression approaches 2000, the AUC approaches 1. This is to be expected, as we are comparing the performance of the linear regression with itself. The EWS-based approach fitted to simulated data tracks this performance closely until the mid-1990s, when the AUC saturates at 0.9. The EWS-based approach fitted to the pertussis data outperforms the linear regression during much of the 1980s and 90s, because of the 8-dimensional logistic model’s increased flexibility. Data and code used to generate this figure can be found at https://doi.org/10.5281/zenodo.3713381. AUC, area under the receiver-operator characteristic curve; EWS, early warning signal.
(TIF)

S34 Fig Comparison of (a) the EWS method and (b) a linear regression method at predicting the 2006 DENV-2 outbreak. The linear regression method was implemented in the same way as for the pertussis case study and was calculated using all log-transformed case reports from January 1, 1995 up to the week indicated. As can be seen by visually inspecting the curves in panel (b), even if the significance level (i.e., the detection threshold, indicated by the horizontal line) is lowered, the linear regression method performs worse at detecting the outbreak. Data and code used to generate this figure can be found at https://doi.org/10.5281/zenodo.3713381. DENV, dengue virus; EWS, early warning signal.
(TIF)

## Abbreviations

AUC: area under the receiver-operator characteristic curve
DENV: dengue virus
EWS: early warning signal
LA: local authority
MRSA: methicillin-resistant *Staphylococcus aureus*
ROC: receiver-operator characteristic
SARS-CoV: severe acute respiratory syndrome coronavirus
XDR TB: extensively drug-resistant tuberculosis
