# Supplementary material for: Dynamical footprints enable detection of disease emergence
Source: PLoS Biol. 2020 May 20;18(5):e3000697. doi: 10.1371/journal.pbio.3000697 (PMC7239390; doi:10.1371/journal.pbio.3000697)
Supplement: S1 Text — (PDF) [file pbio.3000697.s001.pdf]

# Supplemental text for "Dynamical footprints enable detection of disease emergence"

Tobias S Brett,[1,2*] Pejman Rohani,[1,2,3]

[1]Odum School of Ecology, University of Georgia, Athens, GA, USA,
[2]Center for the Ecology of Infectious Diseases, University of Georgia, Athens, GA, USA
[3]Department of Infectious Diseases, University of Georgia, Athens, GA, USA

*To whom correspondence should be addressed; E-mail: tsbrett@uga.edu.

## S1 Early-warning signals as a method to detect emergence

Early-warning signals (EWS) are model independent indicators of whether a dynamical system is approaching a critical point [1]. As the critical point is approached, the largest eigenvalue of the dynamical system, linearised about a stable equilibrium, approaches zero [2]. The eigenvalues govern the rate at which fluctuations away from the equilibrium decay. As the largest eigenvalue approaches zero, fluctuations in the associated eigendirection persist for increasingly long times. This is known as critical slowing down.

Mathematically, EWS are defined as the moments and correlation functions of the fluctuations away from the stable equilibrium [1]. As the critical point is approached the magnitude of the fluctuations increases and the time series becomes increasingly temporally correlated. These dynamical changes are reflected in the EWS, $\Theta_t$, which has led to suggestions that they may be a way of detecting imminent transitions[1].

The critical point for the dynamics of infectious disease transmission occurs at $R_0 = 1$. If $R_0 < 1$ the pathogen is incapable of sustained transmission and the disease-free equilibrium is stable. Whereas if $R_0 > 1$ the pathogen is capable of large scale sustained outbreaks and potential endemicity. Previous theoretical studies have also shown that EWS may potentially be used to anticipate the epidemic transition at $R_0 = 1$ [3, 4, 5].

Theoretically, EWS are defined as expectations of a stochastic process. In order to empirically calculate them from single time series data, estimators are constructed using moving window averages. In the previous literature a uniform window of fixed size was chosen [3, 4, 5]. Preliminary studies informing this paper found that an exponentially weighted moving average performs better, i.e. making the replacement,

$$E[f(X_t)] \approx Z^{-1} \sum_{s=t_0}^{t} e^{-\lambda(t-s)} f(X_s),$$
$$Z = \sum_{s=t_0}^{t} e^{-\lambda(t-s)}, \tag{1}$$

which we will use throughout. The decay rate is specified by the half-life $t_{1/2} = \ln(2)/\lambda$. The full list of EWS considered are listed in Table S1.

Each individual EWS can in principle be used in isolation to detect emergence. Alternatively, we here propose

that performance might be improved by considering a weighted sum of different EWS,

$$\begin{aligned} D_t(\boldsymbol{\Theta}_t, \boldsymbol{w}) &= g(B_t(\boldsymbol{\Theta}_t, \boldsymbol{w})), \\ B_t(\boldsymbol{\Theta}_t, \boldsymbol{w}) &= w_0 + \sum_{i=1}^{n} w_i \Theta_{i,t}, \end{aligned} \quad (2)$$

where the index $i = 1, \ldots, n$ labels the different EWS. We picked the logistic function,

$$g(B_t) = 1/(1 + e^{-B_t}), \quad (3)$$

to give a measure of epidemic risk ranging from 0 to 1. Emergence is detected when $D_t > c$, where $c$ is the detection threshold. In order for this approach to be viable various *a priori* unknown parameters need to be specified: i) the weights $\{w_i\}_{i=1}^{n}$ and intercept $w_0$ ii) the detection threshold $c$ and ii) the optimal half-life $t_{1/2}$. To do this we use a learning algorithm based approach.

Table S1: **List of early-warning signals.**

| EWS | Mathematical definition | Estimator | Theoretical prediction* |
|---|---|---|---|
| Mean | $\mu_t = E[X_t]$ | $\widehat{\mu}_t = \sum_{s=t_0}^{t} \frac{e^{-\lambda(t-s)} X_s}{Z}$ | $\frac{\zeta/\gamma}{1 - R_0}$ |
| Variance | $\sigma_t^2 = E[(X_t - \mu_t)^2]$ | $\widehat{\sigma}_t^2 = \sum_{s=t_0}^{t} \frac{e^{-\lambda(t-s)}(X_s - \widehat{\mu}_s)^2}{Z}$ | $\frac{\zeta/\gamma}{(1 - R_0)^2}$ |
| Coefficient of variation | $\text{CoV}_t = \sigma_t/\mu_t$ | $\widehat{\text{CoV}}_t = \frac{\widehat{\sigma}_t}{\widehat{\mu}_t}$ | $\sqrt{(\zeta/\gamma)}$ |
| Index of dispersion | $\text{IoD}_t = \sigma_t^2/\mu_t$ | $\widehat{\text{IoD}}_t = \frac{\widehat{\sigma}_t^2}{\widehat{\mu}_t}$ | $\frac{1}{1 - R_0}$ |
| Skewness | $\text{Skew}_t = \frac{E[(X_t - \mu_t)^3]}{\sigma_t^3}$ | $\widehat{\text{Skew}}_t = \frac{1}{\widehat{\sigma}_t^3} \sum_{s=t_0}^{t} \frac{e^{-\lambda(t-s)}(X_s - \widehat{\mu}_s)^3}{Z}$ | $\sqrt{(\zeta/\gamma)}(1 + R_0)$ |
| Kurtosis | $\text{Kurt}_t = \frac{E[(X_t - \mu_t)^4]}{\sigma_t^4}$ | $\widehat{\text{Kurt}}_t = \frac{1}{\widehat{\sigma}_t^4} \sum_{s=t_0}^{t} \frac{e^{-\lambda(t-s)}(X_s - \widehat{\mu}_s)^4}{Z}$ | $(\gamma/\zeta)(2 + R_0)^2$ $+3(1 - \gamma/\zeta)$ |
| Autocorrelation† | $\text{AC}_t(\delta) = \frac{E[(X_t - \mu_t)(X_{t-\delta} - \mu_{t-\delta})]}{\sigma_t \sigma_{t-\delta}}$ | $\widehat{\text{AC}}_t = \frac{1}{\widehat{\sigma}_t \widehat{\sigma}_{t-\delta}} \sum_{s=t_0}^{t} \Big[ e^{-\lambda(t-s)}(X_s - \widehat{\mu}_s) \times (X_{s-\delta} - \widehat{\mu}_{s-\delta})/Z \Big]$ | $e^{-(1-R_0)\gamma\delta}$ |

*All BDI theory results calculated using the stationary approximation, assuming $R_0(t) = \epsilon t$ and $\epsilon\delta \ll 1$, see [4].
†$\delta$ denotes one time step.

## S2 Learning algorithm

### S2.1 Transfer learning approach

We want to find a measure of emergence risk which is robust to uncertainty in epidemiological parameters. To do this we fit the logistic model $D_t$ to a set of 10,000 simulated time series, half emerging and half not, using logistic regression. The simulated time series are generated using the canonical Susceptible-Exposed-Infectious-Recovered (SEIR) model, with uncertain parameters drawn using latin hypercube sampling (LHS).

### S2.2 Simulation algorithm

Simulations of epidemiological dynamics were performed using a stochastic SEIR compartmental model [6]. The model incorporates demographic stochasticity, stochasticity in the transmission rate, and reporting error. Simulated data were generated using the Gibson-Bruck next reaction method [7], which simulates a sequence of transition events (e.g. infection, recovery, birth and death), and returns the number of individuals in each model compartment

through time. We assumed the mean population size $N_0$ is stable, and that the *per capita* birth and death rates are both $\nu = 1/75$ years$^{-1}$. Latent and infectious periods were exponentially distributed. We used a parameterisation appropriate for mumps, with mean latent period $1/\sigma = 13$ days and infectious period $1/\gamma = 6$ days [8]. Infection is imported to the population from external sources at rate $\zeta$. Infectious individuals spread the infection to others with transmission rate $\beta(t)$. Table S2 lists the SEIR model parameters, and Table S3 lists the rates and effects of the transitions in the model. The reproductive number of the SEIR model is

$$R_0(t) = \frac{\sigma\beta(t)}{(\gamma+\nu)(\sigma+\nu)}. \tag{4}$$

The parameters $\sigma$, $\gamma$ and $\nu$ are all constant, changes in $R_0(t)$ reflect changes in the pathogen's transmissibility. We therefore parameterised $\beta(t)$ in terms of the other parameters via Eq. 4.

Simulations were initialised at the disease-free equilibrium $S = N_0$ at time $t = -10$ years with initial reproduction number $R_0^{(i)}$. After a 10 year equilibration period, $R_0(t)$ followed a stochastic trajectory of duration $T = 10$ years starting from $R(0) = R_0^{(i)}$. For emergence, $R_0(t)$ was a realisation of a Brownian bridge ending at the emergence threshold $R_0(T) = 1$,

$$R_0(t) = R_0^{(i)} + (1 - R_0^{(i)}) \left[ W(t) + \left(\frac{t}{T}\right)^{\kappa} \right] - \frac{tW(T)}{T}, \tag{5}$$

where $W(t)$ is a Wiener process with $W(0) = 0$ and variance $\sigma_{bb}^2 t$. For time in units of days, we set $\sigma_{bb}^2 = 10^{-5}$. The parameter $\kappa$ controls the curvature of the path. For $0 < \kappa < 1$ the path is concave, for $\kappa = 1$ the path is linear, for $\kappa > 1$ the path is convex (see Fig. S1). Both initial value $R_0^{(i)}$ and curvature $\kappa$ are selected by LHS.

For the non-emerging scenario $R_0(t)$ was a realisation of an Ornstein-Uhlenbeck process with fixed mean, solving the stochastic differential equation

$$\frac{dR_0(t)}{dt} = -k(R_0(t) - R_0^{(i)}) + \xi(t), \tag{6}$$

where the white noise term is defined as $\xi(t) = \frac{dW(t)}{dt}$ with $E[\xi(t)] = 0$ and $E[\xi(t)\xi(t')] = \sigma_{ou}^2\delta(t-t')$. We fix $k = 0.01$ days$^{-1}$and $\sigma_{ou}^2 = \sigma_{bb}^2 = 10^{-5}$. The initial value $R_0^{(i)}$ was drawn from the LHS with the same range as for the emerging scenario, ensuring that both sets of time series (emerging and non-emerging) are initially statistically indistinguishable.

At the end of each aggregation period (set to 1 week, to match the mumps dataset), reporting error was applied to the case count by sampling a negative binomial distribution,

$$P(K_t = k|C_t) = \frac{\Gamma(\phi+k)}{k!\Gamma(\phi)} \left(\frac{\rho C_t}{\rho C_t + \phi}\right)^k \left(\frac{\phi}{\rho C_t + \phi}\right)^{\phi}, \tag{7}$$

with reporting probability $\rho$ and dispersion parameter $\phi$ [9, 10]. Given $C_t$ cases, the mean number reported is $\mu_t = \rho C_t$. The variance is specified by the dispersion parameter via the relation $\sigma_t^2 = \mu_t + \mu_t^2/\phi$. Increasing $\phi$ reduces the overdispersion of the data, so that for large $\phi$ the distribution of reports is approximately Poisson. We set $\phi = 100$, and selected $\rho$ by LHS.

The five parameters with uncertainty – $N_0$, $\rho$, $\zeta$ , $R_0^{(i)}$ and $\kappa$ – were selected for each realisation of the model by LHS with ranges listed in Table S4. The latin hypercube generated $M = 10000$ samples in $(0, 1)^5$. For $\rho$ and $R_0^{(i)}$ we linearly transformed the samples to get the appropriate distribution. To get a wide range of population sizes we assumed $\ln N_0$ has a uniform distribution over the parameter range. For similar reasons we chose that $1/\zeta$ is uniformly distributed. To ensure an equal balance between convex and concave functions for $R_0(t)$, we chose that $1/\kappa$ is uniformly distributed for $\kappa < 1$ and $\kappa$ is uniformly distributed for $\kappa \geq 1$ with equal probability of $\kappa < 1$ and $\kappa > 1$. Half of the samples were then assigned to be non-emerging, and the other half were emerging (see above).

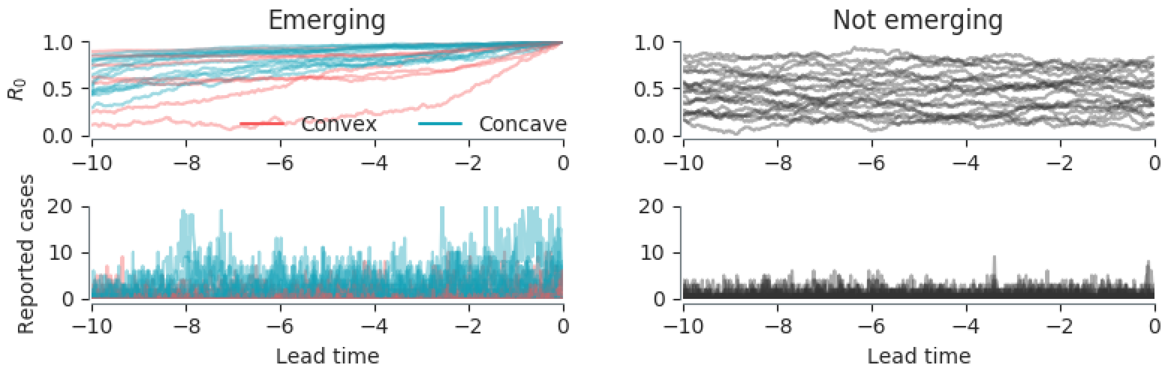


Figure S1: Time series of 20 emerging (left column) and non-emerging (right column) samples from the latin hypercube. Top row shows trajectories of $R_0(t)$, bottom row shows case reports through time. Convex trajectories are generated using $\kappa > 1$, whereas concave use $\kappa < 1$.

Table S2: Model symbols

| Symbol | Definition |
|---|---|
| $S$ | number of susceptible individuals |
| $E$ | number of exposed individuals |
| $I$ | number of infectious individuals |
| $R$ | number of removed individuals |
| $C$ | number of cases |
| $\zeta$ | importation rate |
| $\beta(t)$ | transmission rate |
| $\sigma$ | exposed to infectious rate |
| $\gamma$ | recovery rate |
| $\nu$ | per capita birth and death rate |
| $N_0$ | average population size |

Table S3: Transitions of the SEIR transmission model. At the beginning of each aggregation period $C$ is reset to 0.

| Name | $(\Delta S, \Delta E, \Delta I, \Delta R, \Delta C)$ | Propensity |
|---|---|---|
| birth of $S$ | $(1, 0, 0, 0, 0)$ | $\nu N_0$ |
| death of $S$ | $(-1, 0, 0, 0, 0)$ | $\nu S$ |
| death of $E$ | $(0, -1, 0, 0, 0)$ | $\nu E$ |
| death of $I$ | $(0, 0, -1, 0, 0)$ | $\nu I$ |
| death of $R$ | $(0, 0, 0, -1, 0)$ | $\nu R$ |
| importation | $(-1, 1, 0, 0, 0)$ | $\zeta S/N_0$ |
| transmission | $(-1, 1, 0, 0, 0)$ | $\beta(t) SI/N_0$ |
| $E$ to $I$ | $(0, -1, 1, 0, 0)$ | $\sigma E$ |
| recovery | $(0, 0, -1, 1, 1)$ | $\gamma I$ |

### S2.3 Lasso regression

In order to prevent over-fitting to the training data, we found values for the weights $\{w_i\}_{i=1}^{n}$ and intercept $w_0$ by minimising the negative log-likelihood of the logistic model over the space of weights subject to an $l^1$-penalisation

Table S4: Latin hypercube space

| Parameter | Range | Transformation |
|---|---|---|
| $N_0$ | $[5 \times 10^4, 5 \times 10^6]$ | logarithm |
| $\rho$ | [0.01,0.5] | linear |
| $\zeta$ | [1/30,1] | inverse |
| $R_0^{(i)}$ | [0.1,0.9] | linear |
| $\kappa$ | [1/5,5] | inverse for $< 1$, none for $> 1$ |

with strength $p$. This is also known as lasso regression [11]. The complete cost function we minimised is

$$C(\boldsymbol{w}, p) = -\frac{1}{MT} \sum_{j=1}^{M} \sum_{t=1}^{T} \left\{ y^{(j)} \ln D_t^{(j)}(\boldsymbol{\Theta}_t,^{(j)} \boldsymbol{w}) + (1 - y^{(j)}) \ln(1 - D_t^{(j)}(\boldsymbol{\Theta}_t^{(j)}, \boldsymbol{w})) \right\} + p \sum_{i=1}^{n} |w_i|, \quad (8)$$

where the superscript $j$ labels the replicate and $y^{(j)} = 1$ if the sample was emerging and 0 if not. We performed the lasso regression using the python package sci-kit learn [12], which uses the liblinear solver [13].

### S2.4 AUC test statistic

After fitting, $D_t(\boldsymbol{\Theta}_t, \boldsymbol{w})$ provides a measure of emergence risk. A data point is labelled as emerging if $D_t$ exceeds the detection threshold.

In order to determine the optimum threshold, we calculated the Receiver Operating Characteristic (ROC) curve, a parametric plot of the true positive rate against the false positive rate as a function of the detection threshold. The Area Under the ROC Curve (AUC) statistic provides a measure of how distinct the two different classes are when using a classifier [10]. For example, an AUC $= 1$ means that $D_t$ is always larger for emerging time series than for non-emerging time series. There therefore exists a range of intermediary threshold values for which the classifier performs perfectly. Likewise, AUC $= 0$ means $D_t$ is always smaller for emerging time series than non-emerging time series, and the classifier also performs perfectly. Performance is worst if AUC $= 0.5$, as then it is equiprobable that an observed $D_t$ is emerging or not-emerging. AUC values further from 0.5 therefore imply better performance.

We calculate: (i) the AUC for the full ten years of data using the overall true and false positive rates and (ii) the AUC through time, i.e. each time point individually, using the true and false positive rates for that 4-weekly period. The detection threshold $c$ is chosen to minimise the sum of type I and type II errors for the full dataset.

### S2.5 Hyperparameter selection

The optimum set of weights and detection threshold depend on two hyperparameters: the half-life $t_{1/2}$ of the moving window average and the penalty strength $p$ in the lasso regression. These were selected by 10-fold cross-validation [11]. The time series were randomly split into 10 chunks, for each chunk the other 9 chunks are used for training with the remaining chunk used as an out-of-fit test. For each out-of-fit-test we calculated the AUC test statistic, we then calculated the mean $\mu_{\text{AUC}}$ and the standard deviation $\sigma_{\text{AUC}}$. We performed a grid search over pairs of $t_{1/2}$ and $p$ to find

$$(t_{1/2}^m, p^m) = \underset{(t_{1/2}, p)}{\text{argmax}}\ \mu_{\text{AUC}}(t_{1/2}, p). \quad (9)$$

This parameter combination corresponds to over-fitting the training data [11]. The best hyperparameter combination instead corresponds to the maximum penalty strength (to reduce over-fitting) and the minimum half-life (to improve responsiveness of $D_t$ to changes) that provide satisfactory performance. As criteria for satisfactory

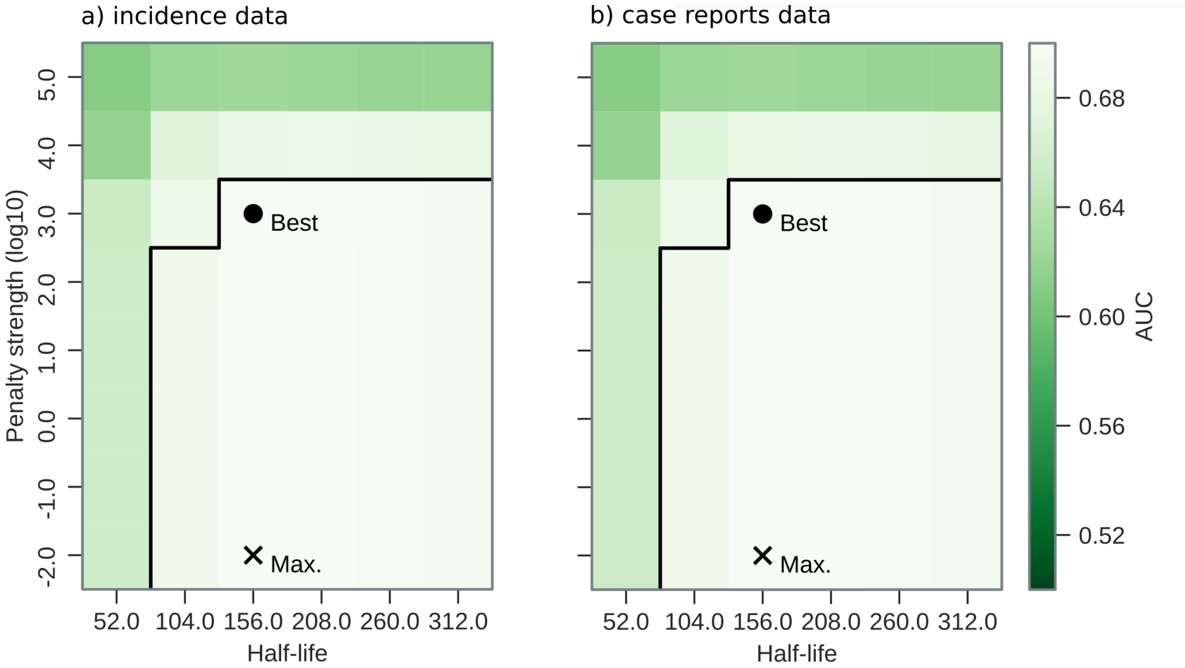


Figure S2: Heat maps showing mean AUC values from cross validation for a) incidence data b) case reports data. The results are largely unaffected by data type, with the same maximum, $\mu_{\text{AUC}} = 0.68$, located at $t^m_{1/2} = 156$ weeks, $p^m = 10^{-3}$. The black contour indicates the region within one standard deviation of the maximum. The best hyperparameter values are the same for both data types, $t_{1/2} = 156$ and $p = 10^4$.

performance we required that the mean AUC falls within one standard deviation of the maximum mean AUC [11], i.e.

$$\mu_{\text{AUC}}(t_{1/2}, p) + \sigma_{\text{AUC}}(t_{1/2}, p) > \mu_{\text{AUC}}(t^m_{1/2}, p^m). \tag{10}$$

We first found the value for $p$,

$$p^{best} = \max_{(p, t_{1/2})} p, \tag{11}$$

subject to the constraint in Eq. 10. The best half-life was then found,

$$t^{\text{best}}_{1/2} = \min_{t_{1/2}} t_{1/2}, \tag{12}$$

subject to the same constraint, but with $p$ fixed to $p^{\text{best}}$. Note that the resulting $(t^{\text{best}}_{1/2}, p^{\text{best}})$ depends on the order the maximisation and minimisation are performed. We determined in preliminary investigations that maximising $p$ was more important to performance than minimising $t_{1/2}$, hence it is performed first. Results from the cross-validation are shown in Fig. S2.

The resulting pair of hyperparameters, $(t^{\text{best}}_{1/2}, p^{\text{best}})$, were then used to i) calculate the EWS and ii) perform lasso regression on the full dataset to give a final set of weights and intercept. The weights, intercept and half-life can be applied to calculate EWS and $D_t$ from epidemiological data without further fitting. Fitted values for the weights and hyperparameters are listed in Table S5.

Table S5: **List of EWS weights and intercepts. Weights found by performing lasso regression on the dataset indicated in the columns with hyper parameters $t_{1/2}^{\mathbf{best}} = 156$ weeks and $p^{\mathbf{best}} = 0.001$ (see Fig. S2).**

| EWS | Dataset<br>Simulated case reports | Simulated incidence | Pertussis | Pertussis† |
|---|---|---|---|---|
| | EWS weight | | | |
| Mean | 0.0 | 9486 | 0.0 | 0.0 |
| Variance | 0.008 | 0.0 | 0.0 | 0.0 |
| Coefficient of variation | −0.661 | −0.639 | −0.659 | −0.651 |
| Index of dispersion | −0.123 | −4308 | −0.002 | 0.0 |
| Skewness | 1.527 | 1.511 | 0.846 | 0.851 |
| Kurtosis | −0.120 | −0.123 | −0.047 | −0.048 |
| Autocorrelation (lag 1) | 0.762 | 0.685 | −1.317 | 0.0 |
| Autocorrelation (lag 2) | 1.105 | 1.055 | 1.626 | 0.0 |
| | Intercept | | | |
| | −1.163 | −1.235 | 0.804 | 0.645 |

†Fit only included the three most important EWS (skewness, kurtosis and coefficient of variation).

### S2.6 Summary of the EWS-based detection method

In summary, our approach to detect disease emergence is as follows:

1. Each of the 8 EWS (the autocorrelation, variance etc) is a non-linear transformation of the input time series data that theory predicts to vary as the transition is approached.
2. We use a logistic model to transform the 8 EWS into one measure of emergence risk. This choice of transformation is chosen for pragmatic reasons, as it allows us to fit the weights using logistic regression.
3. To find the optimal weight to assign each EWS we fit the logistic model to a training data set using lasso regression. The training data were generated using an SEIR transmission model over a range of parameters to ensure robustness to parametric uncertainty. Because we are interested in robustness, we dont need to be as concerned with whether the model is a good model of the dynamics.

Our approach is therefore a statistical approach (a logistic model), using a set of non-linear data transforms as predictors, with the aim of classifying emerging and non-emerging time series.

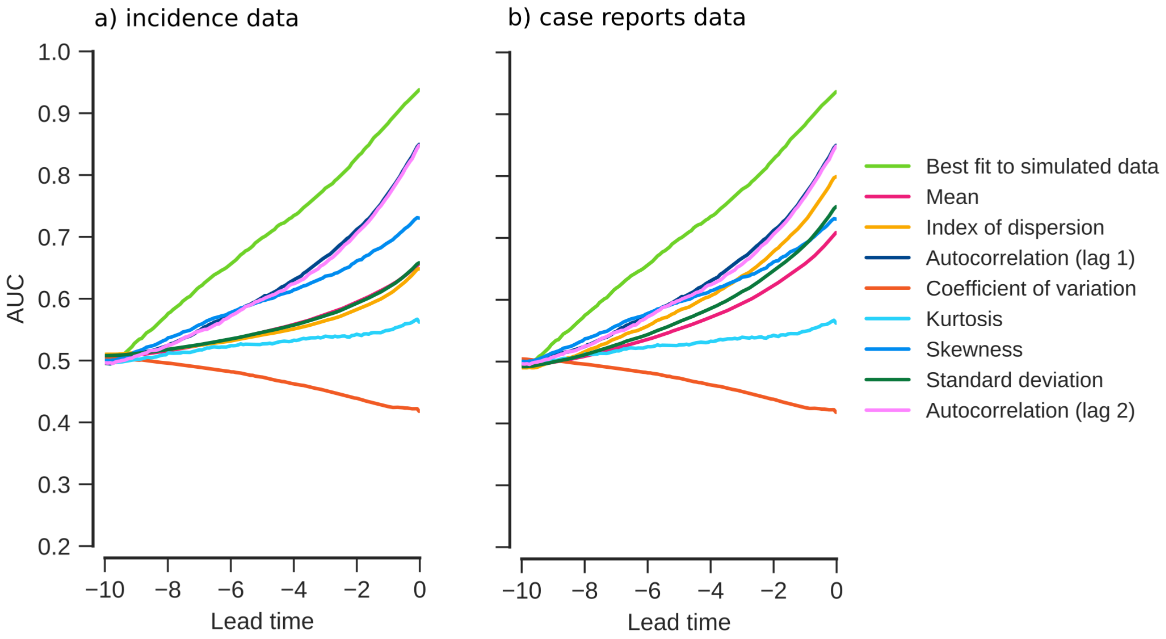


Figure S3: AUC through time for the complete simulated dataset. In panel a) Case counts are converted to incidence data before EWS are calculated, in panel b) raw case counts are used. Our measure of emergence risk, $D_t$, with weights fitted to the simulated data (light green line) outperforms any individual EWS at distinguishing between emerging and non-emerging time series. Performance is only affected by data type for the mean, variance and index of dispersion.

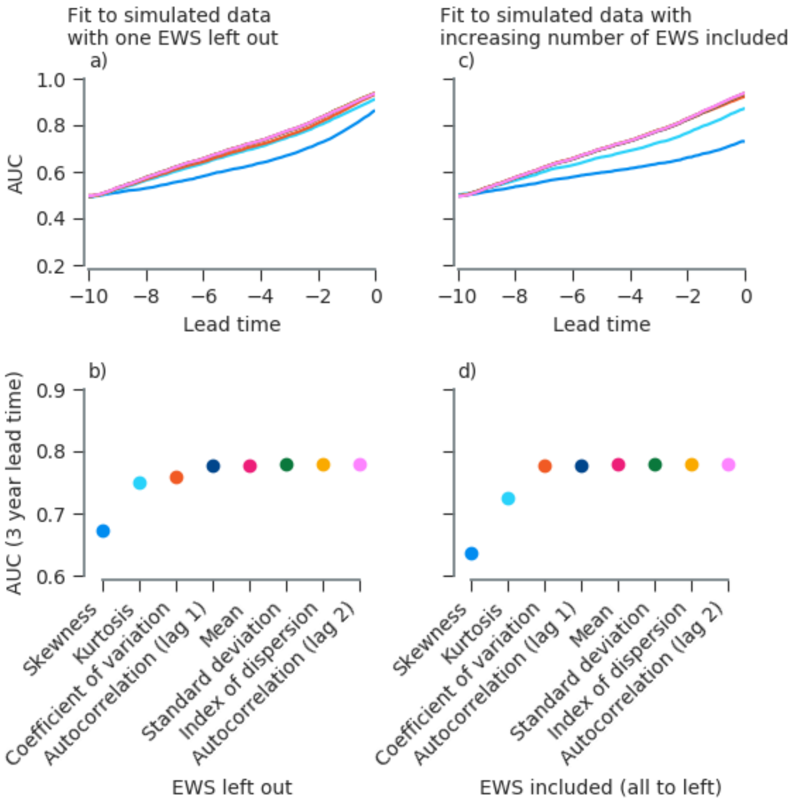


Figure S4: Analysis of importance of individual EWS to performance of $D_t$. a) AUC through time when fitting $D_t$ with one EWS left out. Colour matches Fig. S3, and indicates the EWS left out. b) Same data as panel a), but showing only the AUC at a lead time of 3 years. EWS are ordered from left to right based on impact on performance. Exclusion of the skewness is seen the have the most detrimental impact on performance, followed by the kurtosis and coefficient of variation. c-d) Based on the ranking in b) EWS are sequentially added with colour indicating the rightmost EWS included in the fit. Including the skewness, kurtosis and coefficient of variation is sufficient to get close-to-optimal performance. Calculated using incidence data.

### S2.7 Impact of emergence mechanism and the presence of additional time-varying parameters on algorithm performance

We examined whether performance of our algorithm was sensitive to (i) the mechanism underlying (re-)emergence and (ii) the presence of time-varying parameters (in addition to $R_{\text{eff}}$).

Specifically, if (re-)emergence is driven by waning of vaccinal or natural immunity [14], then increases in $R_{\text{eff}}$ would be concave ($d^2R_{\text{eff}}/dt^2 < 0$). In contrast, if (re-)emergence is the result of the evolution of immune evasion following sequential mutation [15, 16], then the trajectory of $R_{\text{eff}}$ through time would be convex ($d^2R_{\text{eff}}/dt^2 > 0$) [17].

The primary dataset we used for training assumed $R_0(t)$ is the only time-varying parameter. To test the robustness of the learning algorithm fit we also trained on three additional simulated datasets: i) a dataset with only convex ($\kappa > 1$) trajectories for $R_0$ and ii) a dataset with only concave ($\kappa \leq 1$) trajectories for $R_0$ iii) a dataset where $N_0$, $\rho$ and $\zeta$ all varied linearly in time, with start and end points included separately in the latin hypercube with identical ranges given in Table S4.

We retrained the learning algorithm on simulated datasets consisting of only (i) concave and (ii) convex trends. Surprisingly, the EWS weights obtained by fitting to simulated data that comprised both mechanisms of increase performed best compared to training on concave/convex data alone (Figs. S5 & S6). We interpret this as support for our claim that $D_t$ is a mechanism-independent measure of emergence risk. We performed a similar comparison using data simulated from a model with multiple time-varying parameters, in addition to $R_0$ (which drives the transition), to assess their confounding effects (Fig. S7). We again found that the EWS weights obtained by fitting to simulated data without such covariates performed comparably to the optimal fit with covariates.

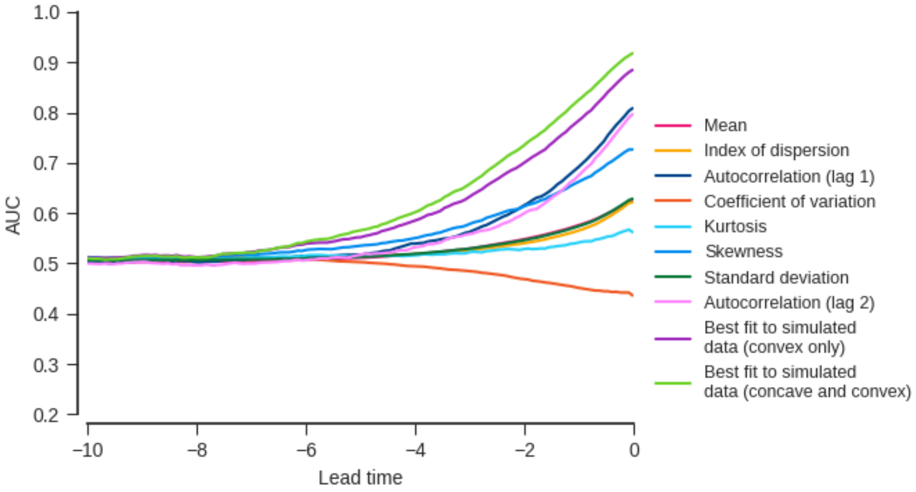


Figure S5: AUC through time for the simulated dataset with only convex trends in $R_0$. Case counts are converted to incidence data before EWS are calculated. The fit to the simulated dataset with both concave and convex trends (light green) has comparable performance to the fit to just convex simulated data (dark purple).

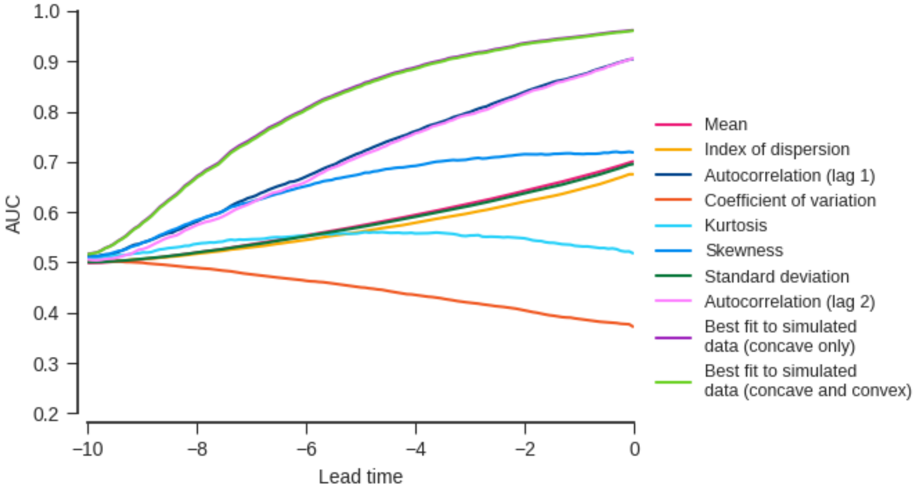


Figure S6: AUC through time for the simulated dataset with only concave trends in $R_0$. Case counts are converted to incidence data before EWS are calculated. The fit to the simulated dataset with both concave and convex trends (light green) has comparable performance to the fit to just concave simulated data (dark purple).

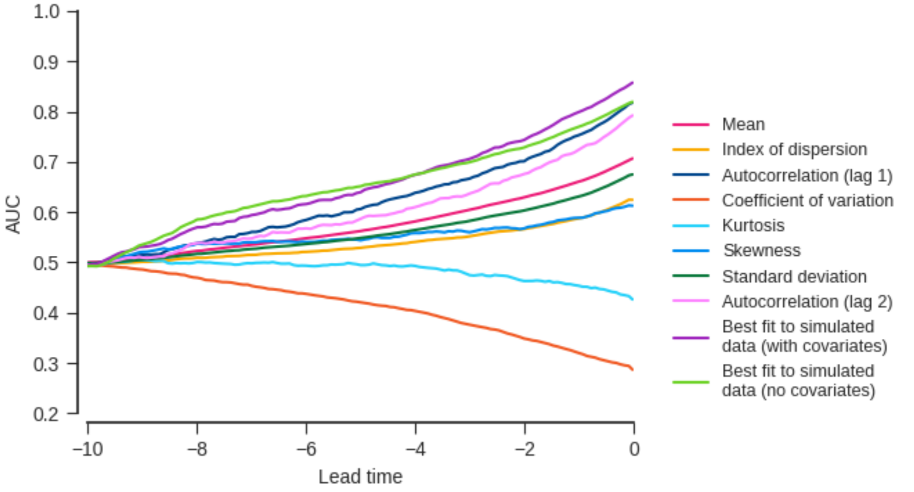


Figure S7: AUC through time for the simulated dataset with the inclusion of covariates. Covariates included are: the population size, the reporting probability and the importation rate. Covariates vary linearly through time, initial and final values are included as independent variables in the latin hypercube with identical ranges given in Table S4. Case counts are converted to incidence data before EWS are calculated. The fit to the simulated dataset with covariates (light green) has comparable performance to the fit without their inclusion (dark purple).

## S3 Motivation for using the General Mixture Model

We chose to use a General Mixture Model (GMM) to model the distribution of 2004-2005 mumps outbreak sizes. This model is motivated by basic epidemiological theory and by observations of patterns in the data.

From the epidemiological theory, the threshold $R_{\text{eff}} = 1$ divides the outcome of the introduction of an infectious individual into two phases:

- If $R_{\text{eff}} < 1$ the outbreak is small and the size is determined by stochasticity in the transmission dynamics, with larger outbreaks being increasingly rare (see for example [18]).
- Conversely, if $R_{\text{eff}} > 1$, the outbreak size is much larger and is an approximately deterministic function of the localitys epidemiological properties (e.g. the initial susceptible population size). In this case, most of the variability in outbreak size stems from heterogeneity in the epidemiological parameters.

We therefore modelled the outbreak size distribution with a GMM that was composed of two exponential distributions, one for small outbreaks and one (with a heavier tail) for large outbreaks. We chose exponential distributions to minimise the number of parameters estimated in the GMM, and due to the lack of obvious peaks in the outbreak size distribution.

# References


[1] Scheffer M, Bascompte J, Brock WA, Brovkin V, Carpenter SR, Dakos V, et al. Early-warning signals for critical transitions. Nature. 2009;461(7260):53–59.

[2] Strogatz S. Nonlinear dynamics and chaos: with applications to physics, biology, chemistry and engineering. Perseus Books Group; 2001.

[3] O'Regan SM, Drake JM. Theory of early warning signals of disease emergence and leading indicators of elimination. Theoretical Ecology. 2013;6(3):333–357.

[4] Brett TS, Drake JM, Rohani P. Anticipating the emergence of infectious diseases. Journal of The Royal Society Interface. 2017;14(132):20170115.

[5] O'Dea EB, Park AW, Drake JM. Estimating the distance to an epidemic threshold. Journal of The Royal Society Interface. 2018;15(143):20180034.

[6] Keeling MJ, Rohani P. Modeling Infectious Diseases in Humans and Animals. Princeton University Press; 2008.

[7] Gibson MA, Bruck J. Efficient exact stochastic simulation of chemical systems with many species and many channels. The Journal of Physical Chemistry A. 2000;104(9):1876–1889.

[8] Anderson RM, May RM. Infectious Diseases of Humans: Dynamics and Control. Oxford university press; 1992.

[9] Bretó C, He D, Ionides EL, King AA. Time series analysis via mechanistic models. The Annals of Applied Statistics. 2009;3(1):319–348.

[10] Brett TS, O'Dea EB, Marty É, Miller PB, Park AW, Drake JM, et al. Anticipating epidemic transitions with imperfect data. PLoS Computational Biology. 2018;14(6):e1006204.

[11] Friedman J, Hastie T, Tibshirani R. The Elements of Statistical Learning. Springer series in statistics New York, NY, USA:; 2001.

[12] Pedregosa F, Varoquaux G, Gramfort A, Michel V, Thirion B, Grisel O, et al. Scikit-learn: Machine Learning in Python. Journal of Machine Learning Research. 2011;12:2825–2830.

[13] Fan RE, Chang KW, Hsieh CJ, Wang XR, Lin CJ. LIBLINEAR: A library for large linear classification. Journal of Machine Learning Research. 2008;9(Aug):1871–1874.

[14] Domenech de Cellès M, Magpantay FMG, King AA, Rohani P. The impact of past vaccination coverage and immunity on pertussis resurgence. Science Translational Medicine. 2018;10(434).

[15] Gandon S, Mackinnon MJ, Nee S, Read AF. Imperfect vaccines and the evolution of pathogen virulence. Nature. 2001;414(6865):751.

[16] Antia R, Regoes RR, Koella JC, Bergstrom CT. The role of evolution in the emergence of infectious diseases. Nature. 2003;426(6967):658.

[17] Arinaminpathy N, McLean A. Evolution and emergence of novel human infections. Proceedings of the Royal Society of London B: Biological Sciences. 2009; p. rspb20091059.

[18] Blumberg S, Lloyd-Smith JO. Inference of $R_0$ and transmission heterogeneity from the size distribution of stuttering chains. PLoS Comput Biol. 2013;9(5):e1002993.
