# Supplementary material for: Dynamical footprints enable detection of disease emergence
Source: PLoS Biol. 2020 May 20;18(5):e3000697. doi: 10.1371/journal.pbio.3000697 (PMC7239390; doi:10.1371/journal.pbio.3000697)
Supplement: S1 Table — (DOCX) [file pbio.3000697.s002.docx]

*All BDI theory results calculated using the stationary approximation, assuming $R_{0}(t)=\epsilon t$ and $\epsilon\delta\ll1$, see [1].

| **S1 Table List of early-warning signals** | | | |
| --- | --- | --- | --- |
| EWS | Mathematical definition | Estimator | Theoretical prediction* |
| Mean | $\mu_{t}=E[X_{t}]$ | ${\overset{̂}{\mu}}_{t}=\overset{t}{\underset{s=t_{0}}{\sum}}\frac{e^{-\lambda(t-s)}X_{s}}{Z}$ | $\frac{\zeta/\gamma}{1-R_{0}}$ |
| Variance | $\sigma_{t}^{2}=E[(X_{t}-\mu_{t})^{2}]$ | $\sigma_{t}^{2}=\overset{t}{\underset{s=t_{0}}{\sum}}\frac{e^{-\lambda(t-s)}(X_{s}-{\overset{̂}{\mu}}_{s})^{2}}{Z}$ | $\frac{\zeta/\gamma}{(1-R_{0})^{2}}$ |
| Coefficient  of variation | ${CoV}_{t}=\sigma_{t}/\mu_{t}$ | ${\overset{̂}{CoV}}_{t}=\frac{{\overset{̂}{\sigma}}_{t}}{\mu_{t}}$ | $\sqrt{(\zeta/\gamma)}$ |
| Index of  dispersion | ${IoD}_{t}=\sigma_{t}^{2}/\mu_{t}$ | ${\overset{̂}{IoD}}_{t}=\frac{\sigma_{t}^{2}}{\mu_{t}}$ | $\frac{1}{1-R_{0}}$ |
| Skewness | ${Skew}_{t}=\frac{E[(X_{t}-\mu_{t})^{3}]}{\sigma_{t}^{3}}$ | ${\overset{̂}{Skew}}_{t}=\frac{1}{\sigma_{t}^{3}}\overset{t}{\underset{s=t_{0}}{\sum}}\frac{e^{-\lambda(t-s)}(X_{s}-{\overset{̂}{\mu}}_{s})^{3}}{Z}$ | $\sqrt{(\zeta/\gamma)}(1+R_{0})$ |
| Kurtosis | ${Kurt}_{t}=\frac{E[(X_{t}-\mu_{t})^{4}]}{\sigma_{t}^{4}}$ | ${\overset{̂}{Kurt}}_{t}=\frac{1}{\sigma_{t}^{4}}\overset{t}{\underset{s=t_{0}}{\sum}}\frac{e^{-\lambda(t-s)}(X_{s}-{\overset{̂}{\mu}}_{s})^{4}}{Z}$ | $(\gamma/\zeta)(2+R_{0})^{2}+3(1-\gamma/\zeta)$ |
| Autocorrelation^†^ | ${AC}_{t}(\delta)=\frac{E[(X_{t}-\mu_{t})(X_{t-\delta}-\mu_{t-\delta})]}{\sigma_{t}\sigma_{t-\delta}}$ | ${\overset{̂}{AC}}_{t}=\frac{1}{\sigma_{t}\sigma_{t-\delta}}\overset{t}{\underset{s=t_{0}}{\sum}}\frac{e^{-\lambda(t-s)}(X_{s}-{\overset{̂}{\mu}}_{s})(X_{s-\delta}-{\overset{̂}{\mu}}_{s-\delta})}{Z}$ | $e^{-(1-R_{0})\gamma\delta}$ |

^†^denotes one time step.

1. T. S. Brett, J. M. Drake, P. Rohani, *Journal of The Royal Society Interface* **14**, 20170115 (2017).
