# Supplementary material for: Dynamical footprints enable detection of disease emergence
Source: PLoS Biol. 2020 May 20;18(5):e3000697. doi: 10.1371/journal.pbio.3000697 (PMC7239390; doi:10.1371/journal.pbio.3000697)
Supplement: S2 Table — (DOCX) [file pbio.3000697.s003.docx]

| **S2 Table Model symbols** | |
| --- | --- |
| Symbol | Definition |
| $S$ | Number of susceptible individuals |
| $E$ | Number of exposed individuals |
| $I$ | Number of infectious individuals |
| $R$ | Number of removed individuals |
| $C$ | Number of cases |
| $\zeta$ | Importation rate |
| $\beta(t)$ | Transmission rate |
| $\sigma$ | Exposed to infectious rate |
| $\gamma$ | Recovery rate |
| $\nu$ | *per capita* birth and death rate |
| $N_{0}$ | Average population size |

*At the beginning of each aggregation period $C$ is reset to zero.
