# Supplementary material for: Dynamical footprints enable detection of disease emergence
Source: PLoS Biol. 2020 May 20;18(5):e3000697. doi: 10.1371/journal.pbio.3000697 (PMC7239390; doi:10.1371/journal.pbio.3000697)
Supplement: S3 Table — At the beginning of each aggregation period C is reset to 0. (DOCX) [file pbio.3000697.s004.docx]

| **S3 Table Transitions of the SEIR transmission model.** | | | | | | |
| --- | --- | --- | --- | --- | --- | --- |
|  | Transition effects | | | | |  |
| Transition name | $\boldsymbol{\Delta}\boldsymbol{S}$ | $\boldsymbol{\Delta}\boldsymbol{E}$ | $\boldsymbol{\Delta}\boldsymbol{I}$ | $\boldsymbol{\Delta}\boldsymbol{R}$ | $\boldsymbol{\Delta}\boldsymbol{C}$* | Propensity |
| birth of $\boldsymbol{S}$ | 1 | 0 | 0 | 0 | 0 | $\nu N_{0}$ |
| death of $\boldsymbol{S}$ | -1 | 0 | 0 | 0 | 0 | $\nu S$ |
| death of $\boldsymbol{E}$ | 0 | -1 | 0 | 0 | 0 | $\nu E$ |
| death of $I$ | 0 | 0 | -1 | 0 | 0 | $\nu I$ |
| death of $\boldsymbol{R}$ | 0 | 0 | 0 | -1 | 0 | $\nu R$ |
| importation | -1 | 1 | 0 | 0 | 0 | $\zeta S/N_{0}$ |
| transmission | -1 | 1 | 0 | 0 | 0 | $\beta(t)SI/N_{0}$ |
| $\boldsymbol{E}$ to $I$ | 0 | -1 | 1 | 0 | 0 | $\sigma E$ |
| recovery | 0 | 0 | -1 | 1 | 1 | $\gamma I$ |
