# Supplementary material for: Dynamical footprints enable detection of disease emergence
Source: PLoS Biol. 2020 May 20;18(5):e3000697. doi: 10.1371/journal.pbio.3000697 (PMC7239390; doi:10.1371/journal.pbio.3000697)
Supplement: S4 Table — (DOCX) [file pbio.3000697.s005.docx]

| **S4 Table Latin hypercube space** | | |
| --- | --- | --- |
| Parameter | Range | Transformation |
| $N_{0}$ | [5×10^4^, 5×10^6^] | logarithm |
| $\rho$ | [0.01, 0.5] | linear |
| $\zeta$ | [1/30, 1] | inverse |
| $R_{0}^{(i)}$ | [0.1, 0.9] | linear |
| $\kappa$ | [1/5, 5] | inverse for < 1; none for > 1 |
