# Supplementary material for: Dynamical footprints enable detection of disease emergence
Source: PLoS Biol. 2020 May 20;18(5):e3000697. doi: 10.1371/journal.pbio.3000697 (PMC7239390; doi:10.1371/journal.pbio.3000697)
Supplement: S5 Table — Weights found by performing lasso regression on the dataset indicated in the columns with hyperparameters weeks (see S2 Fig). EWS, early warning signal. (DOCX) [file pbio.3000697.s006.docx]

| **S5 Table List of EWS weights and intercepts** | | | | |
| --- | --- | --- | --- | --- |
|  | Dataset | | | |
| EWS | Simulated case reports | Simulated incidence | Pertussis | Pertussis* |
| Mean | 0.0 | 9486 | 0.0 | 0.0 |
| Variance | 0.008 | 0.0 | 0.0 | 0.0 |
| Coefficient of variation | -0.661 | -0.639 | -0.659 | -0.651 |
| Index of dispersion | -0.123 | -4308 | -0.002 | 0.0 |
| Skewness | 1.527 | 1.511 | 0.846 | 0.851 |
| Kurtosis | -0.120 | -0.123 | -0.047 | -0.048 |
| Autocorrelation (lag 1) | 0.762 | 0.685 | -1.317 | 0.0 |
| Autocorrelation (lag 2) | 1.105 | 1.055 | 1.626 | 0.0 |
|  | **Intercept** | | | |
|  | -1.163 | -1.235 | 0.804 | 0.645 |

*Fit only included the three most important EWS (skewness, kurtosis and coefficient of variation).

Weights found by performing lasso regression on the dataset indicated in the columns with hyper parameters $t_{1/2}^{\text{best}}=156$ weeks and $p^{\text{best}}=0.001$ (see S2 Fig).
