# Supplementary figures and images for: Dynamical footprints enable detection of disease emergence
Source: PLoS Biol. 2020 May 20;18(5):e3000697. doi: 10.1371/journal.pbio.3000697 (PMC7239390; doi:10.1371/journal.pbio.3000697)

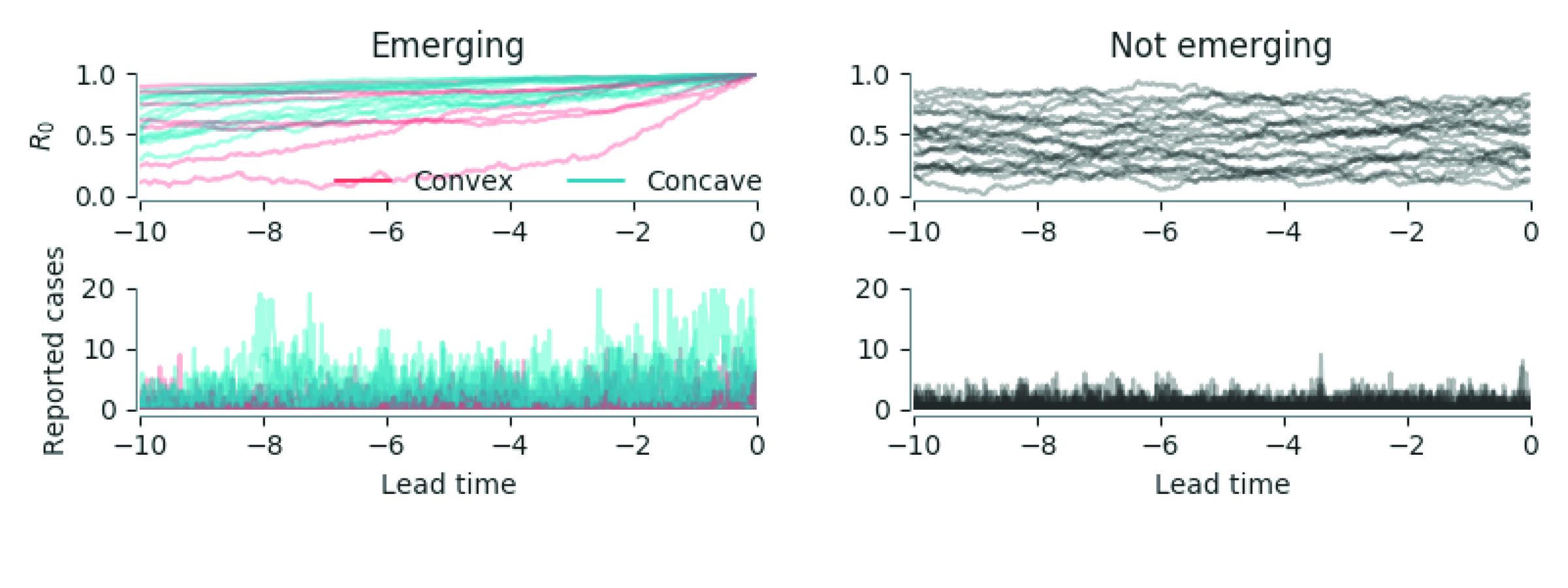

Supplement: S1 Fig — Top row shows trajectories of R0(t); bottom row shows case reports through time. Convex trajectories are generated using κ > 1, whereas concave use κ < 1. Data and code used to generate this figure can be found at https://doi.org/10.5281/zenodo.3713381. (TIF) [file pbio.3000697.s007.tif]

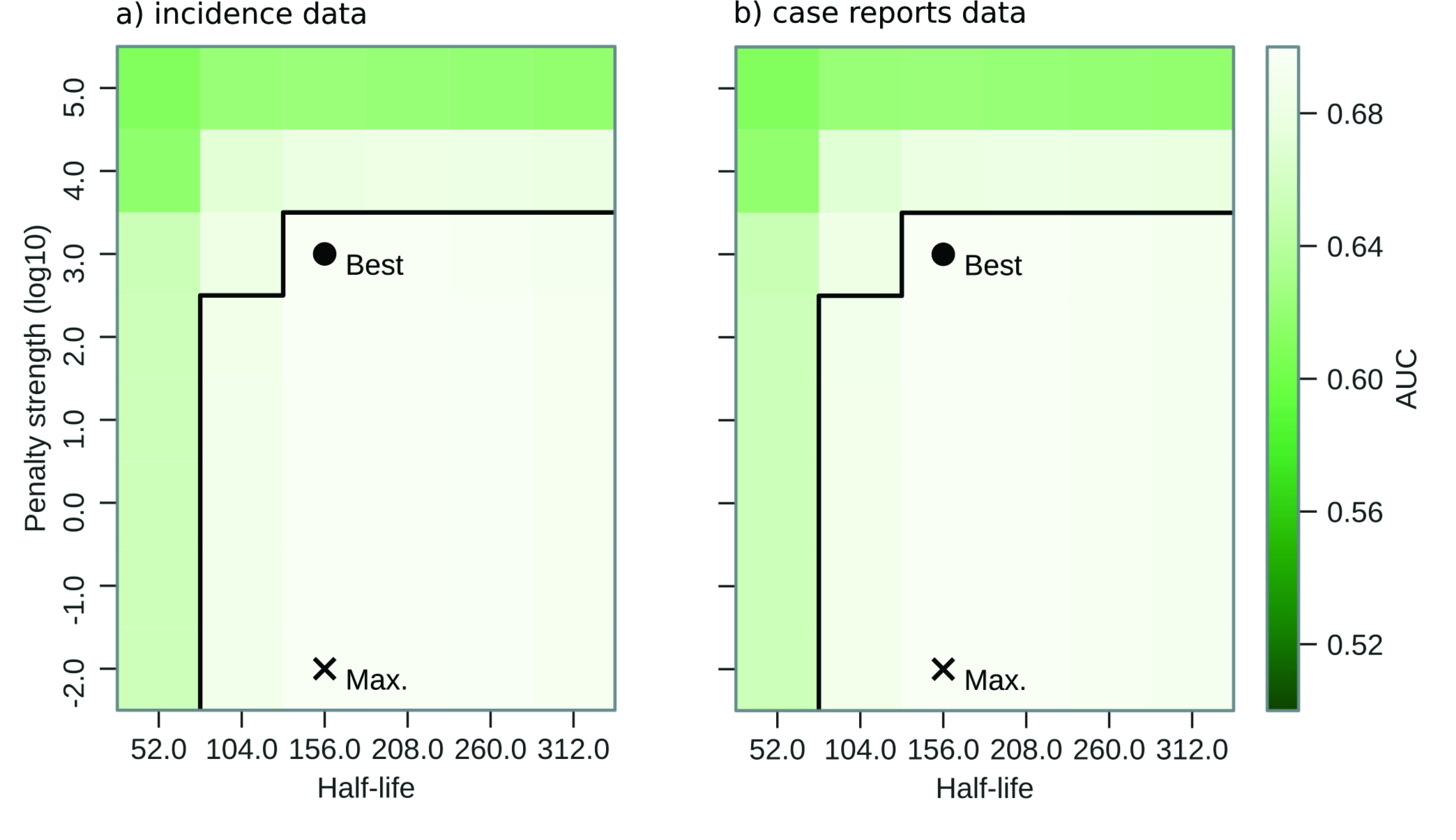

Supplement: S2 Fig — The results are largely unaffected by data type, with the same maximum, μAUC = 0.68, located at t1/2m=156 weeks, pm = 10−3. The black contour indicates the region within 1 standard deviation of the maximum. The best hyperparameter values are the same for both data types, t1/2 = 156 and p = 104. Data and code used to generate this figure can be found at https://doi.org/10.5281/zenodo.3713381. AUC, area under the receiver-operator characteristic curve. (TIF) [file pbio.3000697.s008.tif]

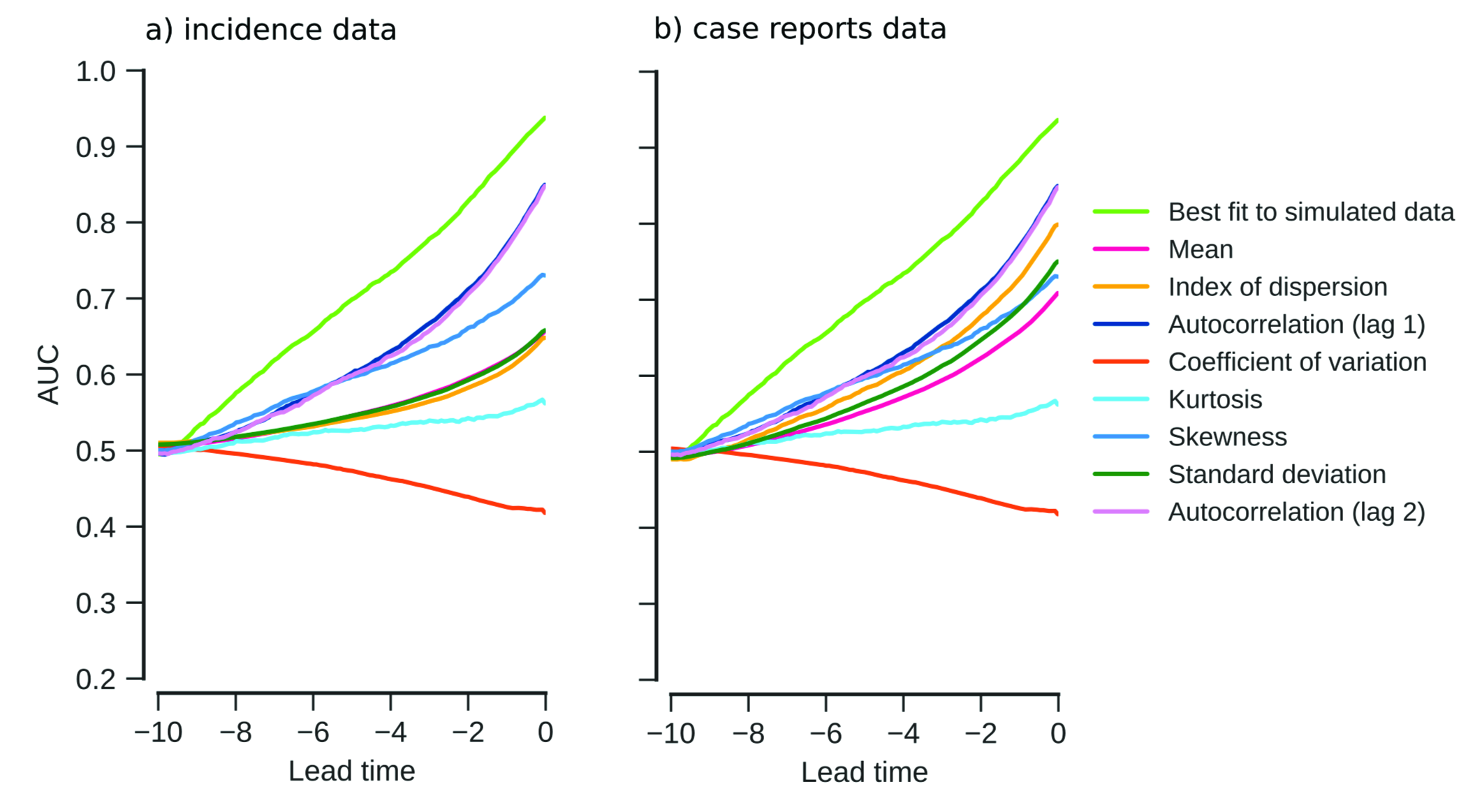

Supplement: S3 Fig — In panel (a), case counts are converted to incidence data before EWSs are calculated; in panel (b), raw case counts are used. Our measure of emergence risk, Dt, with weights fitted to the simulated data (light green line) outperforms any individual EWS at distinguishing between emerging and nonemerging time series. Performance is only affected by data type for the mean, variance, and index of dispersion. Data and code used to generate this figure can be found at https://doi.org/10.5281/zenodo.3713381. AUC, area under the receiver-operator characteristic curve; EWS, early warning signal. (TIF) [file pbio.3000697.s009.tif]

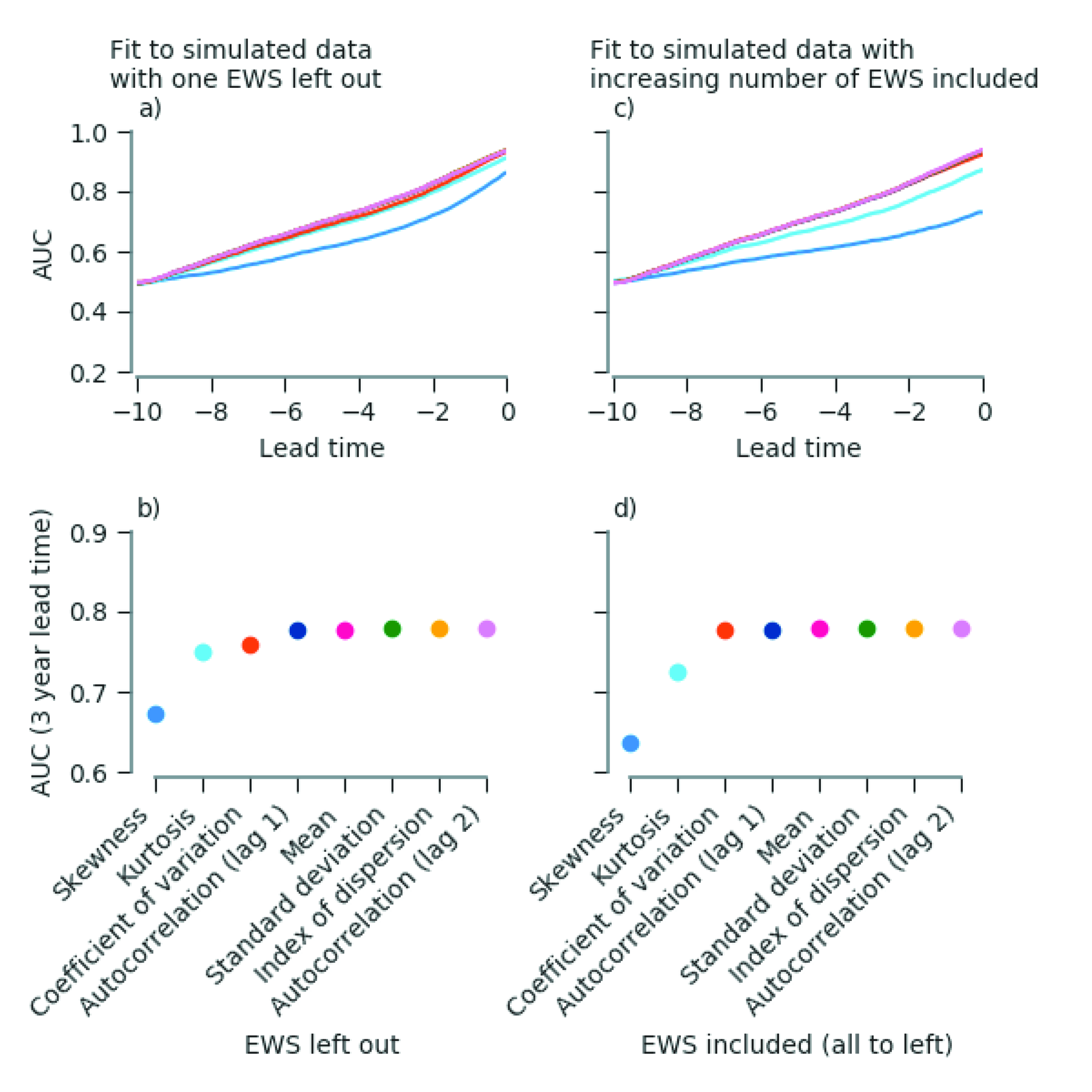

Supplement: S4 Fig — (a) AUC through time when fitting Dt with 1 EWS left out. Color matches S3 Fig and indicates the EWSs left out. (b) Same data as panel (a) but showing only the AUC at a lead time of 3 years. EWSs are ordered from left to right based on impact on performance. Exclusion of the skewness is seen to have the most detrimental impact on performance, followed by the kurtosis and coefficient of variation. (c,d) Based on the ranking in (b), EWSs are sequentially added, with color indicating the rightmost EWS included in the fit. Including the skewness, kurtosis, and coefficient of variation is sufficient to get close to optimal performance. Calculated using incidence data. Data and code used to generate this figure can be found at https://doi.org/10.5281/zenodo.3713381. AUC, area under the receiver-operator characteristic curve; EWS, early warning signal. (TIF) [file pbio.3000697.s010.tif]

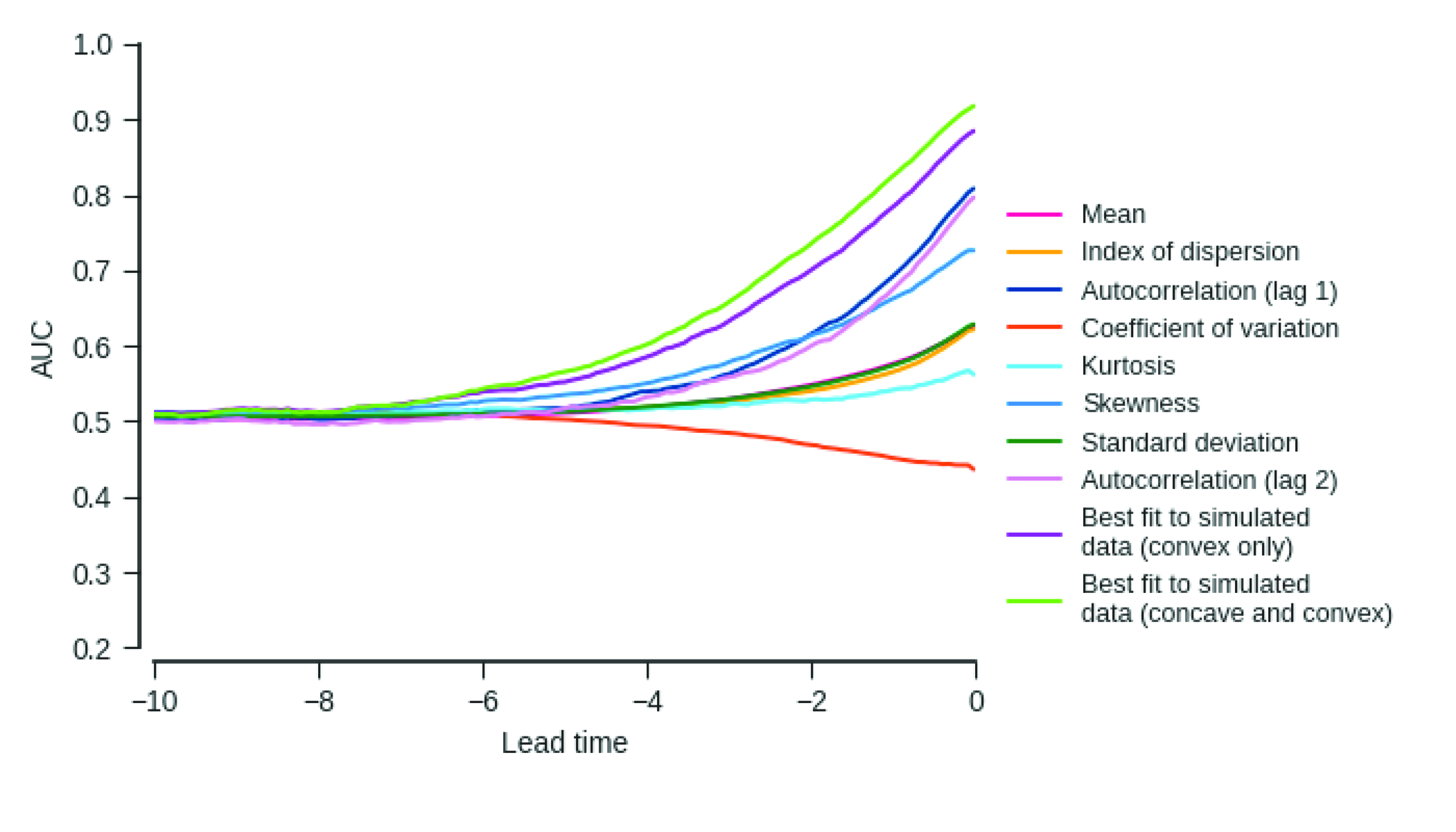

Supplement: S5 Fig — Case counts are converted to incidence data before EWSs are calculated. The fit to the simulated dataset with both concave and convex trends (light green) has comparable performance to the fit to just convex simulated data (dark purple). Data and code used to generate this figure can be found at https://doi.org/10.5281/zenodo.3713381. AUC, area under the receiver-operator characteristic curve; EWS, early warning signal. (TIF) [file pbio.3000697.s011.tif]

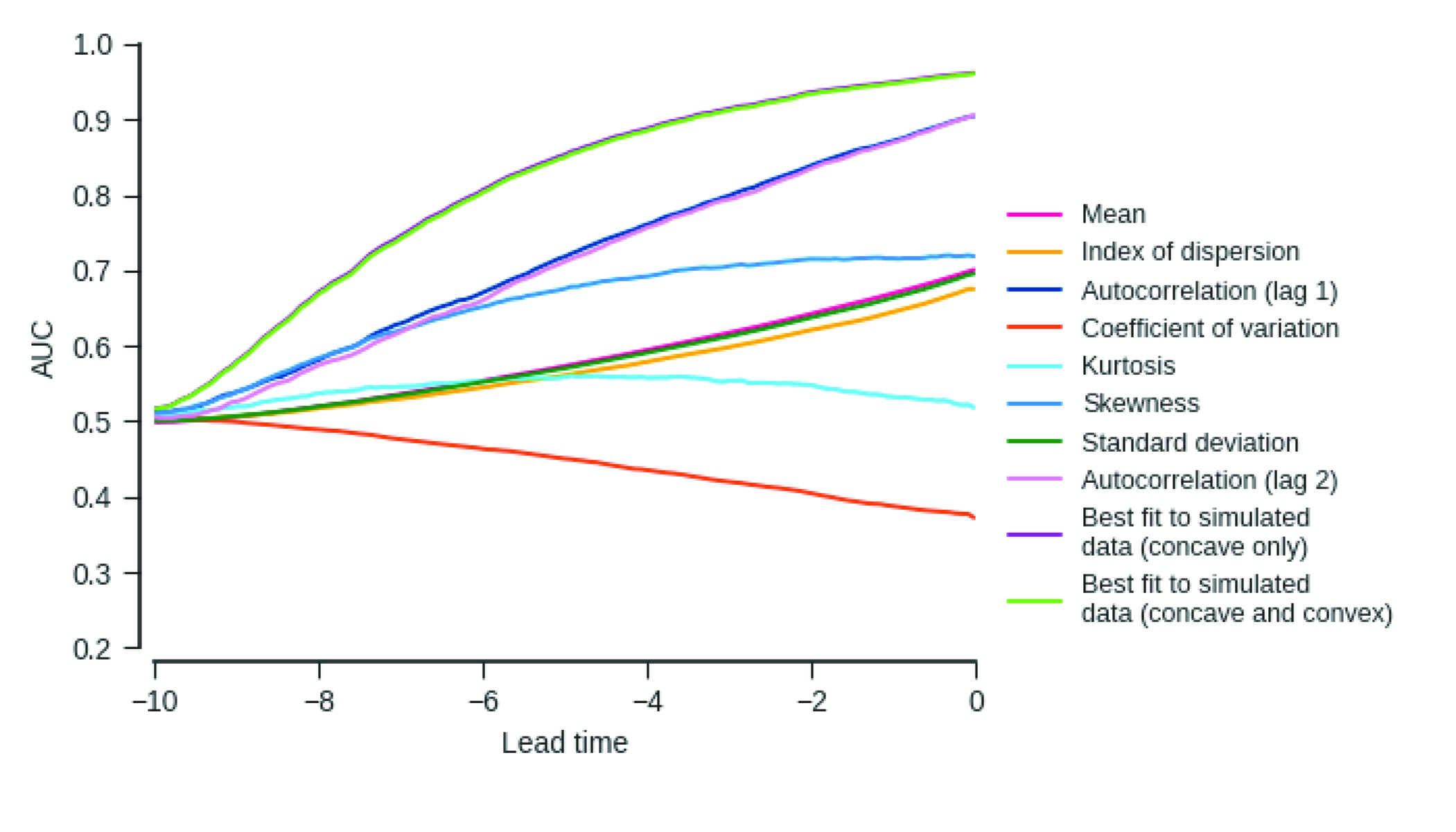

Supplement: S6 Fig — Case counts are converted to incidence data before EWSs are calculated. The fit to the simulated dataset with both concave and convex trends (light green) has comparable performance to the fit to just concave simulated data (dark purple). Data and code used to generate this figure can be found at https://doi.org/10.5281/zenodo.3713381. AUC, area under the receiver-operator characteristic curve; EWS, early warning signal. (TIF) [file pbio.3000697.s012.tif]

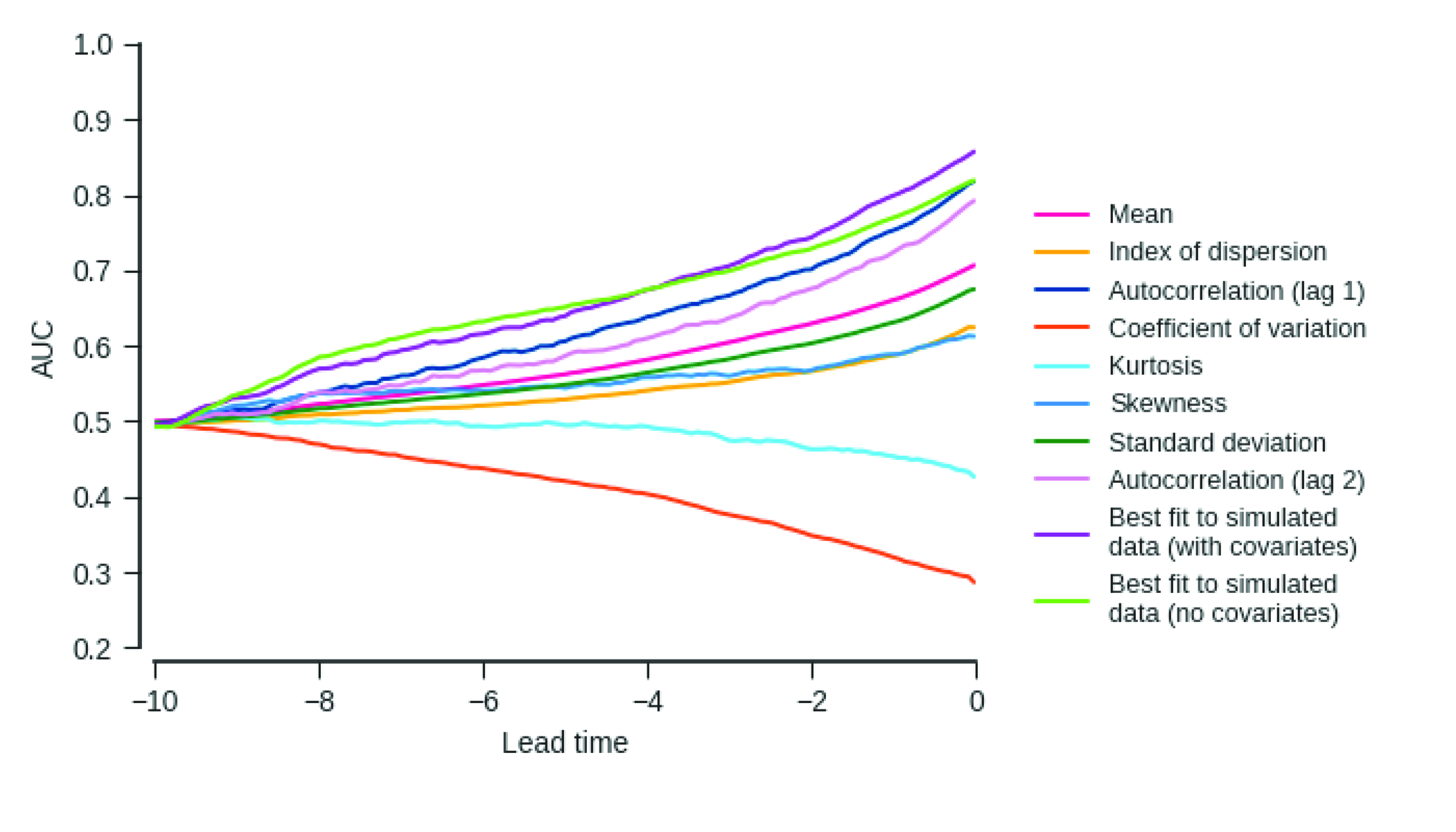

Supplement: S7 Fig — Covariates included are the population size, the reporting probability, and the importation rate. Covariates vary linearly through time; initial and final values are included as independent variables in the Latin hypercube with identical ranges given in S4 Table. Case counts are converted to incidence data before EWSs are calculated. The fit to the simulated dataset with covariates (light green) has comparable performance to the fit without their inclusion (dark purple). Data and code used to generate this figure can be found at https://doi.org/10.5281/zenodo.3713381. AUC, area under the receiver-operator characteristic curve; EWS, early warning signal. (TIF) [file pbio.3000697.s013.tif]

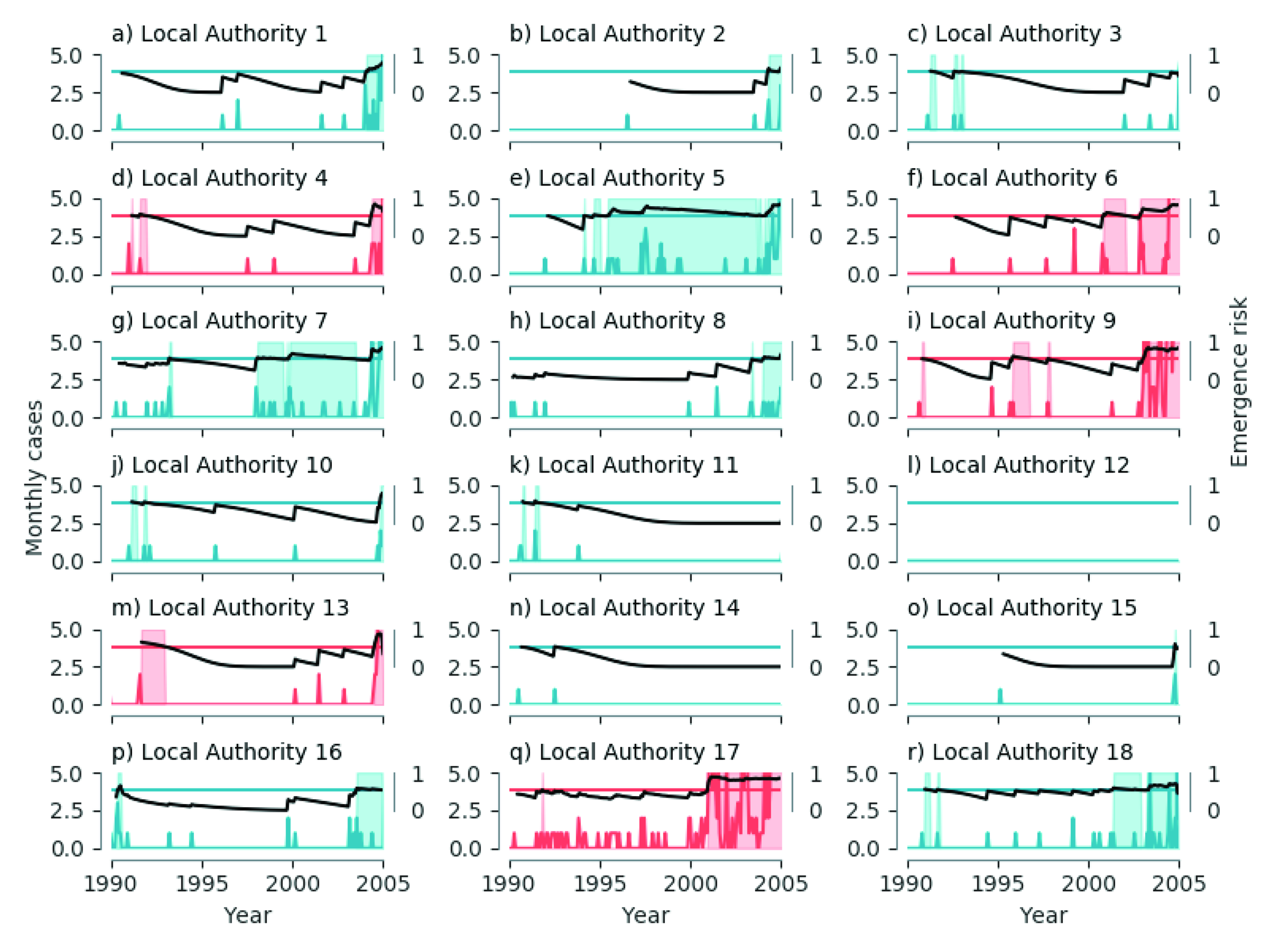

Supplement: S8 Fig — The coloring indicates the classification using the GMM, with red and blue corresponding to large and small outbreaks, respectively. Shaded backgrounds indicate Dt < c. Data and code used to generate this figure can be found at https://doi.org/10.5281/zenodo.3713381. GMM, general mixture model. (TIF) [file pbio.3000697.s014.tif]

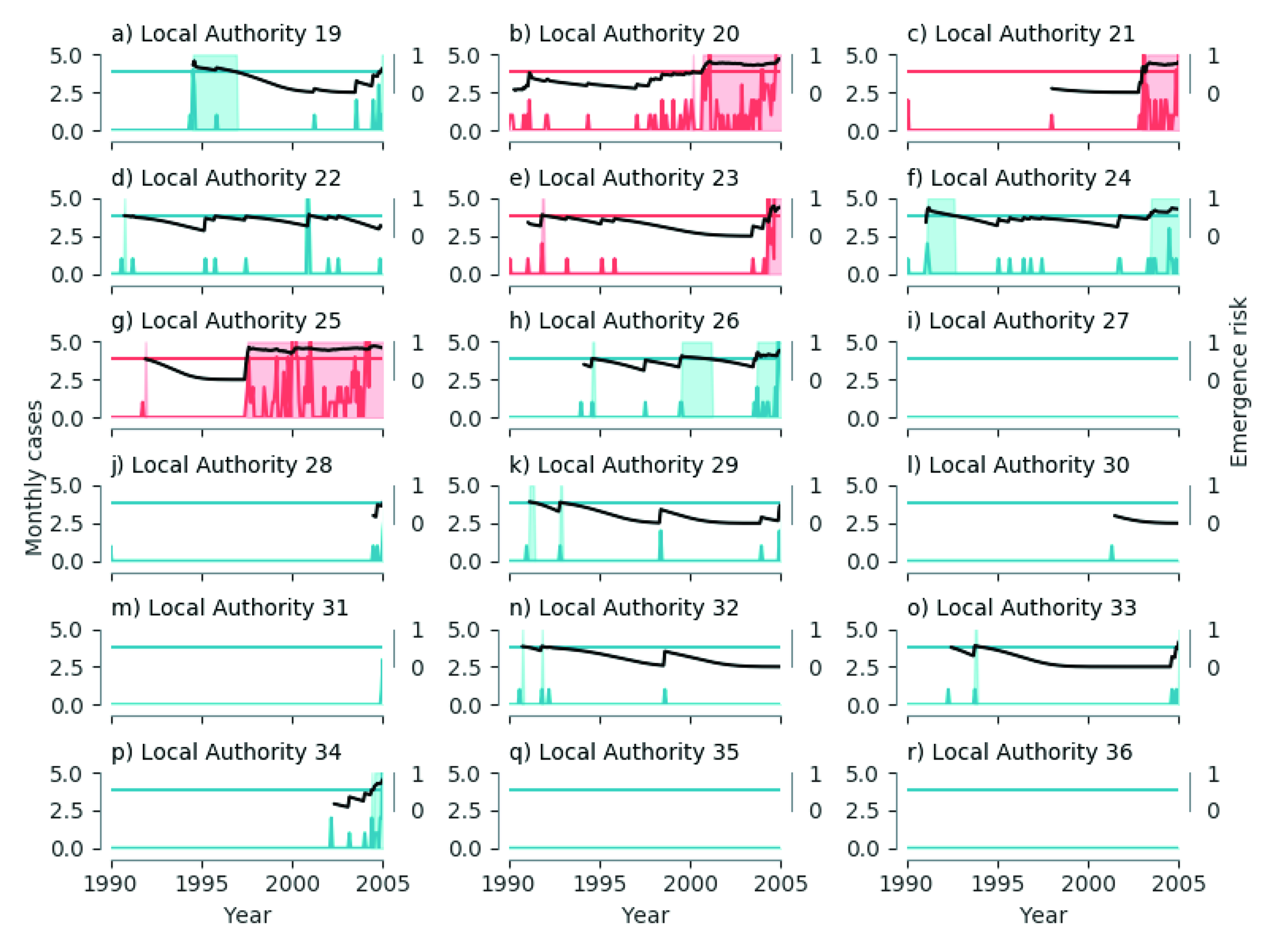

Supplement: S9 Fig — (TIF) [file pbio.3000697.s015.tif]

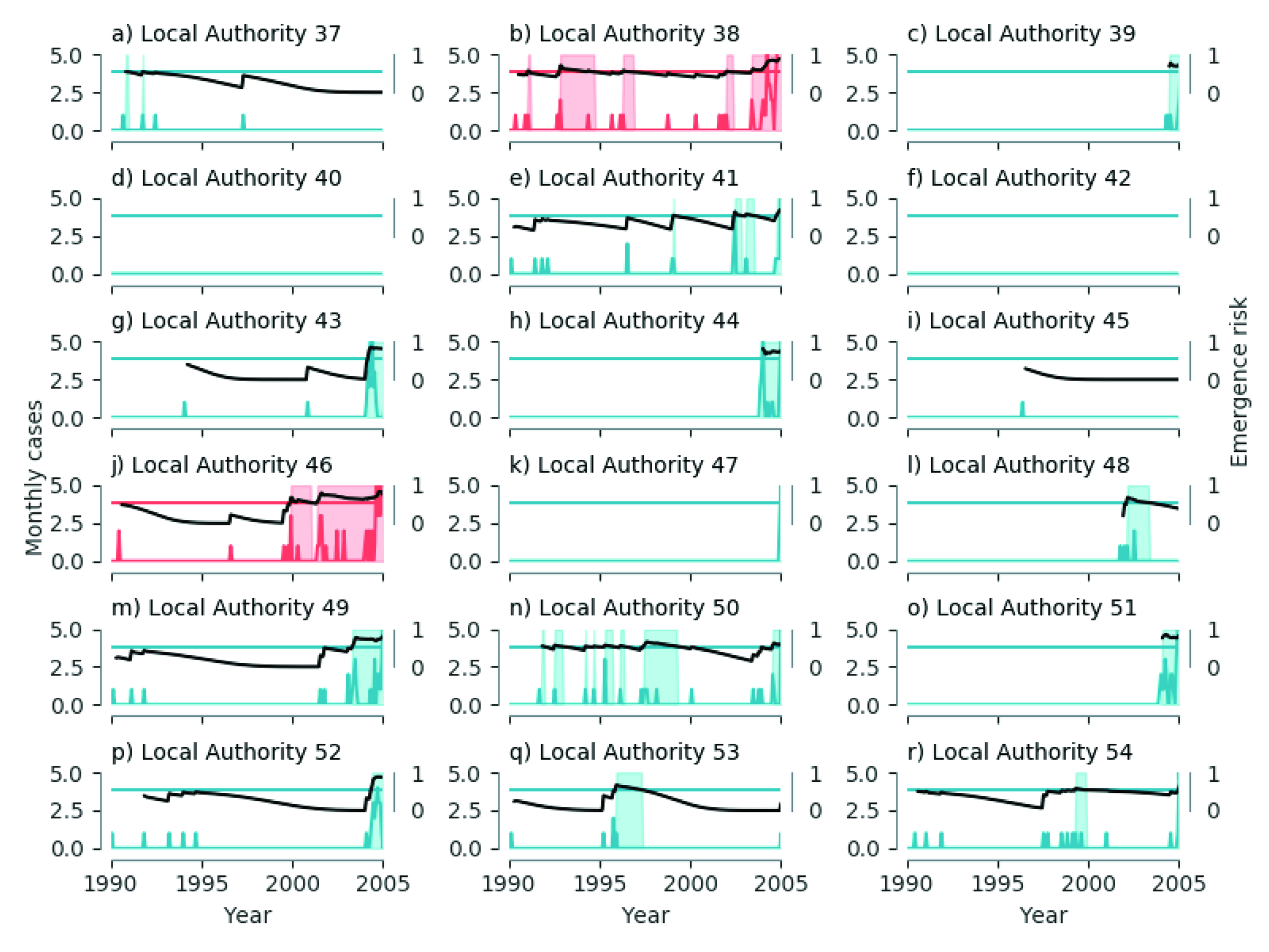

Supplement: S10 Fig — (TIF) [file pbio.3000697.s016.tif]

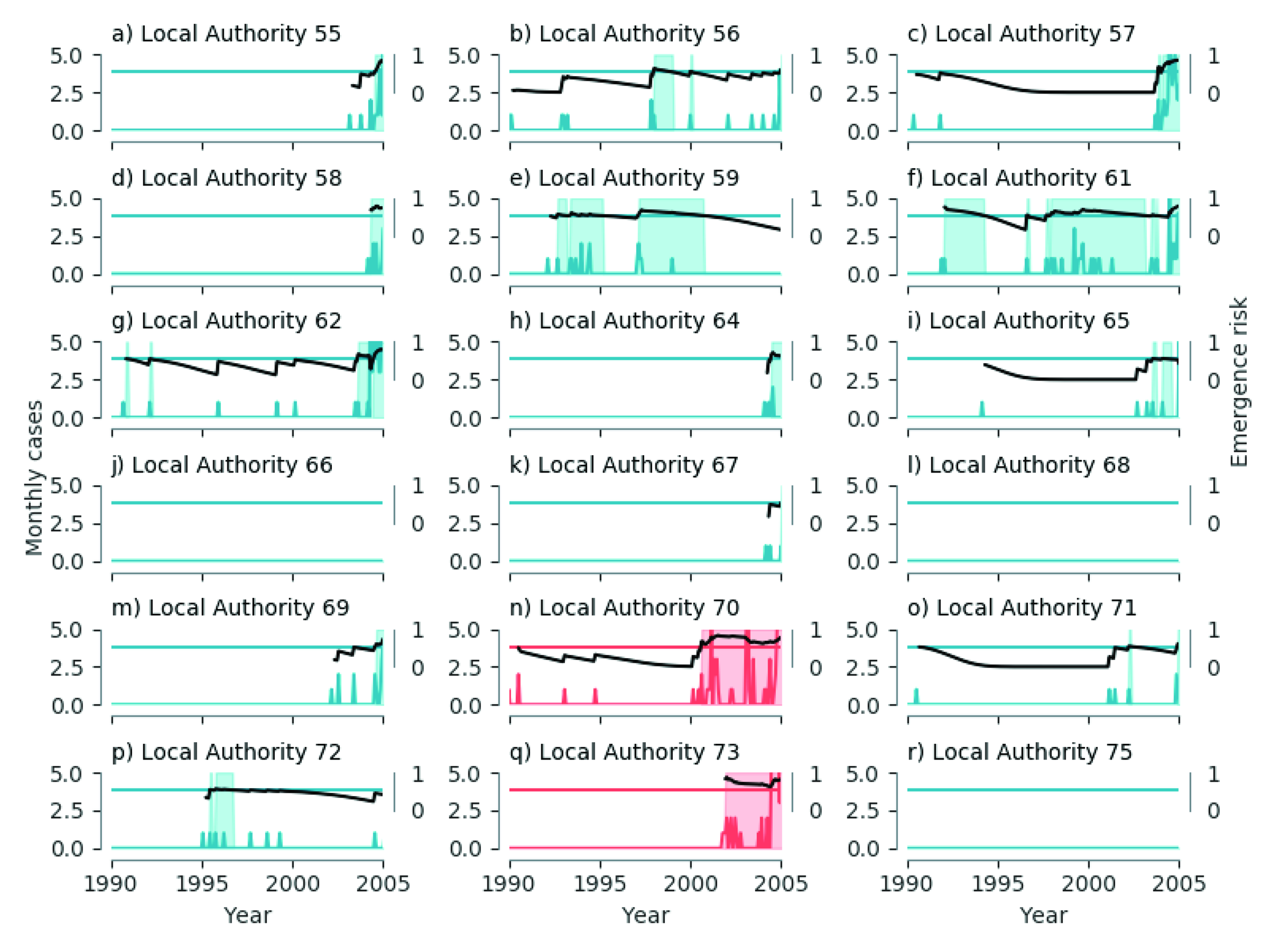

Supplement: S11 Fig — (TIF) [file pbio.3000697.s017.tif]

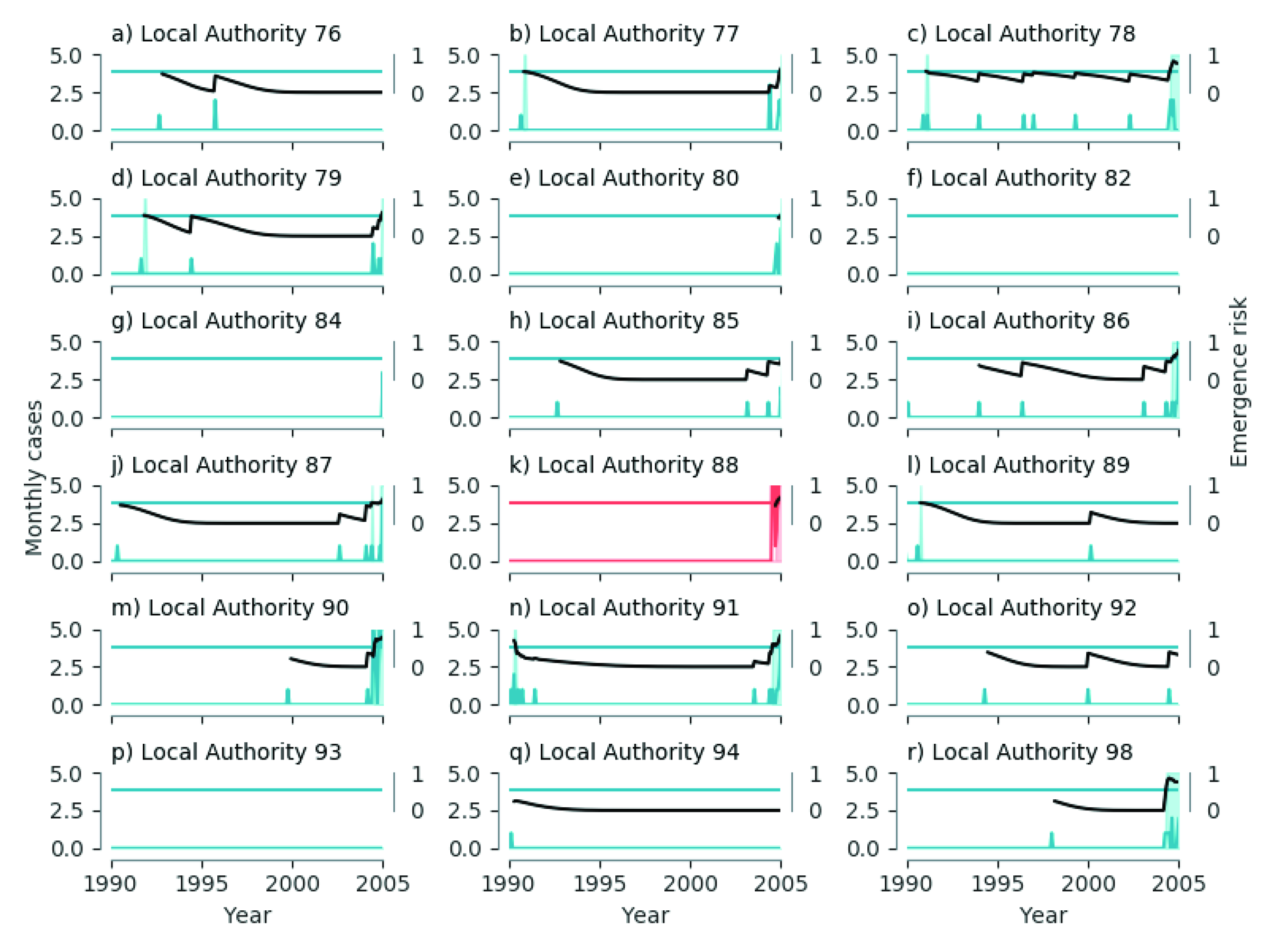

Supplement: S12 Fig — (TIF) [file pbio.3000697.s018.tif]

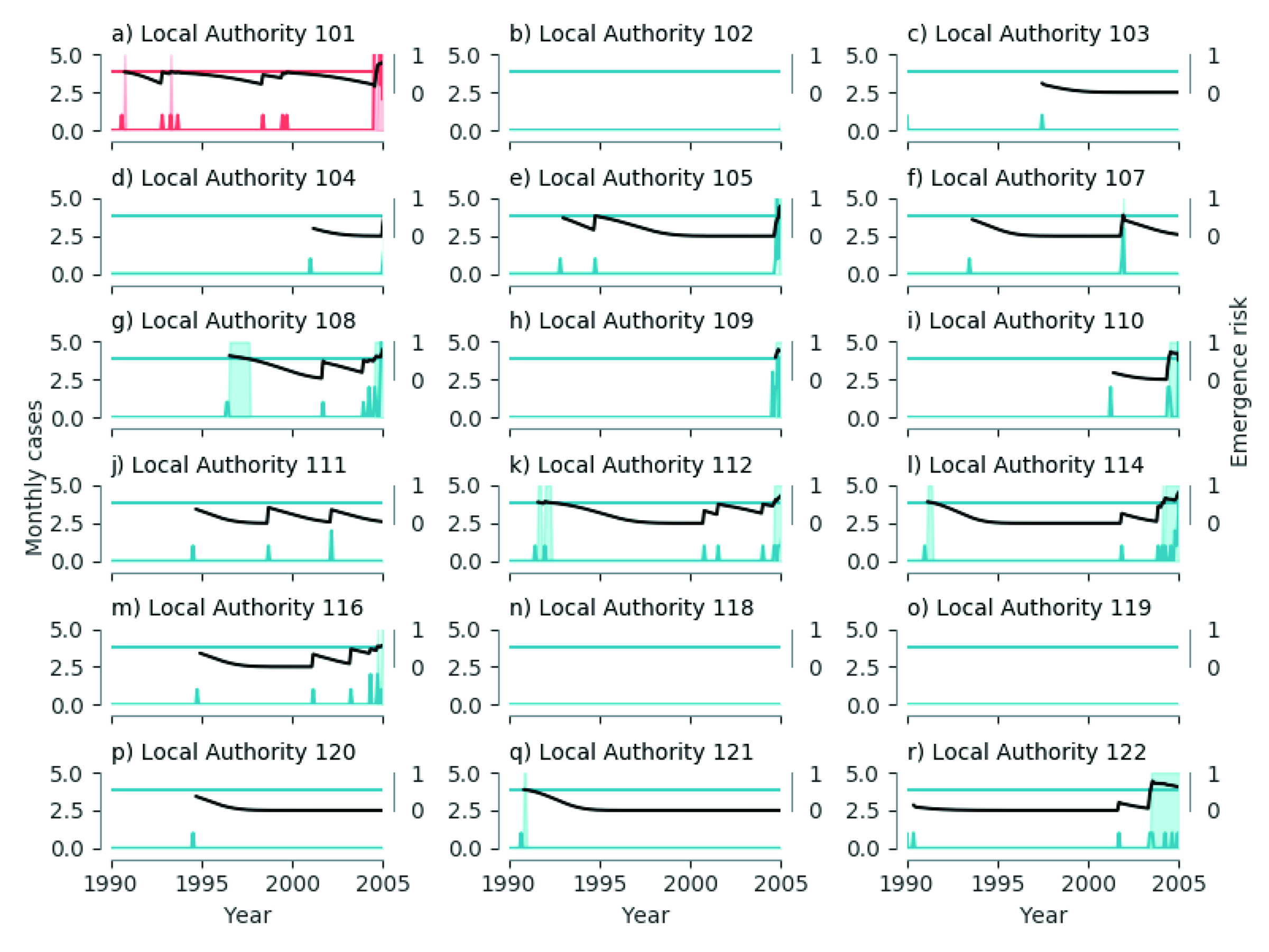

Supplement: S13 Fig — (TIF) [file pbio.3000697.s019.tif]

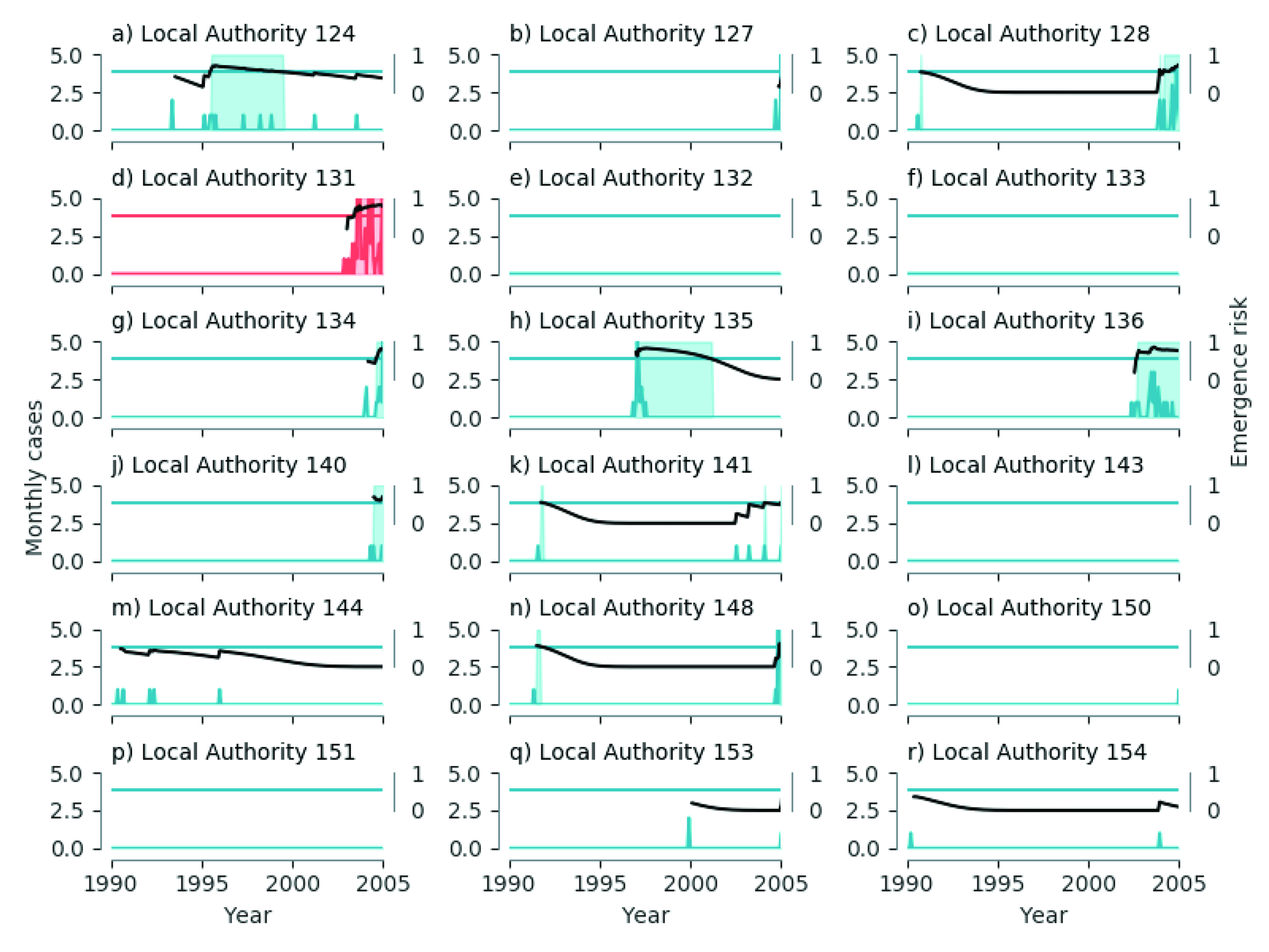

Supplement: S14 Fig — (TIF) [file pbio.3000697.s020.tif]

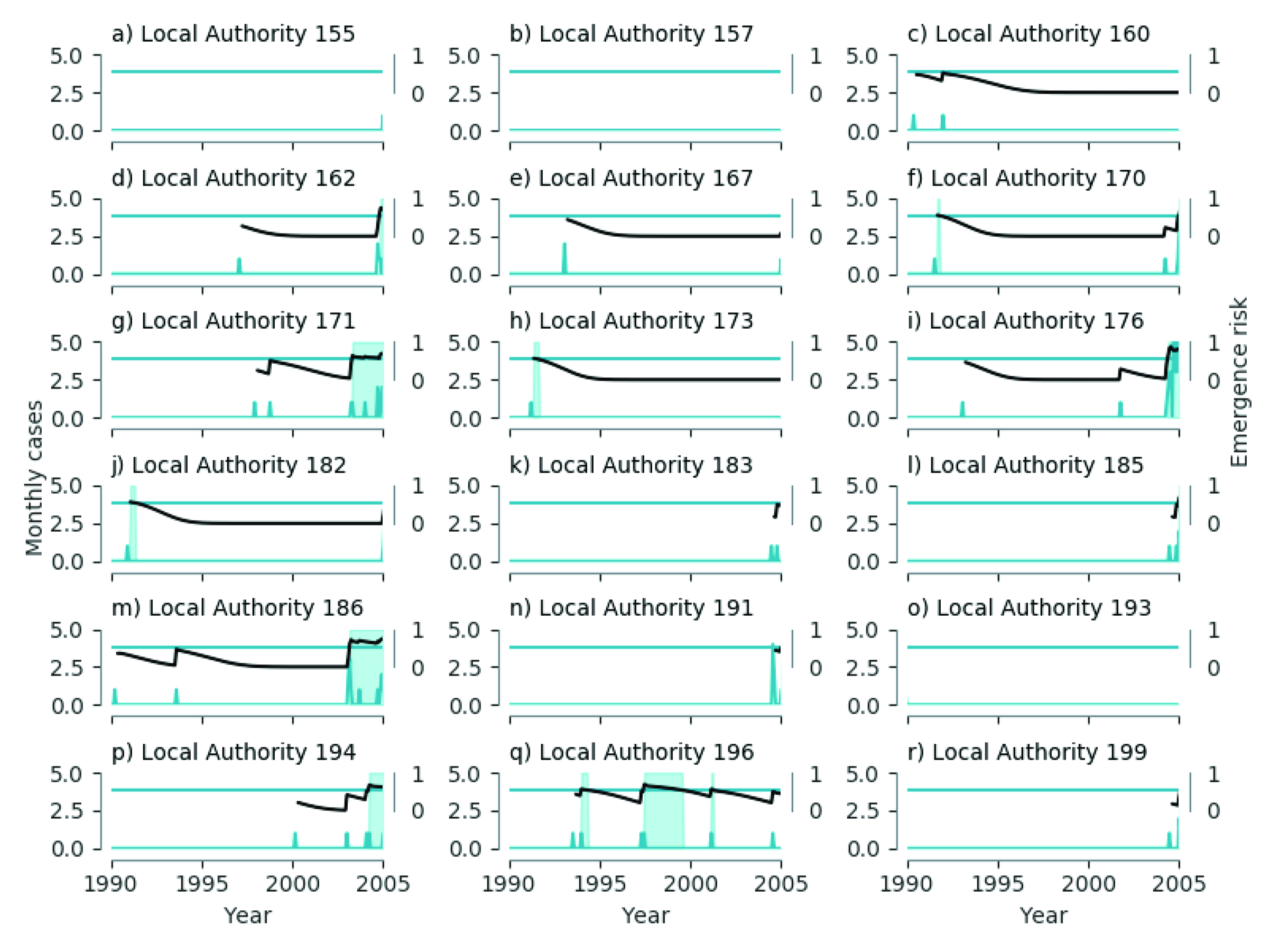

Supplement: S15 Fig — (TIF) [file pbio.3000697.s021.tif]

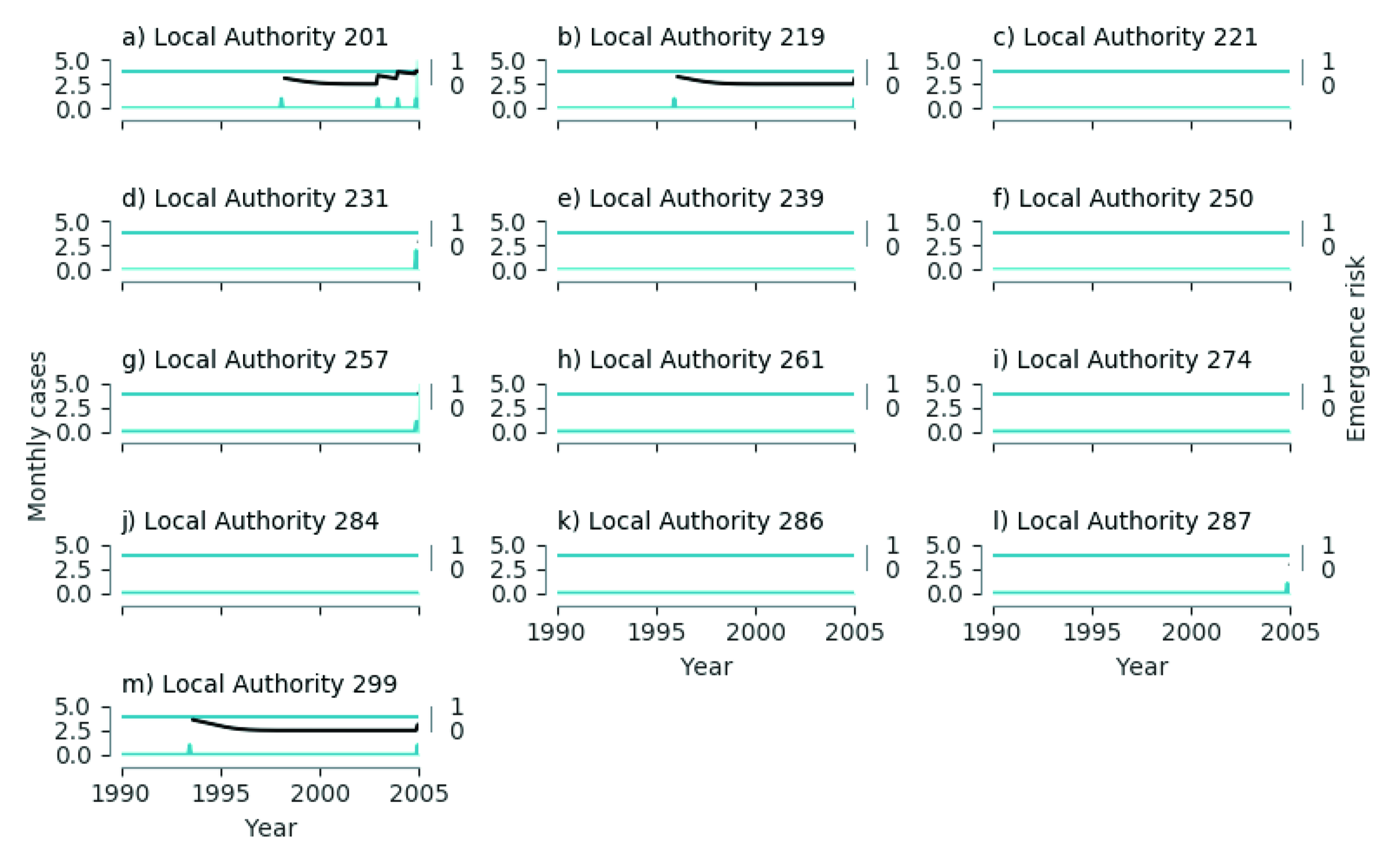

Supplement: S16 Fig — (TIF) [file pbio.3000697.s022.tif]

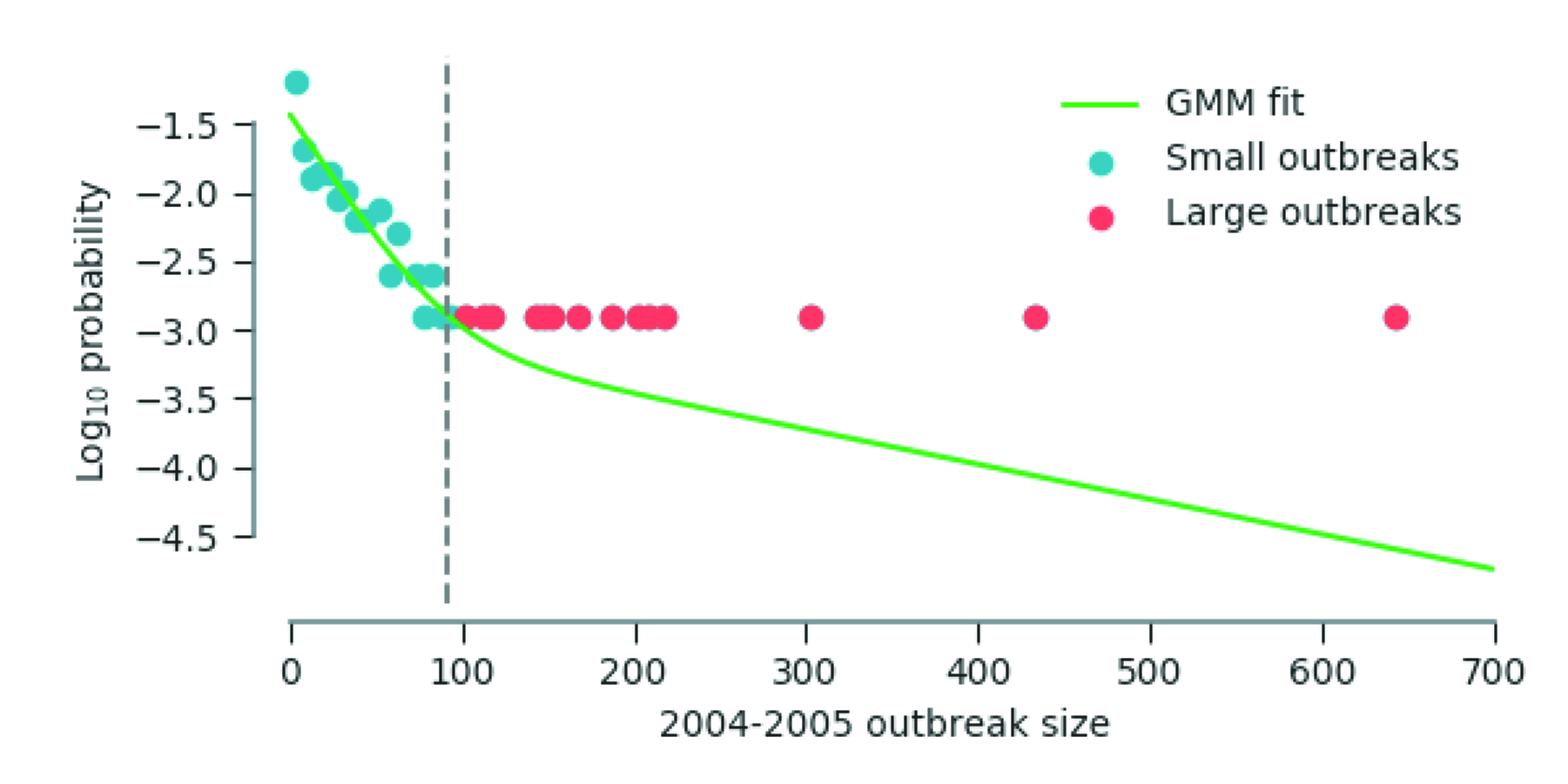

Supplement: S17 Fig — The GMM fit identifies a boundary between large and small outbreaks at 92 cases (dashed gray line). Dots show the empirical distribution calculated using a bin width of 5. Data and code used to generate this figure can be found at https://doi.org/10.5281/zenodo.3713381. GMM, general mixture model. (TIF) [file pbio.3000697.s023.tif]

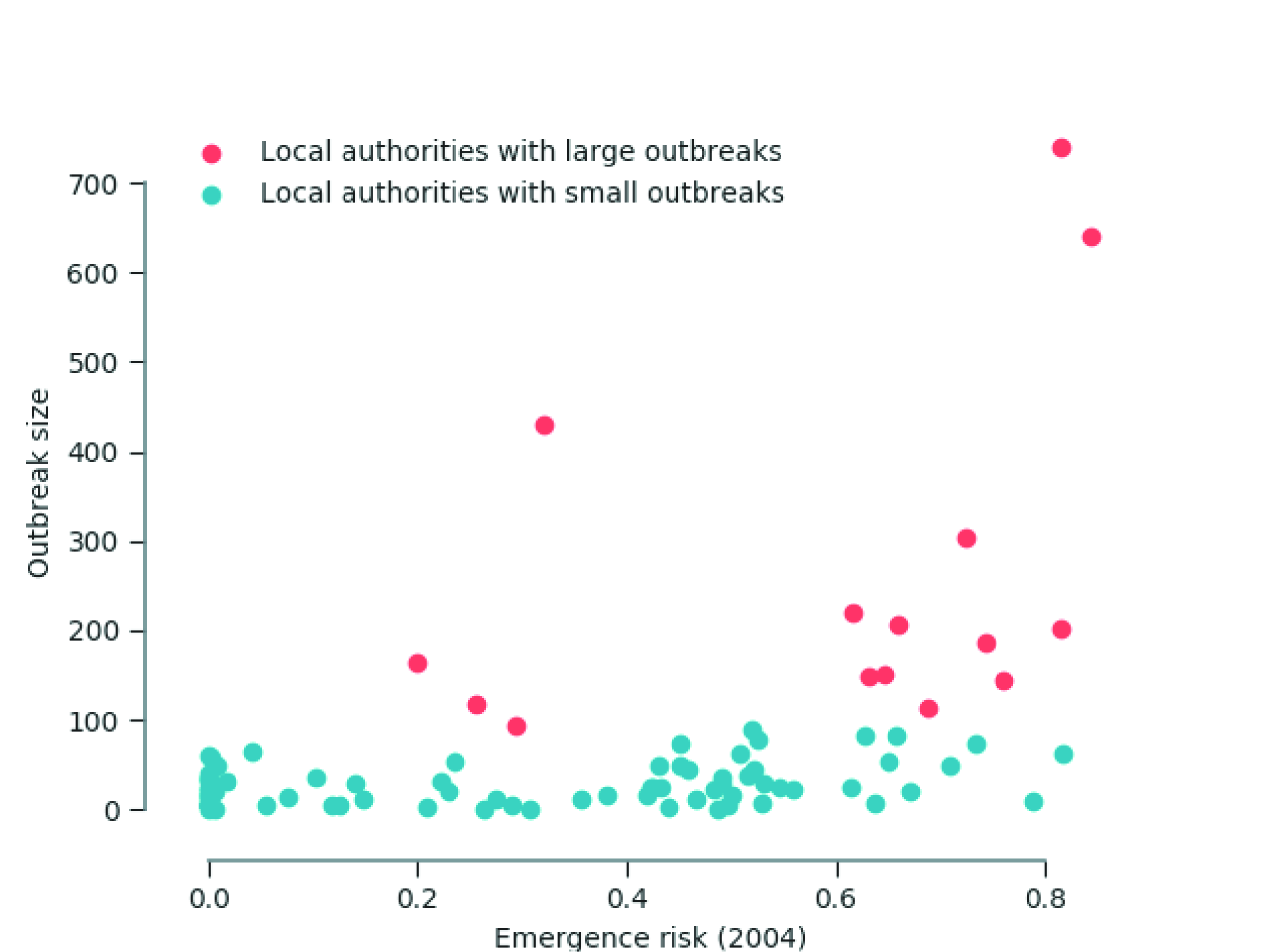

Supplement: S18 Fig — Data and code used to generate this figure can be found at https://doi.org/10.5281/zenodo.3713381. (TIF) [file pbio.3000697.s024.tif]

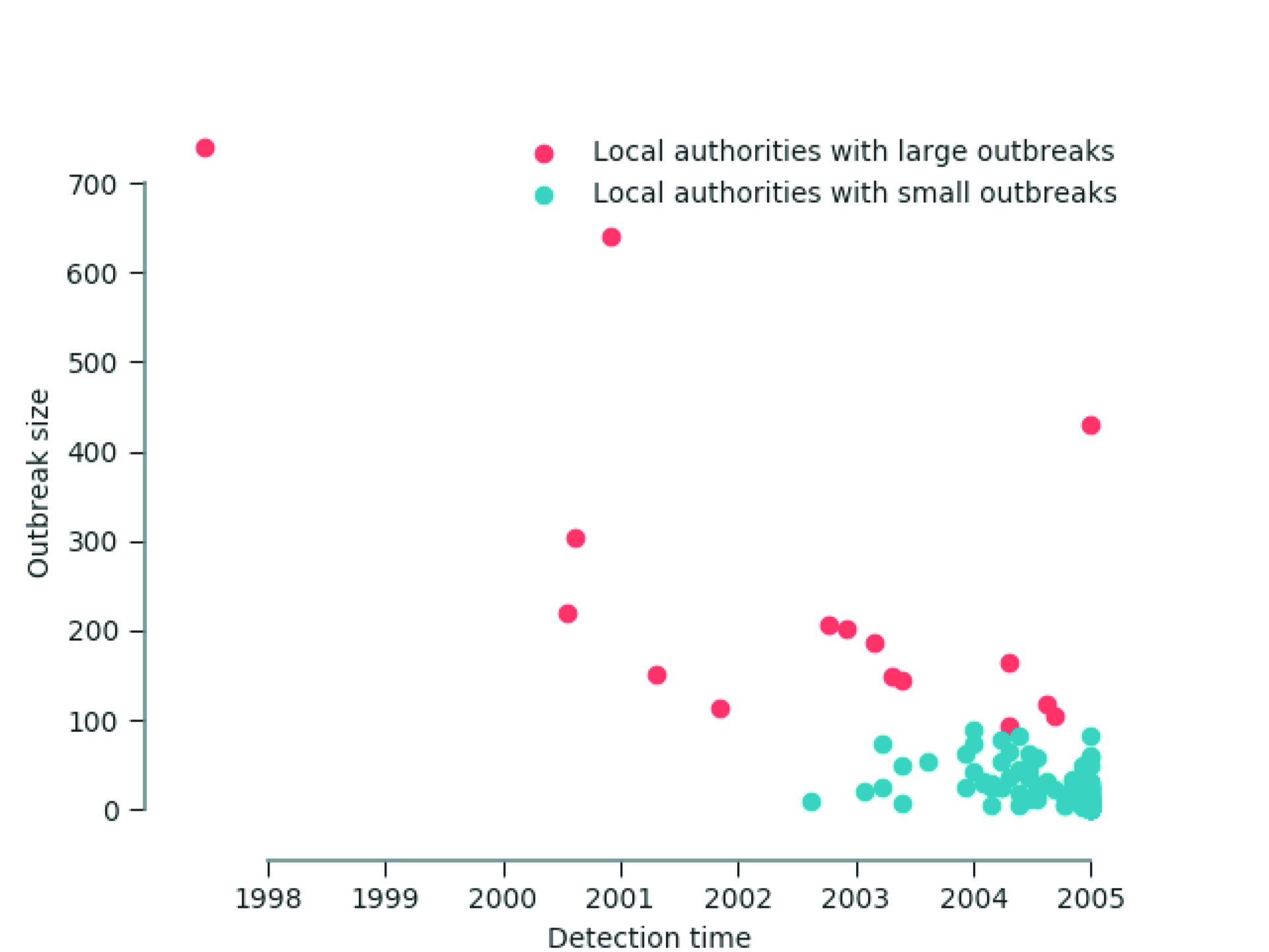

Supplement: S19 Fig — Data and code used to generate this figure can be found at https://doi.org/10.5281/zenodo.3713381. (TIF) [file pbio.3000697.s025.tif]

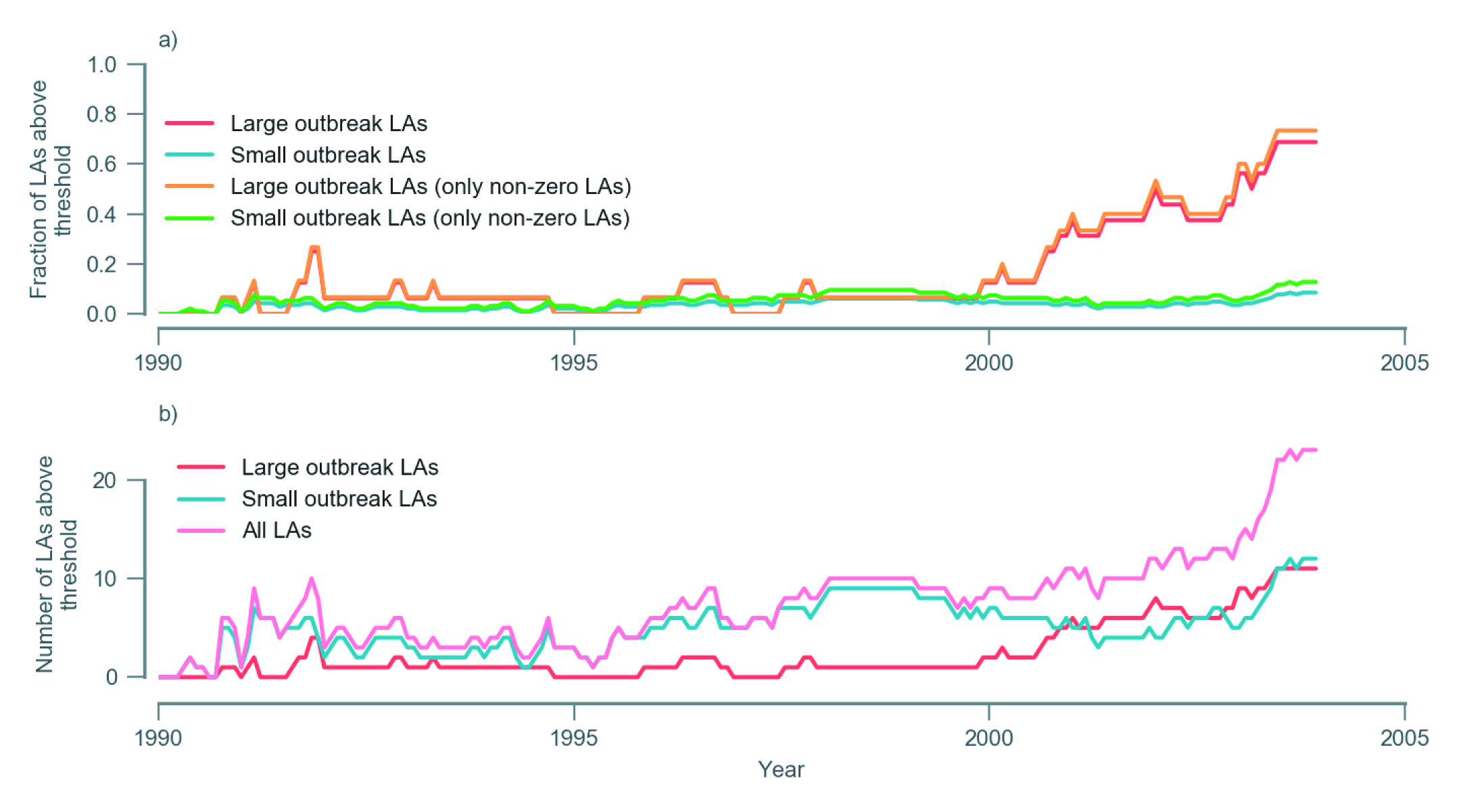

Supplement: S20 Fig — (a) Fraction of LAs above the detection threshold through time. Excluding LAs for which no cases were recorded between 1990 to 2004 (32 in total) has little effect on either fraction. (b) Number of LAs above the detection threshold through time. Data and code used to generate this figure can be found at https://doi.org/10.5281/zenodo.3713381. LA, local authority. (TIF) [file pbio.3000697.s026.tif]

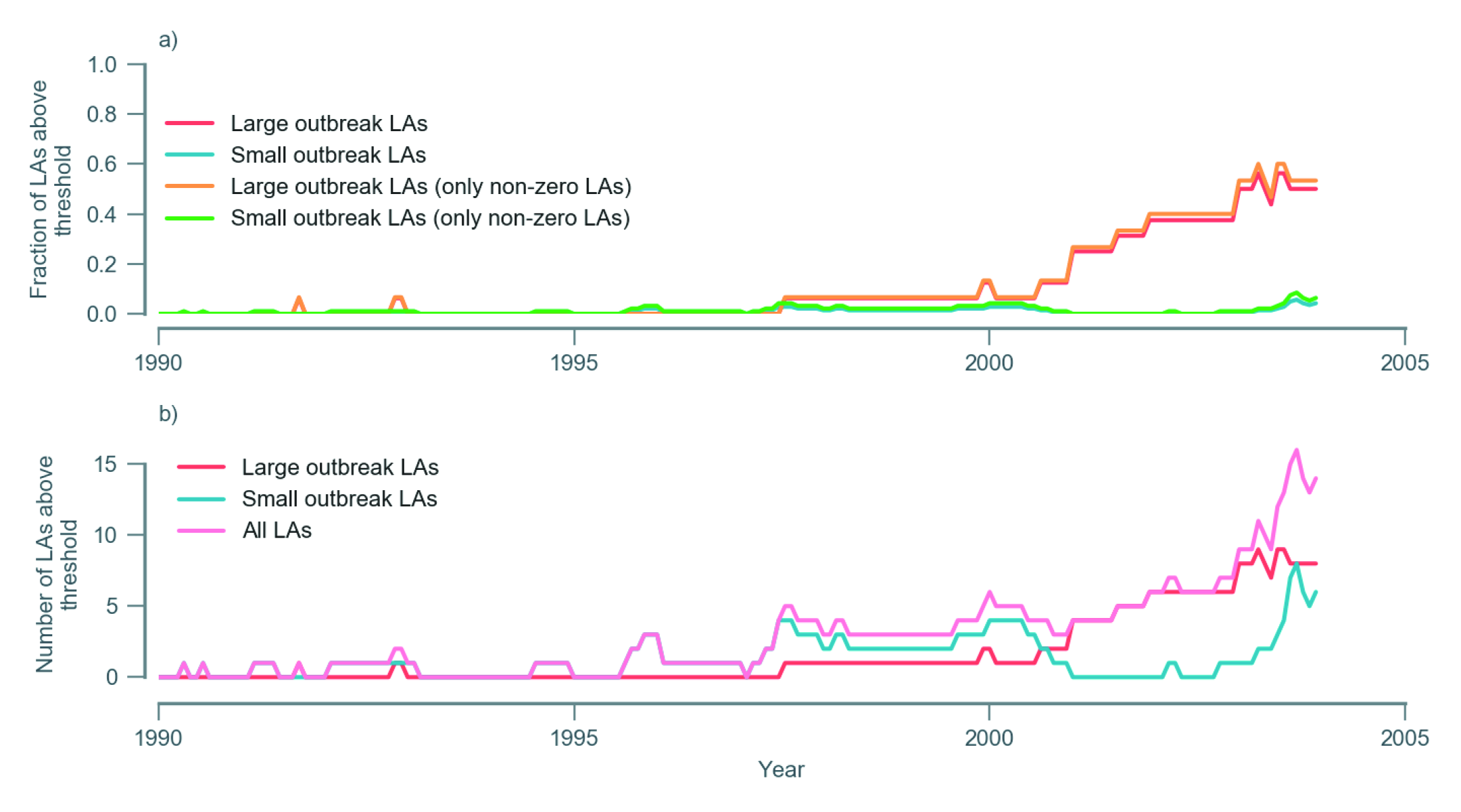

Supplement: S21 Fig — Data and code used to generate this figure can be found at https://doi.org/10.5281/zenodo.3713381. (TIF) [file pbio.3000697.s027.tif]

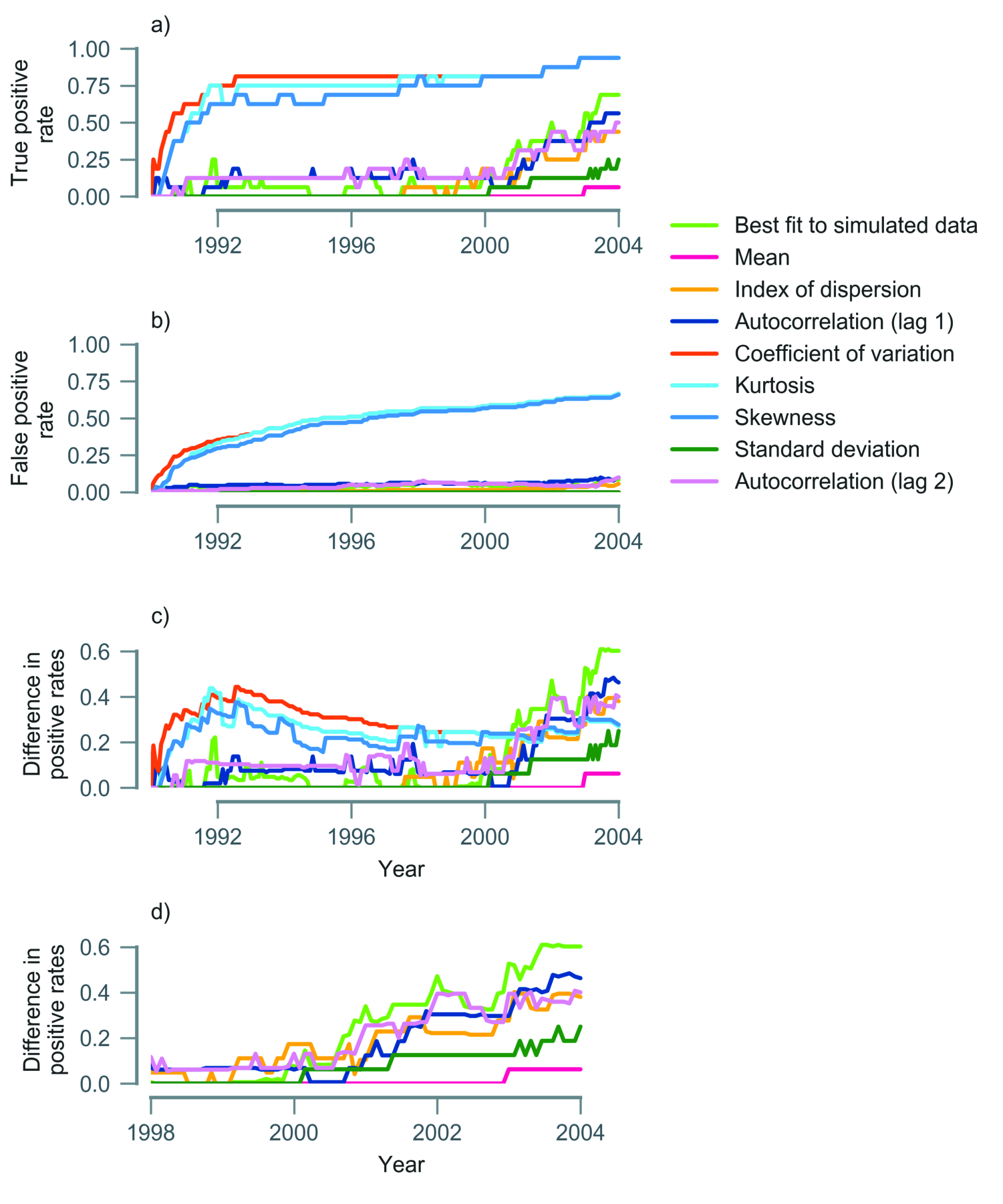

Supplement: S22 Fig — Weights and thresholds for both Dt and the individual EWS were found by fitting to the simulated training data. (a) Detection rate in local authorities with large outbreaks. We assumed that these detections are true positives. (b) False positives, i.e., detections in local authorities with small outbreaks. (c) Difference between the true and false positive rates. (d) Close up of panel (c) focusing on the last 6 years before the outbreak; the coefficient of variation, skewness, and kurtosis are not shown in this panels as these 3 EWSs performed poorly individually—echoing their performance during training (S3 Fig)—with high initial false positive and true positive rates. For all the remaining EWSs, detections increased as the transition was approached, with the fit using all EWSs, Dt, having the highest true positive rate (a) and difference between positive rates (c and d) for most of the period post-2000. Performance of the autocorrelation (at lags 1 and 2) and the index of dispersion are close behind. Data and code used to generate this figure can be found at https://doi.org/10.5281/zenodo.3713381. EWS, early warning signal. (TIF) [file pbio.3000697.s028.tif]

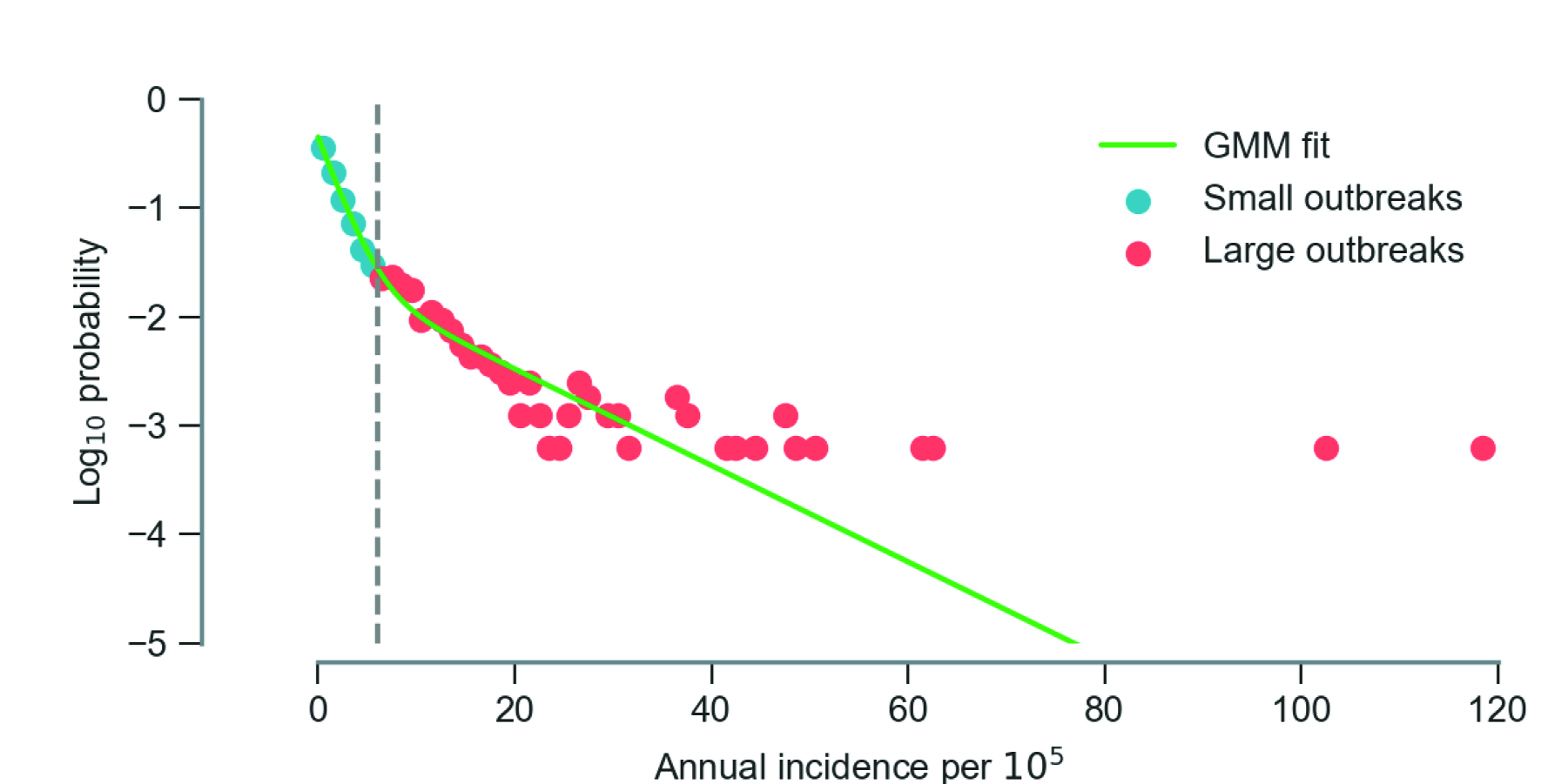

Supplement: S23 Fig — The GMM is fit to the annual incidence data from the years 1980 to 2012, treating each year and state as an independent sample. The fit identifies a boundary between large and small outbreaks of 6.10 cases per 105 (dashed gray line). Dots show the empirical distribution calculated using a bin width of 1 case per 105. Data and code used to generate this figure can be found at https://doi.org/10.5281/zenodo.3713381. GMM, general mixture model. (TIF) [file pbio.3000697.s029.tif]

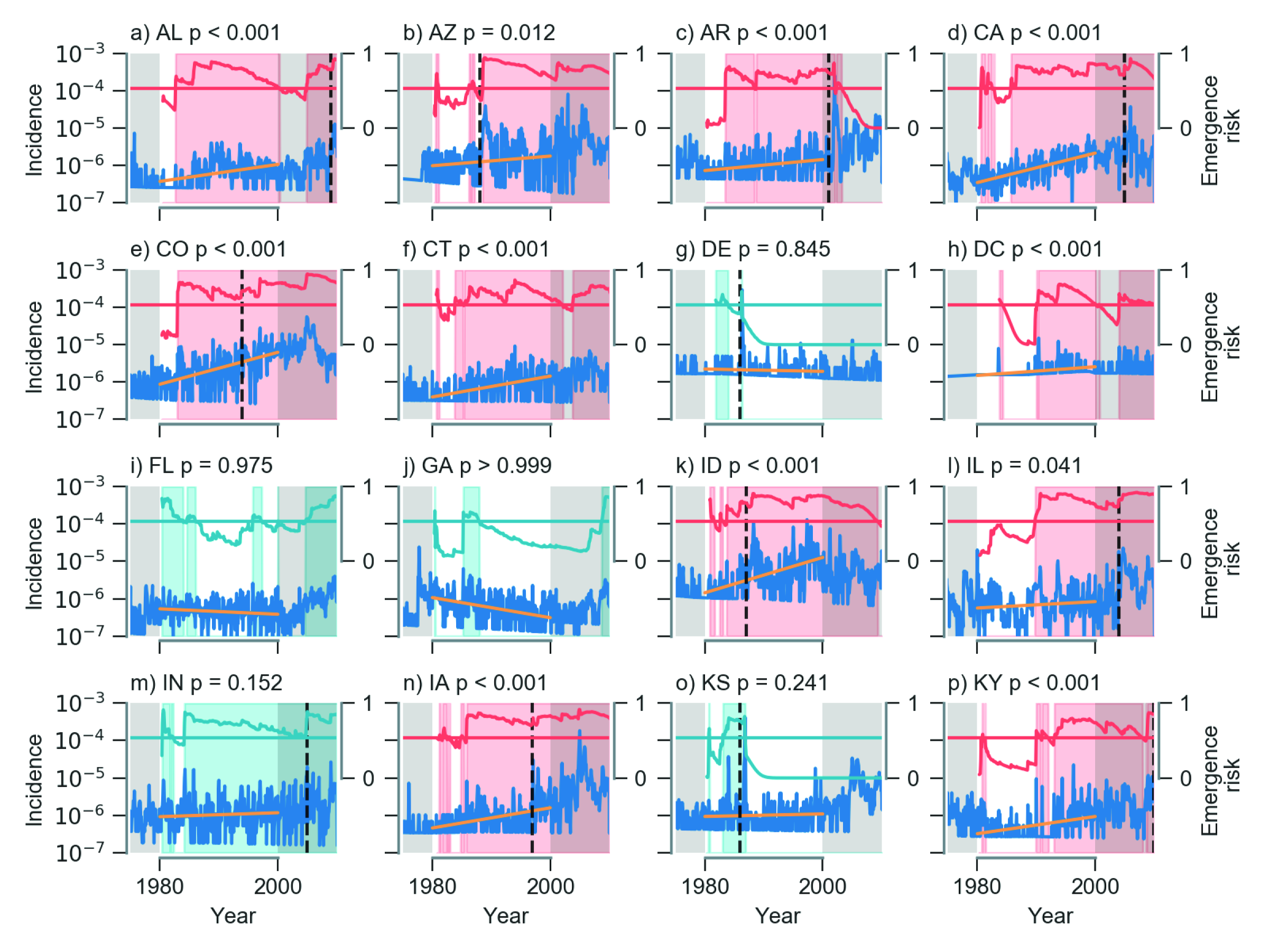

Supplement: S24 Fig — Colors indicate whether the linear regression analysis classified a state as emerging (red) or not (blue). Log-transformed incidence data are shown in dark blue; linear regression fits are shown in orange. p-Values from the linear regression analysis are shown in the panel labels. Shaded backgrounds indicate Dt > c. Black dashed vertical lines indicate the year of the first large outbreak, found using the GMM. Data and code used to generate this figure can be found at https://doi.org/10.5281/zenodo.3713381. GMM, general mixture model. (TIF) [file pbio.3000697.s030.tif]

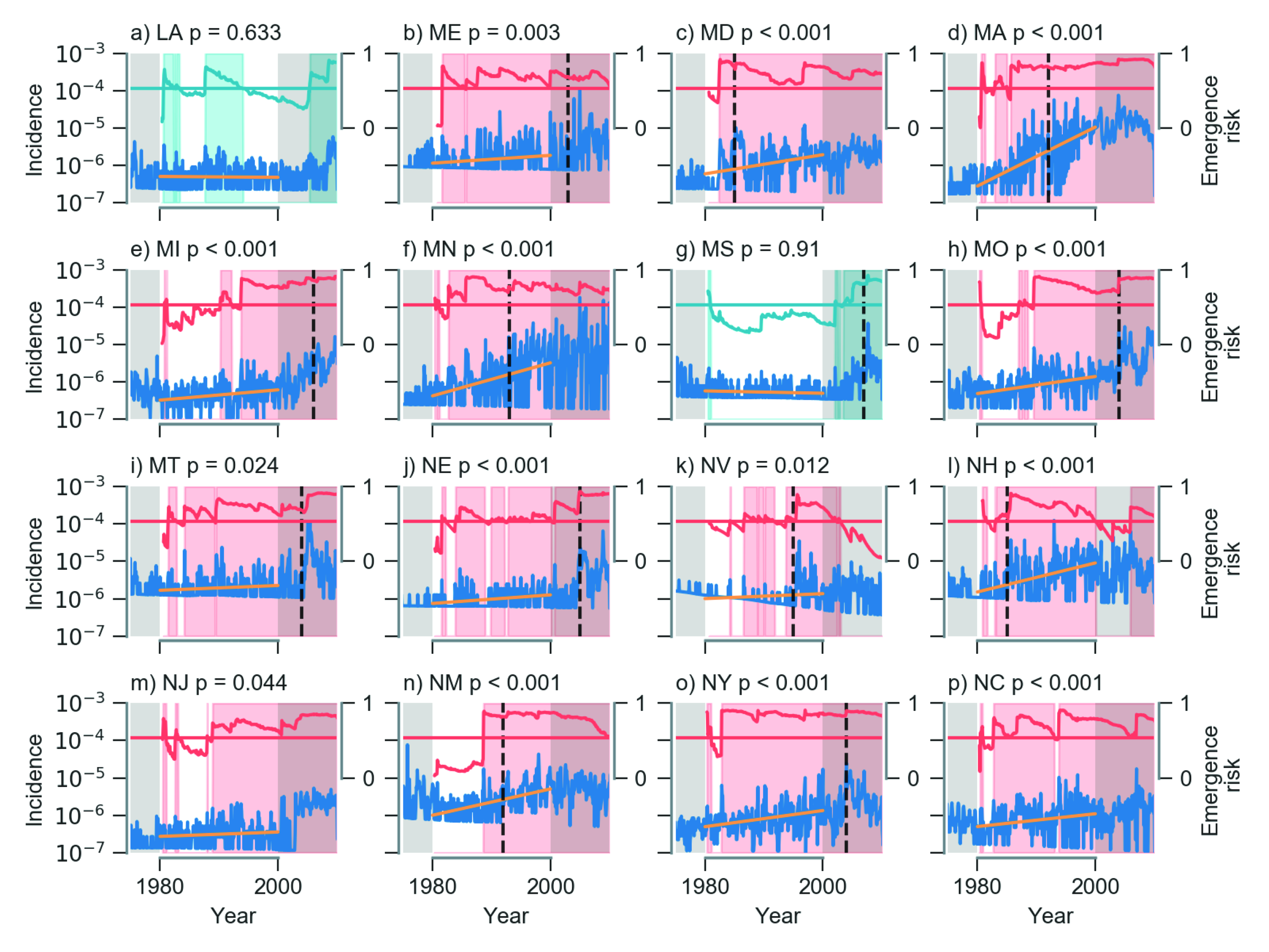

Supplement: S25 Fig — (TIF) [file pbio.3000697.s031.tif]

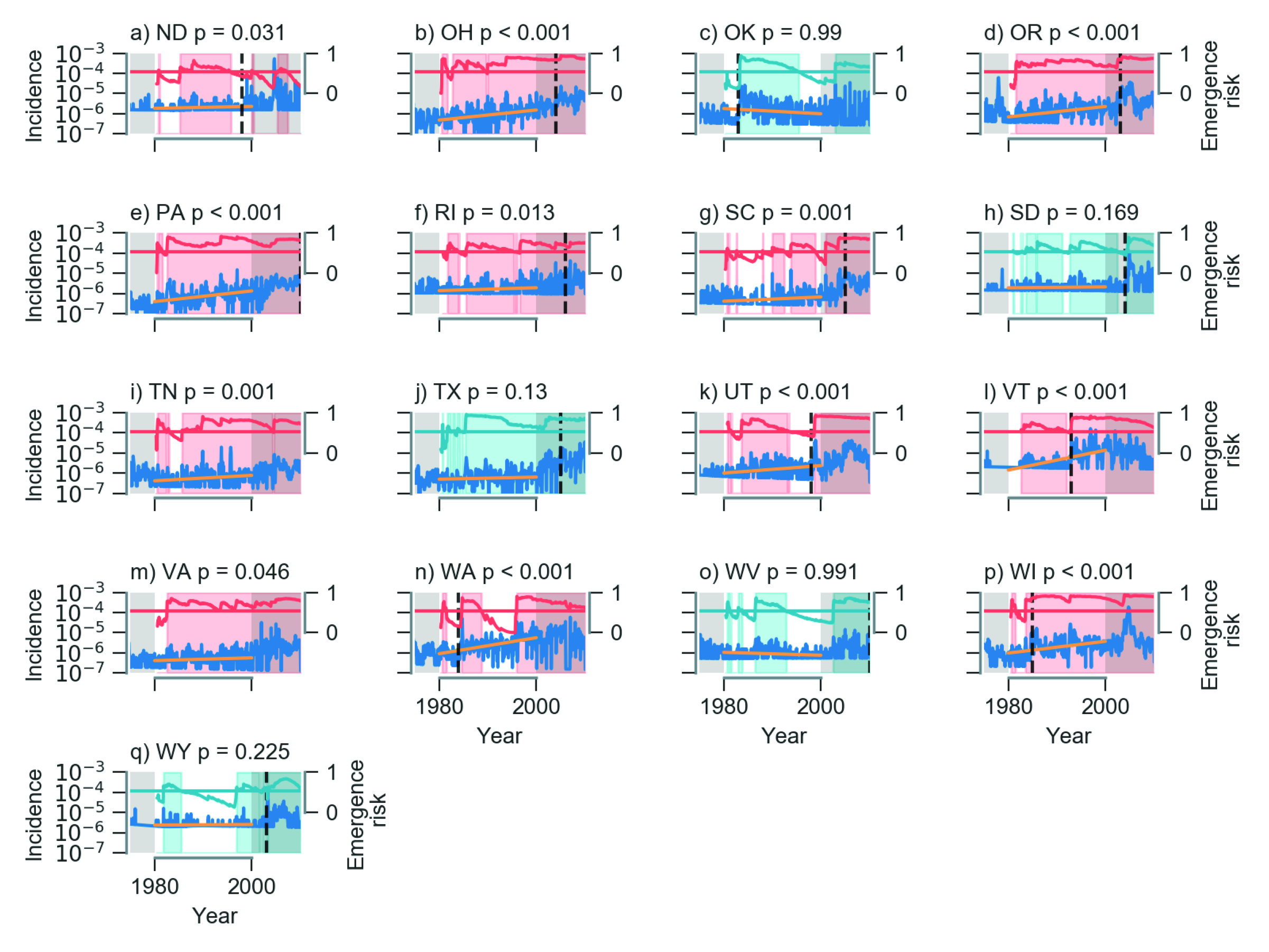

Supplement: S26 Fig — (TIF) [file pbio.3000697.s032.tif]

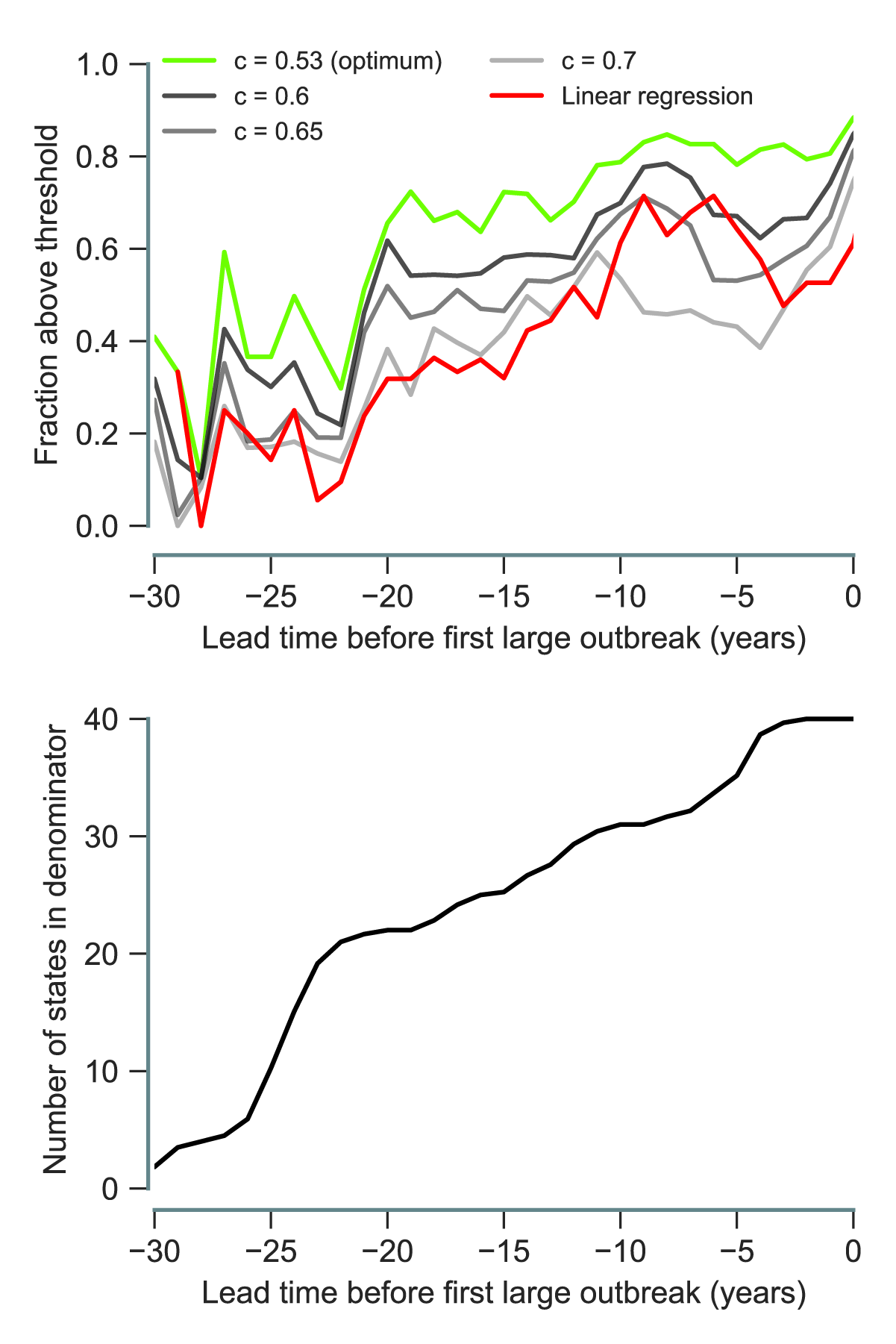

Supplement: S27 Fig — (a) Fraction of states above the detection threshold for a range of detection thresholds as a function of lead time to the first large outbreak (determined using the GMM). Also shown are the number of states with p < 0.05 when using the linear regression method. (b) Because the earliest year used to calculate the EWSs is 1980, the number of states in the denominator of the fraction decreases as the lead time increases, with states that have the earliest first large outbreaks dropping out first. This leads to difficulties interpreting the results shown in panel (a) at lead times greater than 4 years. Data and code used to generate this figure can be found at https://doi.org/10.5281/zenodo.3713381. EWS, early warning signal. (TIF) [file pbio.3000697.s033.tif]

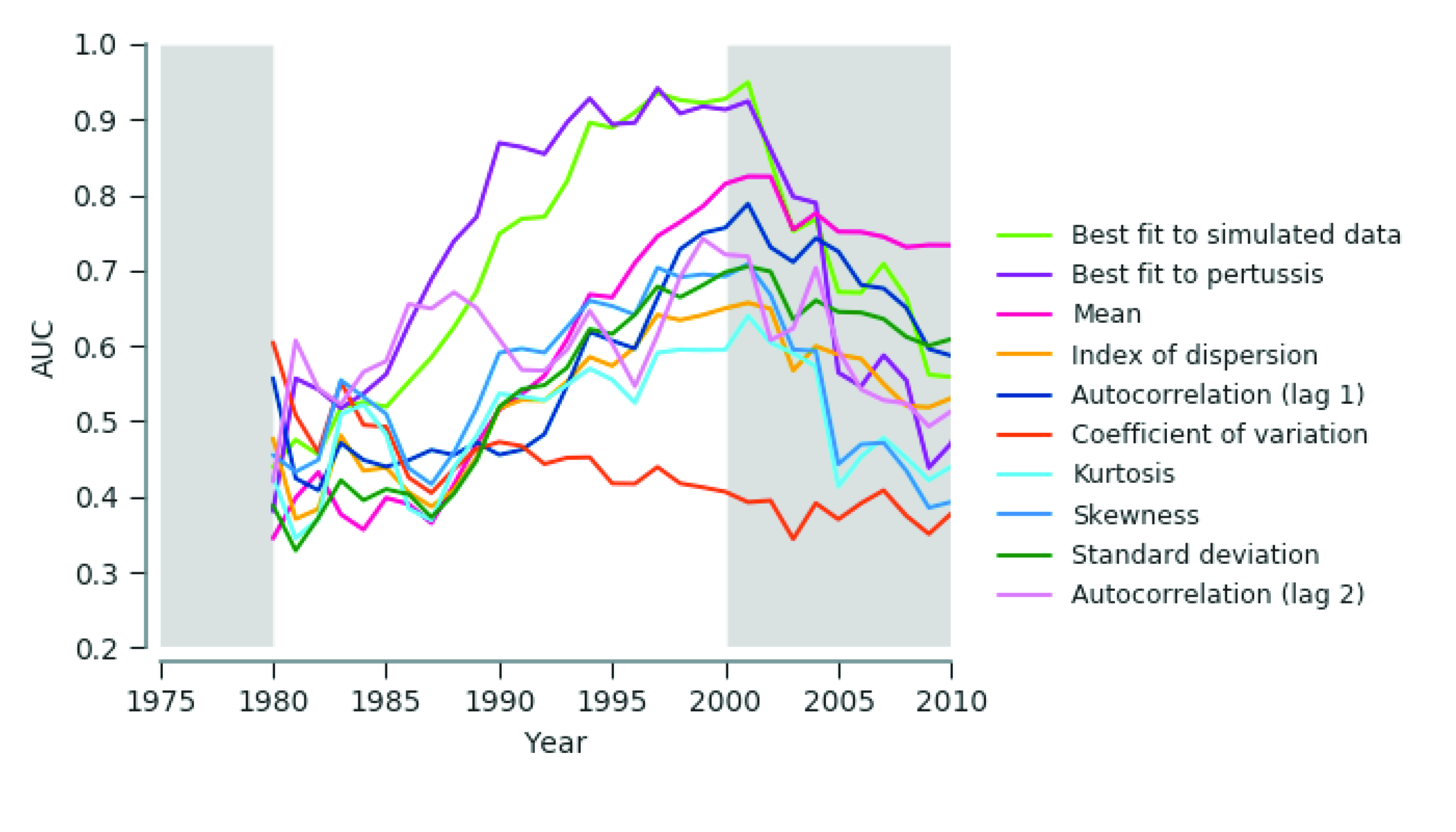

Supplement: S28 Fig — The fit to simulated data performs much better than any individual EWS and is close to the upper bound on the ability of our set of EWS to classify pertussis emergence in the US. Data and code used to generate this figure can be found at https://doi.org/10.5281/zenodo.3713381. EWS, early warning signal. (TIF) [file pbio.3000697.s034.tif]

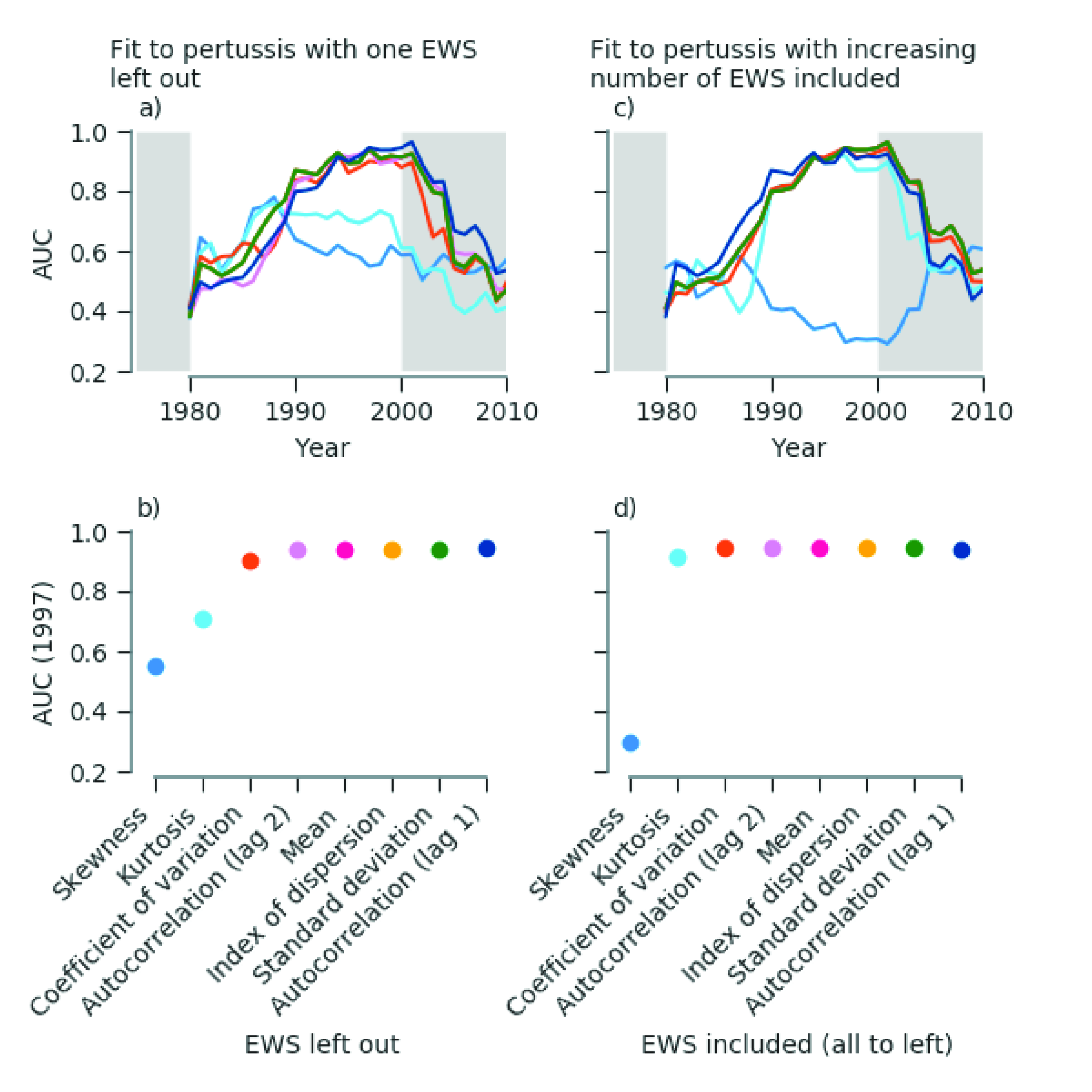

Supplement: S29 Fig — The skewness, kurtosis, and coefficient of variation remain the most important to the performance of Dt. Data and code used to generate this figure can be found at https://doi.org/10.5281/zenodo.3713381. (TIF) [file pbio.3000697.s035.tif]

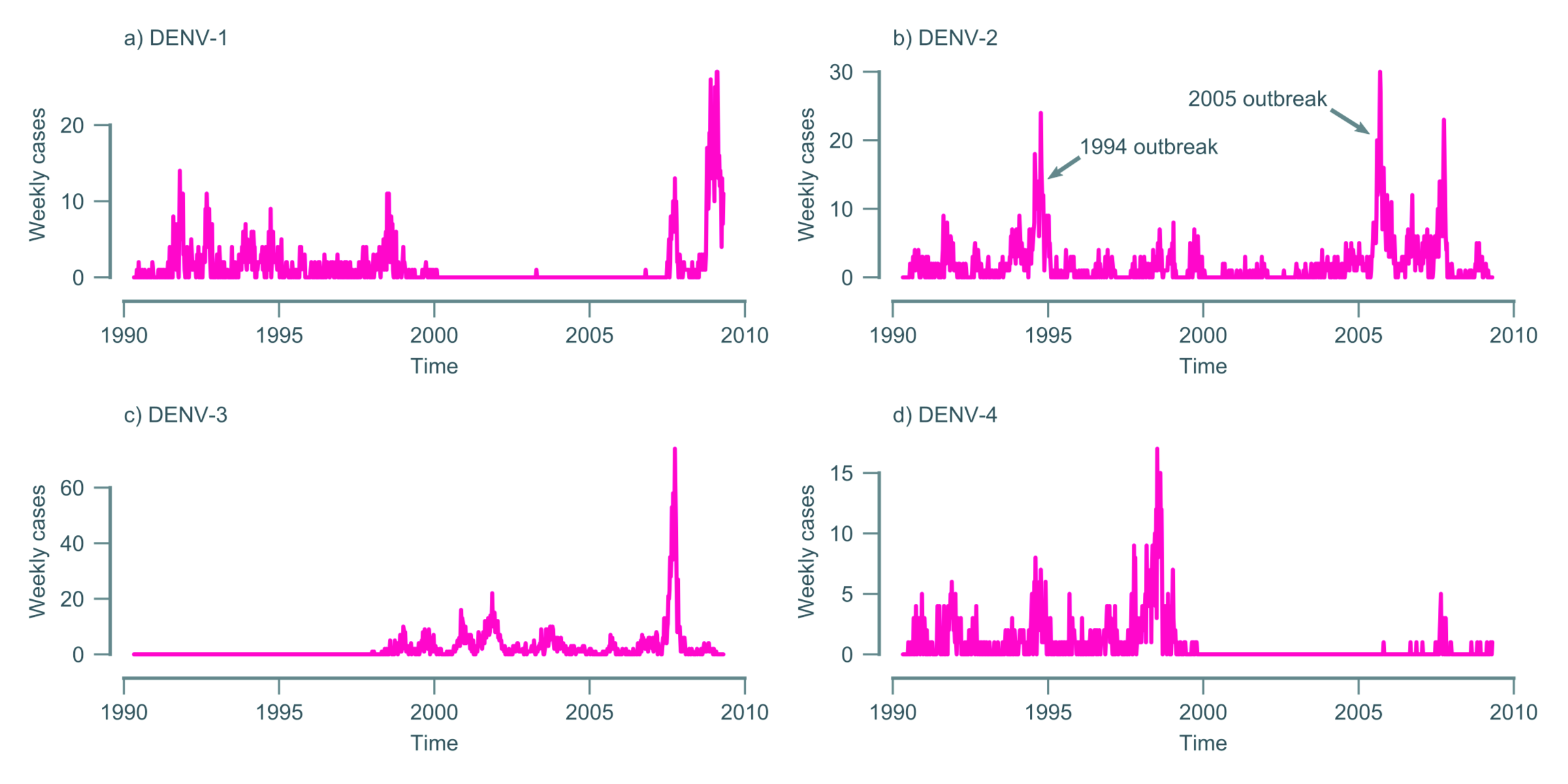

Supplement: S30 Fig — Data and code used to generate this figure can be found at https://doi.org/10.5281/zenodo.3713381. DENV, dengue virus. (TIF) [file pbio.3000697.s036.tif]

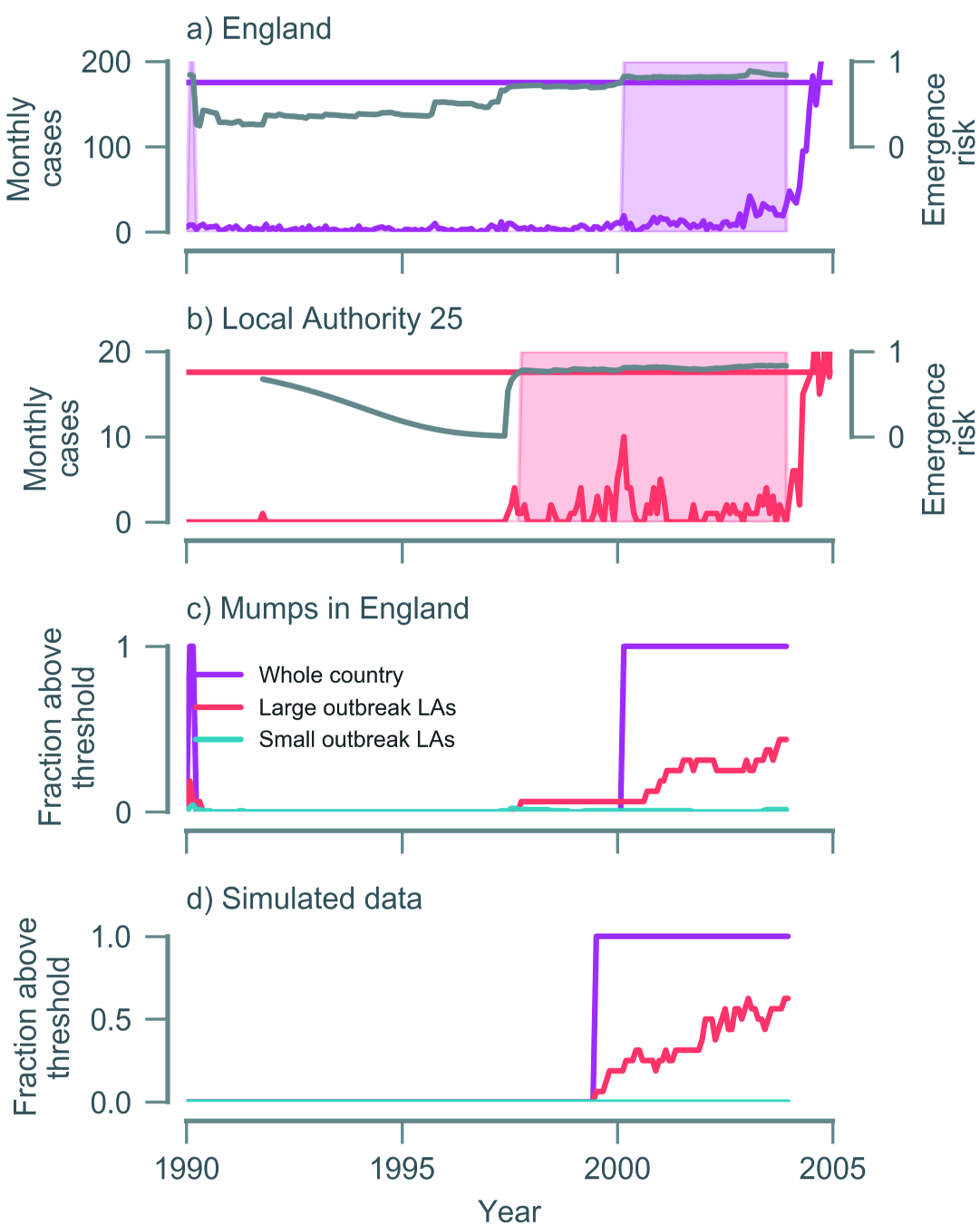

Supplement: S31 Fig — Instead of training on the simulated data, we take the weights and detection threshold found by training on the pertussis data. We only included the 3 EWSs that were most important to the performance of Dt in the fit (see S4 Fig), namely, the coefficient of variation, the kurtosis, and the skewness. On the national level, the detection time is largely unchanged. On the local authority level, both true and false positives are reduced, indicating a more stringent detection threshold. Performance is slightly worse if the fit to pertussis including all EWSs is used. Data and code used to generate this figure can be found at https://doi.org/10.5281/zenodo.3713381. EWS, early warning signal. (TIF) [file pbio.3000697.s037.tif]

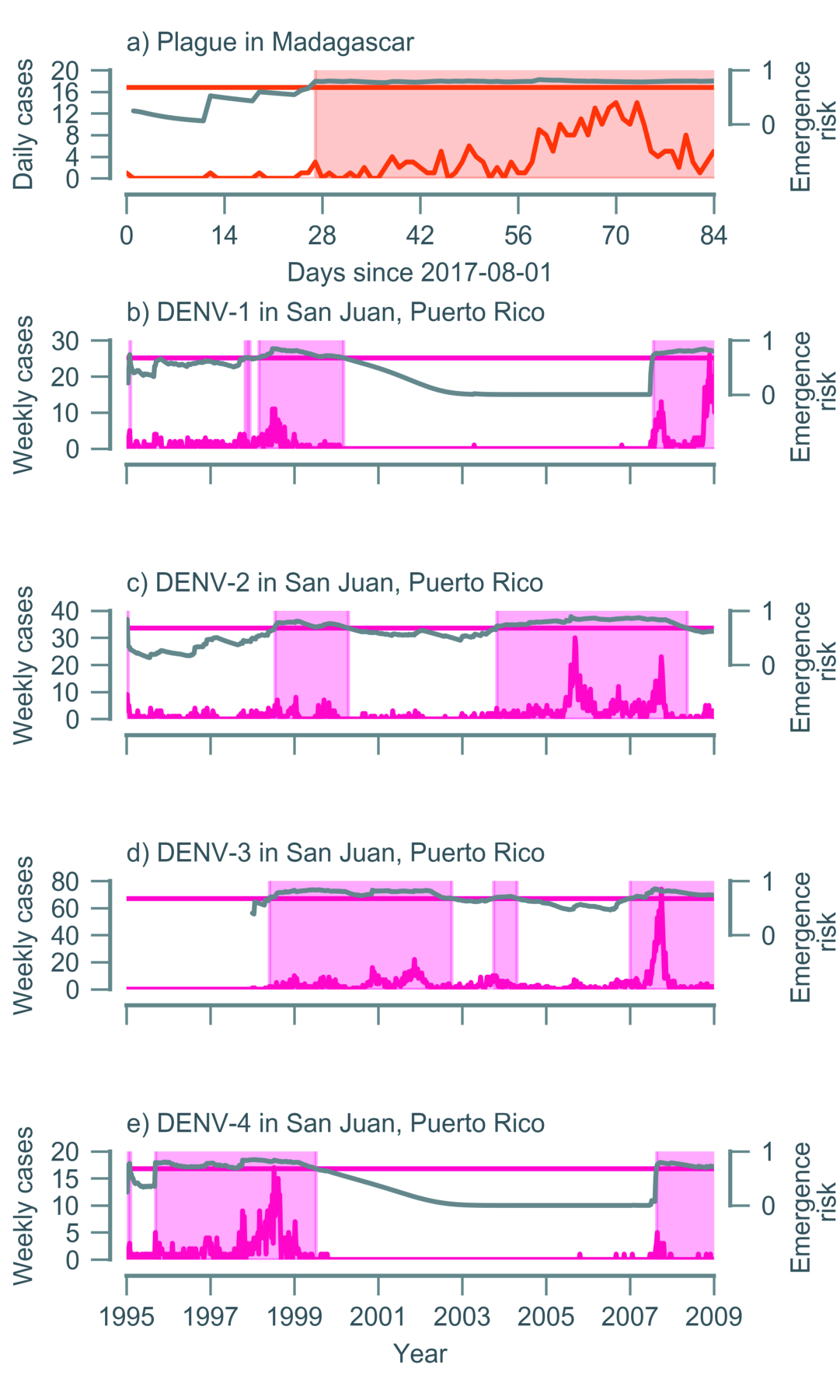

Supplement: S32 Fig — We see no effect on the timing of detection for bubonic plague and a very small effect for dengue. Data and code used to generate this figure can be found at https://doi.org/10.5281/zenodo.3713381. (TIF) [file pbio.3000697.s038.tif]

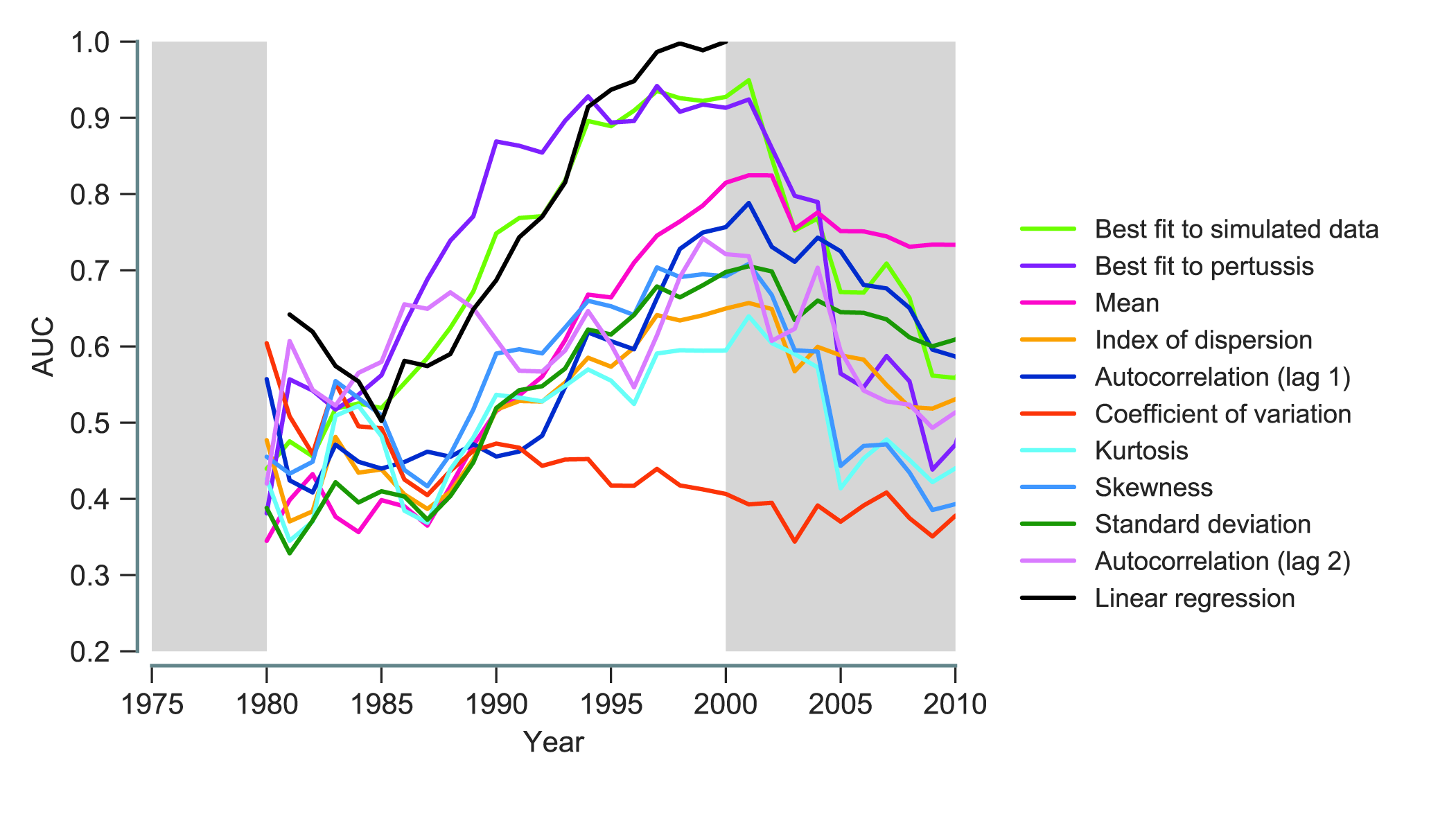

Supplement: S33 Fig — Linear regression is performed using the log-transformed pertussis data from 1980 to the date indicated on the x-axis. As the endpoint of the linear regression approaches 2000, the AUC approaches 1. This is to be expected, as we are comparing the performance of the linear regression with itself. The EWS-based approach fitted to simulated data tracks this performance closely until the mid-1990s, when the AUC saturates at 0.9. The EWS-based approach fitted to the pertussis data outperforms the linear regression during much of the 1980s and 90s, because of the 8-dimensional logistic model’s increased flexibility. Data and code used to generate this figure can be found at https://doi.org/10.5281/zenodo.3713381. AUC, area under the receiver-operator characteristic curve; EWS, early warning signal. (TIF) [file pbio.3000697.s039.tif]

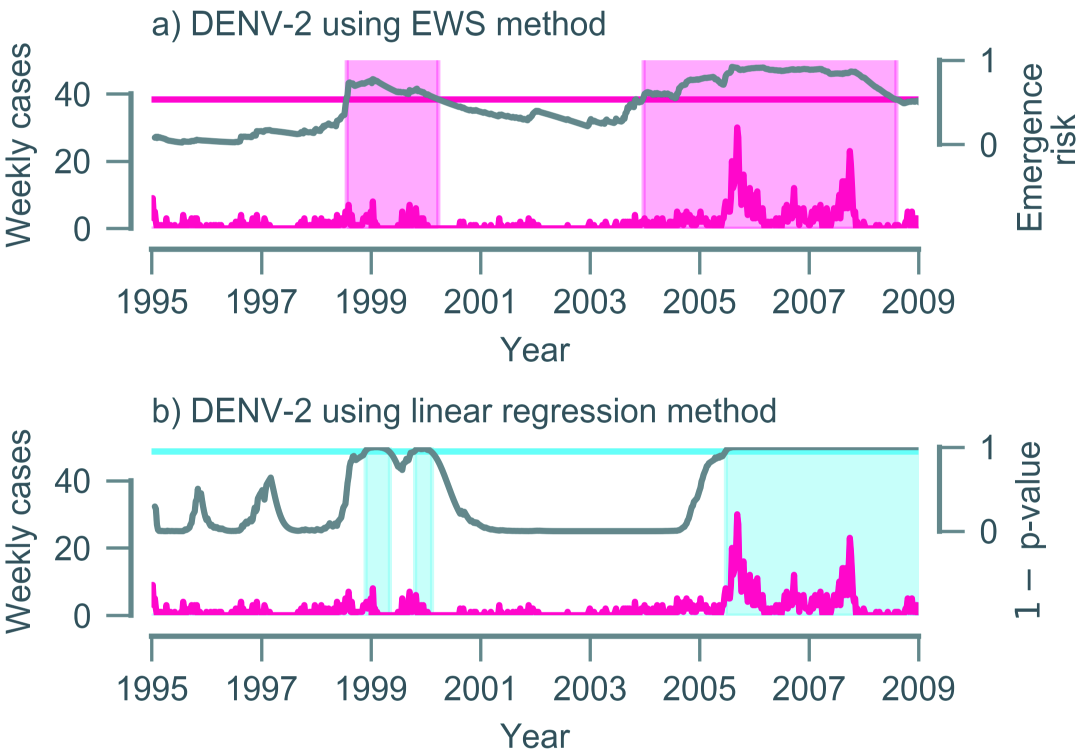

Supplement: S34 Fig — The linear regression method was implemented in the same way as for the pertussis case study and was calculated using all log-transformed case reports from January 1, 1995 up to the week indicated. As can be seen by visually inspecting the curves in panel (b), even if the significance level (i.e., the detection threshold, indicated by the horizontal line) is lowered, the linear regression method performs worse at detecting the outbreak. Data and code used to generate this figure can be found at https://doi.org/10.5281/zenodo.3713381. DENV, dengue virus; EWS, early warning signal. (TIF) [file pbio.3000697.s040.tif]
